# Luminal long non-coding RNAs regulated by estrogen receptor alpha in a ligand-independent manner show functional roles in breast cancer

**DOI:** 10.18632/oncotarget.6420

**Published:** 2015-11-28

**Authors:** Valentina Miano, Giulio Ferrero, Stefania Reineri, Livia Caizzi, Laura Annaratone, Laura Ricci, Santina Cutrupi, Isabella Castellano, Francesca Cordero, Michele De Bortoli

**Affiliations:** ^1^ Center for Molecular Systems Biology, University of Turin, Turin, Italy; ^2^ Department of Clinical and Biological Sciences, University of Turin, Turin, Italy; ^3^ Department of Computer Science, University of Turin, Turin, Italy; ^4^ Bioindustry Park Silvano Fumero, Turin, Italy; ^5^ Department of Medical Sciences, University of Turin, Turin, Italy; ^6^ Department of Molecular Biology, Max Planck Institute for Biophysical Chemistry, Göttingen, Germany

**Keywords:** lncRNA, breast cancer, estrogen receptor, data integration, DSCAM-AS1

## Abstract

Estrogen Receptor alpha (ERα) activation by estrogenic hormones induces luminal breast cancer cell proliferation. However, ERα plays also important hormone-independent functions to maintain breast tumor cells epithelial phenotype. We reported previously by RNA-Seq that in MCF-7 cells in absence of hormones ERα down-regulation changes the expression of several genes linked to cellular development, representing a specific subset of estrogen-induced genes. Here, we report regulation of long non-coding RNAs from the same experimental settings. A list of 133 Apo-ERα-Regulated lncRNAs (AER-lncRNAs) was identified and extensively characterized using published data from cancer cell lines and tumor tissues, or experiments on MCF-7 cells. For several features, we ran validation using cell cultures or fresh tumor biopsies. AER-lncRNAs represent a specific subset, only marginally overlapping estrogen-induced transcripts, whose expression is largely restricted to luminal cells and which is able to perfectly classify breast tumor subtypes. The most abundant AER-lncRNA, DSCAM-AS1, is expressed in ERα+ breast carcinoma, but not in pre-neoplastic lesions, and correlates inversely with EMT markers. Down-regulation of DSCAM-AS1 recapitulated, in part, the effect of silencing ERα, i.e. growth arrest and induction of EMT markers. In conclusion, we report an ERα-dependent lncRNA set representing a novel luminal signature in breast cancer cells.

## INTRODUCTION

Breast tumors of the luminal subtype expressing Estrogen Receptor alpha (ERα) represent a prominent part of breast cancers and are treated with anti-estrogenic drugs with good rate of success, albeit endocrine resistance is still difficult to detect and justifies failure in one-fourth of cases [1]. In addition to being the main mediator of estrogenic hormone action in breast cancer cells, ERα displays an estrogen-independent function in its unliganded status (Apo-ERα). The functions of ERα as well as of other nuclear receptors in absence of ligands have been exhaustively reviewed recently [2, 3] and are thought to depend on phosphorylation by several signal transducing kinases or on interaction with other Transcription Factors (TFs). In breast cancer cells cultured in absence of hormone, depletion of ERα brings about a response similar to Epithelial-to-Mesenchymal Transition (EMT) [4–6] by activating mesenchymal genes and growth-sustaining pathways and, *in vivo*, the loss of ERα is usually accompanied by a more invasive and clinically aggressive phenotype [7, 8]. Conversely, in some model systems, it was shown that re-expression of ERα leads to the re-appearance of epithelial gene expression [5, 9]. Thus, together with other TFs such as Forkhead box protein A1 (FoxA1) and Activating enhancer binding Protein 2 γ (AP-2γ), the estrogen-independent activity of ERα seems important in maintaining the luminal epithelial phenotype and blocking EMT in breast cancer cells. It is noteworthy that a hormone-deprived environment is what is realized in breast cancer patients treated with Aromatase Inhibitors (AIs), the category of drugs that is becoming one of the most widely used for breast cancer patients with ERα+ tumors. In our previous work we have shown that unliganded ERα binds to thousands of chromatin sites and controls the basal transcription of genes linked to cell development and epithelial differentiation, which represent a specific subset of estrogen-induced genes [4]. Interestingly, among Apo-ERα-dependent genes a group of noncoding transcripts was observed. Long noncoding RNAs (lncRNAs) are increasingly recognized as an exceptionally interesting group of RNAs with regulatory functions. LncRNAs show a much higher degree of tissue- and cell-type specificity than protein-coding transcripts and systematic approaches to unravel their role have converged to indicate developmental functions [10–12]. In a number of cases, specific lncRNAs were reported as linked to human diseases, especially in cancer [13]. There are numerous reports testifying alterations of the expression levels of several lncRNAs in cancer [10, 13] and the involvement of lncRNAs as controllers of the availability of specific miRNAs [14]. In breast cancer, either aberrant expression or tumorigenic functions of a number of lncRNAs were reported [15, 16]. Analysis of lncRNAs expressed in breast cancer and their correlation with clinicopathological parameters are available from several studies [17–24] and possible roles in endocrine resistance suggested [1]. In addition, the noncoding response to estrogen stimulation *in vitro* has been extensively described using both RNA-Seq [16] and GRO-Seq analysis in breast cancer cell lines [25–27].

We report here that in hormone-deprived conditions ERα controls the expression of 133 lncRNAs that are highly specific for luminal breast cancer and that consequently can be used as biomarkers of this specific subtype. In addition, we found that the most abundant of these lncRNAs, DSCAM-AS1, is highly specific to ERα+ luminal cells and correlates with a specific stage of breast cancer. Moreover, DSCAM-AS1 deletion can extensively mimic the effect of deleting ERα in breast cancer cells.

## RESULTS

### ERα down-regulation in absence of hormones defines a set of differentially expressed lncRNAs

**Figure 1:**
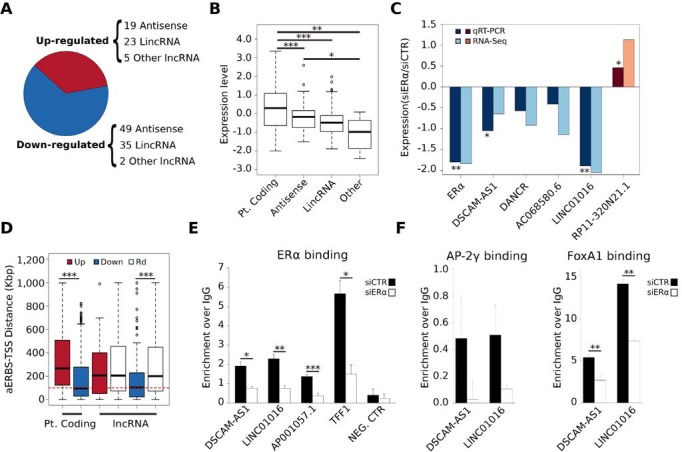
Features of Apo-ERα regulated lncRNAs (AER-lncRNAs) **A.** Fraction of down-regulated (blue) and up-regulated (red) lncRNAs in siERα- *vs* siCTR-transfected MCF-7 cells; in brackets details of biotypes are shown. “Other lncRNAs” includes few cases classified as “processed-transcript”, “sense intronic” and “sense overlapping” in the database. **B.** Box plot showing expression levels of protein coding, antisense, lincRNAs and “other” lncRNAs regulated by Apo-ERα, in terms of log10 RPKM (Reads Per Kilobase per Million mapped reads); ***p-value < 0.00001 and *p-value < 0.05. **C.** Quantitative evaluation by qRT-PCR of expression change of five AER-lncRNAs in siERα- *vs* siCTR-transfected MCF-7, (dark blue and dark red bars). Values are log2 FC (fold-change). **p-value < 0.01 and *p-value < 0.05. For comparison, corresponding data from RNA-Seq are shown in the plot as light blue and light red bars. **D.** Box plot reporting the distance of most proximal AERBS (Apo-ERα Binding Site) from the TSS of differentially expressed genes. In the case of protein coding genes, a direct comparison between down-regulated (blue) and up-regulated (red) RNAs is shown. In the case of lncRNAs, due to the smaller number, calculation is made separately for down- (blue) and up-regulated (red) genes with a random set (Rd) of lncRNAs of the same size (white boxes). The red dashed line indicates a distance of 100 Kb; ***p-value < 0.0001. **E.** ChIP-qPCR analysis of ERα binding to *DSCAM-AS1*, *LINC01016* and *AP001057.1* AERBS-containing 5′-flanking regions in hormone deprived MCF-7 cells. The *TFF1* promoter was used as a positive control, whereas the region upstream to *KCNQ1OT1* TSS, where no AERBS was identified, was selected as negative control (error bars are SD of four independent biological replicates); ***p-value < 0.001, **p-value < 0.01 and *p-value < 0.05. **F.** ChIP-qPCR analysis of AP-2γ and FoxA1 binding to *DSCAM-AS1* and *LINC01016* putative promoters upon ERα silencing in hormone deprived MCF-7 cells (error bars are SD of three independent biological replicates); **p-value < 0.01.

In our previous work, we carried out Apo-ERα chromatin binding analysis as well as poly(A+)-RNA-Seq analysis of MCF-7 cells cultured in hormone-deprived media and transfected with ERα-specific double-stranded interfering RNA (siRNA), or control siRNA [4]. Starting from this RNA-Seq data, by integrating Differentially Expressed (DE) genes defined by three algorithms (DESeq, EdgeR and RegionMiner), and filtering out protein-coding genes, short transcripts (length minor than 200 bp) and pseudogenes, we have compiled a list of 133 Apo-ERα-dependent lncRNAs (AER-lncRNAs) (Figure 1A, Supplemental Table 1A).

The most represented biotypes were “antisense” and “lincRNA” (Figure 1A). Both classes showed levels of expression, measured as RPKM (reads per Kilobase transcript per million reads), significantly lower than protein-coding genes, as expected (Wilcoxon Rank-Sum *p*-value < 0.00001) (Figure 1B). In total, 86 lncRNAs were down-regulated and 47 up-regulated in siERα transfected cells as compared to control. Validation relative to four down-regulated and one up-regulated lncRNAs using qRT-PCR in MCF-7 cells cultured in hormone-deprived medium and transfected with ERα-specific siRNA or control siRNA is shown in Figure 1C, demonstrating consistency to what observed in RNA-Seq data.

Taking advantage of our previously published map of AERBS (“unliganded” or Apo-ERα Binding Sites) [4], we next asked if AER-lncRNAs also had evidence of ERα binding within their genomic domain (within ± 100 Kb from gene TSS) and evaluated the distance of the closest AERBS from lncRNA gene TSS. This analysis showed that lncRNAs that are down-regulated by ERα depletion display closer AERBS than a random set of lncRNAs of equivalent size and biotype (Wilcoxon Rank-Sum *p*-value < 0.00001) (Figure 1D). This was expected assuming that Apo-ERα has prevalent trans-activating effect on linked promoters. The majority of AERBS were distant from TSS of regulated lncRNA genes, suggesting that they represent enhancers. To address this point further, we took advantage of published TFs and histone Post-Translational Modifications (PTMs) ChIP-Seq data obtained on MCF-7 cultured in hormone-deprived medium (i.e. treated with vehicle alone in experiments of 17β-estradiol-induction) (Supplemental Table 2), which corresponds to our basal condition. Mapping of this data clearly demonstrated that AERBS attributable to AER-lncRNAs show clear enhancer histone PTMs patterns (Supplemental Figure 1A). In addition, binding of FoxA1, AP-2γ, Forkhead box protein M1 (FoxM1) and histone acetyltransferase p300 in the ± 1 Kb region around AERBS illustrates not only their prevalent nature as enhancers, but also that they resemble closely the AERBS found within protein coding gene domains [4]. On the other side, histone PTMs at AER-lncRNA gene TSS clearly show promoter features, with high H3K4me3/H3K4me1 ratio (Supplemental Figure 1B).

ERα binding within the promoter region was observed in few cases, e.g. the *DSCAM-AS1* and *LINC01016* genes. We measured Apo-ERα binding to these regions by ChIP-qPCR, in comparison to the well-known estrogen-dependent *TFF1* gene and to the lncRNA *AP001057.1* gene, which has an AERBS close to TSS, albeit not promotorial (4.5 Kb upstream). In all cases, we could confirm ERα binding in cells cultured in hormone-deprived medium (Figure 1E). Concordant decrease of Apo-ERα binding and lncRNAs transcription is evident upon ERα down-regulation (cf. Figures 1E and 1C), and this fact correlated with the decreased binding of the two pioneer factors FoxA1 and AP-2γ, as described previously (Figure 1F) [4].

Altogether, these results demonstrate that ERα controls lncRNA transcription in MCF-7 cells cultured in hormone-deprived media, and this is achieved in a very similar manner to that previously observed in the case of protein coding genes.

### AER-lncRNAs dependence on ERα does not imply estrogen responsiveness

**Figure 2:**
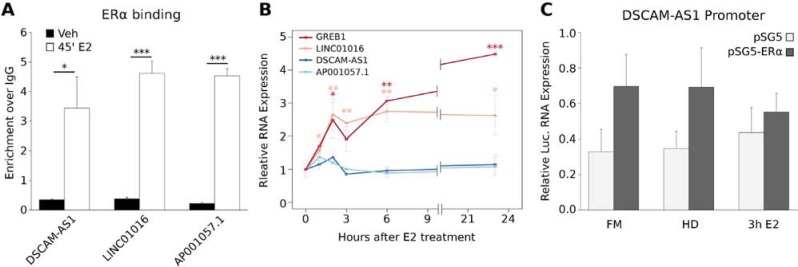
Estrogen-independent expression of AER-lncRNAs **A.** ChIP-qPCR analysis of ERα binding 45 minutes after E2 treatment to *DSCAM-AS1*, *LINC01016* and *AP001057.1* AERBS-containing 5′-flanking regions (error bars are SD of three independent biological replicates); ***p-value < 0.001 and *p-value < 0.05. **B.** qRT-PCR analysis of GREB1 (positive control, red), LINC01016 (orange), DSCAM-AS1 (dark-blue) and AP001057.1 (light-blue) expression in MCF-7 cells over a 24-hour time-course after E2 treatment (error bars are SD of three independent biological replicates); ***p-value < 0.001, **p-value < 0.01 and *p-value < 0.05. **C.** Luciferase reporter analysis of DSCAM-AS1 promoter in HEK 293T cells. Cells were transfected with 2Kb-DSCAMAS1p-Luc in combination with an empty pSG5 (light-grey bars) or ERα-expressing pSG5 vector (dark-grey bars), then grown in full medium (FM) or in hormone deprived medium minus (HD) or plus 10 nM E2 (3 h E2). Luciferase is expressed as firefly/renilla ratio and normalized to basal luciferase expression (error bars are SD of three independent biological replicates).

Next, we asked whether lncRNA genes regulated by Apo-ERα were also estrogen-responsive. For this comparison, we took in account only the transcripts that are directly responsive to 17β-estradiol (E2) in MCF-7 cells. We exploited three published GRO-Seq experiments [25–27] and found that only 31 out of 133 AER-lncRNAs responded transcriptionally to estrogen stimulation in these experiments (Supplemental Table 1B). Moreover, four of these 31 AER-lncRNAs were regulated by ERα deletion in a direction that was not coherent with the effect of E2, i.e. Apo-ERα sustains basal expression, while E2-bound ERα represses it. This observation suggested that the AER-lncRNA set described here is specifically responsive to ERα deletion, rather than representing a simple subset of the estrogen-responsive signature. Therefore, we decided to address this point further. We examined *DSCAM-AS1*, *LINC01016* and *AP001057.1,* which are robustly down-regulated by ERα siRNA (Figure 1C). These lncRNA genes display clear AERBS near their TSS or not far upstream (see above). By ChIP-qPCR analysis we observed a very significant increase in ERα binding to these three sites 45 minutes after E2 treatment (Figure 2A). On the contrary, time-course analysis of RNA expression by qRT-PCR after E2 treatment showed that, while LINC01016 increased 2.5-fold, in parallel with the well-known estrogen-regulated gene *GREB1*, neither *DSCAM-AS1* nor *AP001057.1* transcription responded to E2 over a period of 24 hours (Figure 2B). Especially in the case of *DSCAM-AS1*, which presents the AERBS very close to TSS, this may pose the question whether this site is the one responsible for the observed regulation after ERα silencing. To respond to this issue, we cloned a 2 Kb fragment of the *DSCAM-AS1* 5′-flanking sequence, containing the observed AERBS, in a luciferase reporter vector and examined its activity in HEK 293T cells co-transfected with an ERα-expressing vector. As shown in Figure 2C, ERα expression induced a significant, though not dramatic, increase in luciferase expression, whereas notably no further response was elicited by three-hour E2 treatment. Taken together, the above results allow defining *DSCAM-AS1* as an ERα-regulated, but estrogen-independent lncRNA gene.

We found further cases of incoherent response to ERα down-regulation or activation by E2 by comparing the effects of estradiol versus ICI 182,780 (“Fulvestrant”), which induces degradation of ERα protein (not shown). Taken together, this data suggests that AER-lncRNAs are a specific population of transcripts, which is in part distinguished from estrogen-regulated lncRNAs, representing a signature of unliganded ERα function in breast cancer cells.

### AER-lncRNAs define Luminal subtype of breast cancer cell lines and tumors

The Affymetrix Human Genome U133 Plus 2.0 microarray platform, used to measure the gene expression level of 1,037 cancer cell lines in the Cancer Cell Line Encyclopedia (CCLE) [28], contained probes for 38 AER-lncRNAs. Exploring this resource, we found that a number of AER-lncRNAs are expressed in cancer cell lines of non-breast origin. However, some of them, notably DSCAM-AS1, LINC01016, LINC00925, KRTAP5-AS1, were quite specific to breast cancer cell lines (Supplemental Figure 2A).

**Figure 3:**
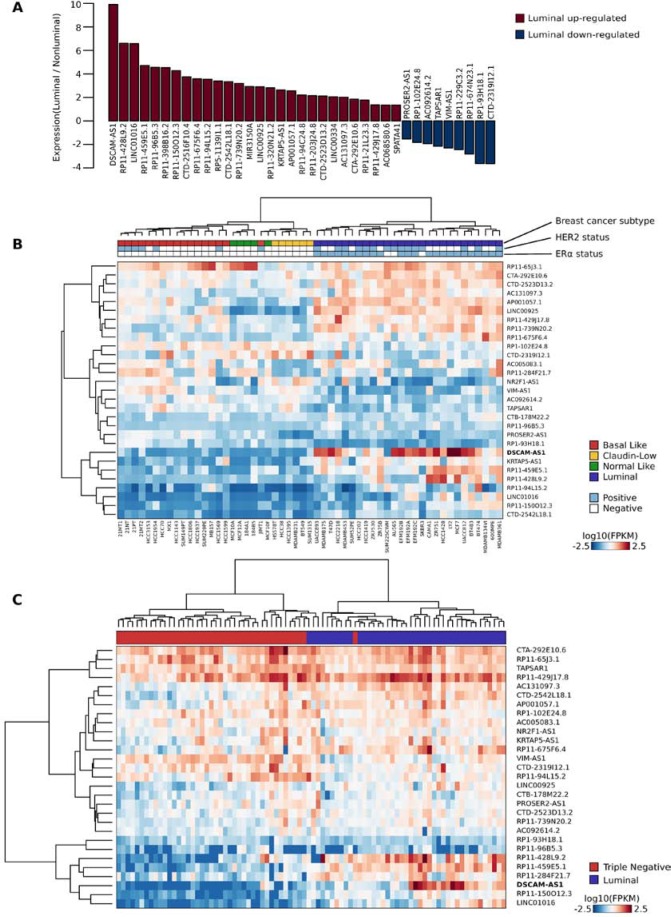
A 29-AER-lncRNAs signature defines luminal subtype of breast cancer cell lines and tumors **A.** Bar plot representing the differential expression of AER-lncRNAs in breast cancer cell lines of the luminal subtype as compared to cell lines of other subtypes (non-luminal). The most significant 37 lncRNAs are shown, plotting the relative prevalence as log2 fold change. Data from RNA-Seq analysis of 55 breast cancer cell lines were used [29]. **B.** Heat map illustrating expression values of the 29-AER-lncRNAs with highest classification “merit” (rows) in 55 breast cancer cell lines (columns) [29]. For each sample, the tumor subtype is color-coded below the dendrogram (basal-like = red, claudin-low = yellow, normal-like = green and luminal = blue), together with the Her2 status (positive = light blue; negative = white), and the ERα status (positive = light blue; negative = white). **C.** Heat map illustrating expression values of the 29-AER-lncRNAs signature (rows) in 84 breast tumor tissue samples (columns) from published dataset [31]. Tumor classification is limited to “Luminal” and “Triple negative” and is indicated below the dendrogram with blue and red boxes, respectively. Values are expressed as log10 FPKM (Frequency Per Kilobase per Million fragments) and color-coded as indicated.

More importantly, we asked whether AER-lncRNA expression is an ERα-dependent signature limited to the MCF-7 cell line or if it may represent a more general signature of luminal breast cancer. AER-lncRNA expression was evaluated using the data published on 55 breast cancer cell lines [29], classified as luminal, basal, claudin-low and normal-like by the authors. Thirty-seven AER-lncRNAs revealed a significant differential expression (at least *p*-value < 0.001) in luminal versus non-luminal cells, the majority of them being overexpressed in luminal cells (Figure 3A). DSCAM-AS1 was associated to the highest differential, i.e. log2 fold change equal to 9.91 (Figure 3A). Importantly, 22 out of 28 lncRNAs up-regulated in luminal cells and seven out of nine down-regulated had concordant changes upon ERα silencing in MCF-7 cells, i.e. luminal-overexpressed are down-regulated, and vice-versa.

Next, we sought to identify an AER-lncRNA signature suitable to classify correctly breast cancer subtypes. As a first step, we verified if AER-lncRNA expression was able to classify breast cancer cell lines. Using a multi-layered perceptron classifier [30], we predicted cell line subtypes with an accuracy of 96.36% (Supplemental Table 3). Then, we evaluated the relative contribution (also called *merit*) of each AER-lncRNA to the classification, using the leave-one-out approach (Supplemental Figure 2B). Figure 3B shows the performance of clustering breast cancer cell lines: the first 29 AER-lncRNAs showing the highest *merit* (> 15) were extremely effective. Note that the right branch is composed by “pure luminal” cancer subtypes, and also non-luminal cell lines were correctly clustered with only one incorrect call (one basal-like clustered as normal-like). The fact that the whole AER-lncRNA set is a general luminal signature was confirmed by removing the 29 “high-merit” AER-lncRNAs. The remaining AER-lncRNAs still classified correctly 80% of the cell lines (Supplemental Table 3). Next, we moved to validate this signature using an independent dataset of breast tumor biopsies. To this goal, we took advantage of data on 84 breast tumors from a published study [31], whose RNA-Seq data were downloaded and re-processed. This database contains only information concerning “Luminal” versus “Triple Negative” subtypes and we did not attempt re-classification. The 29-AER-lncRNA signature was very efficient in clustering tumor biopsies, with the left branch including pure “non-luminal” tumors and the right branch containing only one false call (Figure 3C). Principal Component Analysis (PCA) using this signature confirmed a clear separation of the samples (Supplemental Figure 2C). This 29-AER-lncRNA signature clearly outperformed the clustering ability of the whole AER-lncRNA set both on cell lines (cf. Figure 3B with Supplemental Figure 2D) and on tumor biopsies (cf. Figure 3C with Supplemental Figure 2E).

Altogether, these results demonstrate that AER-lncRNAs derived from MCF-7 cells represent a general luminal signature that can be used to discriminate breast tumor subtypes.

### DSCAM-AS1 is a major discriminant of the luminal subtype in breast cancer cell lines and tumors

**Figure 4:**
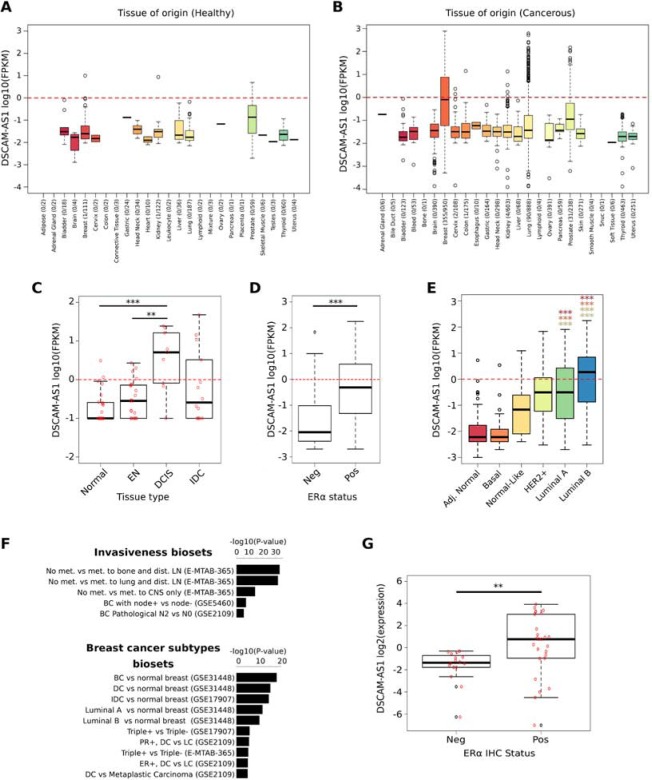
DSCAM-AS1 is a major discriminant of the luminal subtype in breast cancer cell lines and tumors **A-B.** Box plots showing DSCAM-AS1 expression in healthy (A) and neoplastic human tissues (B) extracted from the miTranscriptome RNA-Seq database [11]. For each tissue type, the number of samples where DSCAM-AS1 had a FPKM > 0 over the number of analyzed samples is reported in brackets. FPKM = Fragments Per Kilobase per Million mapped fragments. The red dotted line corresponding to log10 (FPKM) = 0 is the cut-off value conventionally considered for calling as positive a lncRNA expression. **C.** Box plot reporting DSCAM-AS1 expression in tissue samples normal, early neoplasia (EN), ductal carcinoma in situ (DCIS) and invasive ductal carcinoma (IDC) of the breast from a public dataset [33]; ***p-value < 0.0005 and **p-value < 0.005. **D-E.** Box plots showing DSCAM-AS1 expression in 839 breast tumor tissues, derived from the TCGA database [34]. In panel D tumor tissues are clustered as ERα-positive (Pos) and ERα-negative (Neg). In panel E the samples are classified as “adjacent normal tissue” or as tumor of the “basal”, “normal-like”, “HER2 positive”, “luminal A”, and “luminal B” subtype, accordingly to the PAM50 classifier. ***p-value < 0.00001. The *p*-values reported with color-coded stars refer to the comparison between luminal A and luminal B *versus* the other subtypes, as follows: Red = Adjacent normal; Orange = Basal; Ochre = Normal-Like; Light-green = HER2+. **F.** Bar plots reporting the differential expression of DSCAM-AS1 in the NextBio collection of breast cancer subgroups [35]. The significance of DSCAM-AS1 differential expression in each comparison is reported as -log10 (*p*-value). For each breast cancer bio-set, there is a brief description and the ID of the data (in brackets). Abbreviations are as follows: met = metastasis; dist. LN = distal Lymph Nodes; CNS = Central Nervous System; DC = ductal carcinoma; LC = lobular carcinoma; IDC = invasive ductal carcinoma; PR = Progesterone Receptor. **G.** Box plot showing the distribution of DSCAM-AS1 expression assessed by qRT-PCR in 42 RNA samples derived from 16 ERα-negative (Neg) and 26 ERα-positive (Pos) breast tumor biopsies (IHC = immunohistochemistry; Supplemental Table 4); red circles are individual values, black circles the outliers; **p-value = 0.0021.

When examining AER-lncRNAs individually, DSCAM-AS1 outstands for several features. First, it had the highest value of RPKM among differentially expressed lncRNAs in MCF-7 (RPKM = 394 in siCTR condition, Supplemental Table 1A); second, it is characterized by the widest differential range of gene expression between luminal and non-luminal cell lines (log2 fold change = 9.91) and, third, it showed significant correlation with ERα expression in both cell lines and tumor samples (see above). Finally, DSCAM-AS1 was already described in a cDNA library of MCF-7 cells subtracted with a benign cell line and designated as M41, and reported as expressed at higher levels in breast cancer than in normal tissue and benign lesions [32]. Therefore, we examined DSCAM-AS1 in deeper detail. First, we addressed the question whether DSCAM-AS1 is specific to cancer *versus* normal cells and, specifically, to breast cancer cells. Analysis of 6,249 RNA-Seq datasets extracted from the miTranscriptome database [11] demonstrated very low expression levels in normal tissues (Figure 4A), whereas elevated expression levels of DSCAM-AS1 were found essentially in breast cancer tissues, with few overexpressing cases derived from lung, prostate and kidney carcinoma (Figure 4B). This fact was essentially confirmed using the CCLE microarray resource described above (Supplemental Figure 3A). Second, we asked whether DSCAM-AS1 expression was specific to breast cancer progression, by examining a recently published RNA-Seq dataset [33]. This study examined gene expression in Formalin-Fixed Paraffin-Embedded (FFPE) tissue samples, comparing normal tissues, pre-neoplastic lesions, in situ carcinoma (DCIS) and invasive carcinoma (IDC) from breast cancer patients, using the 3SEQ procedure, which determines the reads in the RNA region preceding the poly(A) tail. This approach is valid in the case of *DSCAM-AS1*, since all four described DSCAM-AS1 transcripts have a common 3′ end (see below). As shown in Figure 4C, high levels of DSCAM-AS1 were observed only in overt carcinoma tissues, while it is almost undetectable in either the adjacent normal tissues or pre-neoplastic lesions. The difference between DCIS and IDC samples is not significant, albeit the box plot depicts a more regular distribution in DCIS than in IDC.

Next, we wanted to establish the relationship of DSCAM-AS1 expression to both ERα and breast tumor subtypes in a more robust way. We examined the miTranscriptome data of 839 breast cancer tissues from the TCGA database [34] and found that DSCAM-AS1 expression is significantly higher in ERα+ versus ERα- cases (*p*-value < 0.00001) (Figure 4D). Moreover, following the subtype classification of samples based on the PAM50 signature, it is possible to observe that Luminal A and -even more- Luminal B tumors express significantly higher levels of *DSCAM-AS1* compared to adjacent normal tissues and tumors of the Basal and Normal-like subtypes (Figure 4E). Instead, HER2+ tumors show DSCAM-AS1 levels comparable to Luminal A.

Looking further to confirm these findings, we analyzed the differential expression of DSCAM-AS1 in the subgroup of breast cancer cases, defined by different clinicopathological features that are collected in the NextBio database [35]. All studies giving *p*-values of differential expression < 0.00001 are plotted in Figure 4F and Supplemental Figure 3B. In addition to confirming the clear association of DSCAM-AS1 expression in cancer *vs* normal breast, in ERα+ *vs* ERα-, and in luminal *vs* triple-negative subtypes, we observed also higher DSCAM-AS1 expression in tumors that are not metastatic *vs* metastatic, although not all the metastatic sites were considered in these studies. Only marginal association to positive axillary lymph nodes was observed.

**Figure 5:**
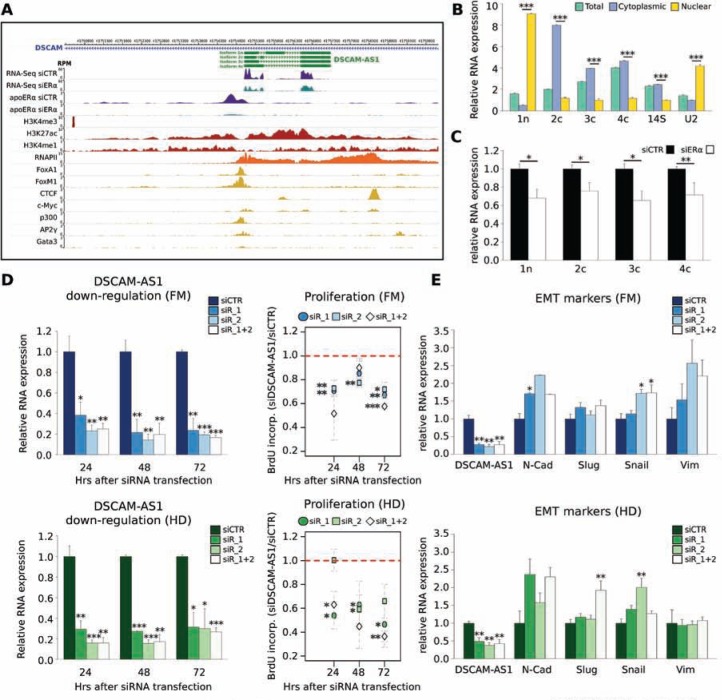

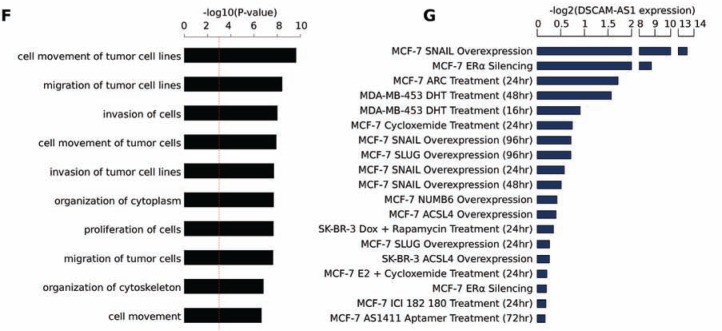
DSCAM-AS1 lncRNA is functional to the basal ERα activity **A.** Genome-browser view of the *DSCAM-AS1* locus. *DSCAM-AS1* (green; 1n = nuclear and 2c, 3c, 4c = cytoplasmic isoforms) is reported in association with Apo-ERα ChIP-Seq and RNA-Seq reads enrichment in siCTR- (violet) or siERα- (teal-blue) transfected MCF-7 cells; and with ChIP-Seq profiles of histone modifications (red), RNA-Pol II (orange) and seven TFs (yellow). Histograms are in Reads Per Million (RPM). **B.** DSCAM-AS1 isoforms expression by qRT-PCR in total, cytoplasmic and nuclear RNA fractions from MCF-7 cells. Isoforms are numbered in the same order as they are shown in the browser in (A). “n” and “c” indicate the nuclear or cytoplasmatic localization reported in the literature. 14S ribosomal RNA and U2 small nuclear RNA were used as fractionation controls. Error bars are SD of three independent biological replicates; ***p-value < 0.001. **C.** Effect of Apo-ERα down-regulation on DSCAM-AS1 isoforms by qRT-PCR. Error bars are SD of 3 independent biological replicates; **p-value < 0.01 and *p-value < 0.05. **D. Left**, DSCAM-AS1 expression measured by qRT-PCR in MCF-7 cells grown in full medium (FM, **upper** panels) or in hormone-deprived medium (HD, **lower** panels) and transfected with control siRNA (siCTR) or with two different siRNAs targeting DSCAM-AS1, alone (siR_1 and siR_2) or in combination (siR_1+2) (error bars are SD of 3 independent biological replicates); ***p-value < 0.001, **p-value < 0.01 and *p-value < 0.05. **Right**, MCF-7 cell proliferation, measured as BrdU incorporation (error bars are SD of 3 independent biological replicates); ***p-value < 0.001, **p-value < 0.01 and *p-value < 0.05. **E.** N-cadherin (N-Cadh), Slug, Snail and Vimentin (Vim) expression by qRT-PCR in MCF-7 cells grown in full medium (FM, **upper** panel) or in hormone-deprived medium (HD, **lower** panel) and transfected as in D (error bars are SD of 5 independent biological replicates); **p-value < 0.01 and *p-value < 0.05. **F.** Bar plot illustrating the functional analysis of genes whose expression is positively or negatively correlated with DSCAM-AS1 expression in RNA-Seq data of 55 breast cancer cell lines (29) (Supplemental Table 5A). The significance of Biological Functions enrichment (Supplemental Table 5B), as evaluated by Ingenuity Pathway Analysis, is expressed as -log10 (*p*-value). **G.** Bar plot reporting DSCAM-AS1 expression level in the 19 experiments from the GEO database reporting the effects of various treatments on MCF-7 cells, where DSCAM-AS1 was significantly down-regulated (*p*-value < 0.05). DSCAM-AS1 down-regulation is reported as -log2 FC (fold change).

This *in silico* data prompted us to further examine DSCAM-AS1 expression in a fresh series of breast cancer biopsies using qRT-PCR. To this goal, we designed PCR primer pairs spanning the constitutive last exon (see below and Figure 5A) and we ran qRT-PCR assays on 42 breast tumor samples (Supplemental Table 4). Quantitative data confirmed that DSCAM-AS1 expression is significantly higher in ERα+ breast cancer (Figure 4G), but no further correlation with other clinicopathological data was obtained. In conclusion, the AER-lncRNA DSCAM-AS1 is a stage-specific marker of luminal breast cancer.

### Preliminary characterization of DSCAM-AS1 lncRNA

This data also prompted us to examine further traits of the *DSCAM-AS1* gene and functions. *DSCAM-AS1* has four transcripts of length less than 2 Kb annotated in Gencode and Ensembl, differing mainly for the presence or absence of a central exon. The transcription unit is entirely contained, in antisense, in the third, 324 Kb intron of the *DSCAM* gene on chromosome 21 (Figure 5A). By mapping data from several published studies (Supplemental Table 2) performed in MCF-7 cells in hormone-deprived medium, we obtained the functional representation of this region shown in Figure 5A. Apo-ERα presents a bimodal peak close to DSCAM-AS1 TSS. The most proximal peak summit is a composite site bound by FoxM1, FoxA1 and AP-2γ transcription factors and, as in many other ERBSs, these TFs cooperate (see also Figure 1F).

We analyzed the expression of *DSCAM-AS1* isoforms in MCF-7 subcellular fractions by qRT-PCR using specific primers and confirmed that the isoform containing the central exon is mostly nuclear, while the other isoforms are cytoplasmic (Figure 5B). All four isoforms were similarly down-regulated after ERα silencing (Figure 5C), in keeping with a transcriptional effect. To further confirm the association of DSCAM-AS1 expression with ERα, we analyzed ERα and DSCAM-AS1 RNAs in a panel of breast cancer cells by qRT-PCR, confirming that DSCAM-AS1 is confined to cells expressing ERα, with the exception of cells with HER2 amplification (SK-BR-3), as already noticed in previous *in silico* analysis (Supplemental Figure 3C). Interestingly, we observed complete down-regulation of DSCAM-AS1 in the T-47D-sfRon cell line as compared to parental cells. T-47D-sfRon were obtained by transducing T-47D cells with the oncogenic short form of the *RON* gene that caused complete loss of ERα expression and almost complete EMT [5, 36]. This further supports the conclusion that DSCAM-AS1 expression depends, at least in part, on the presence of ERα. Consequently, since experimental down-regulation of ERα leads, as a first evident consequence, to growth arrest in MCF-7 cells, we asked whether DSCAM-AS1 was essential for the growth-sustaining action of unliganded ERα. We down-regulated DSCAM-AS1 expression using two different siRNAs, targeting the last exon common to the four transcripts. As shown in Figure 5D (left panels), siR_1 and siR_2 alone or in combination effectively down-regulated DSCAM-AS1 RNA over a period of 72 hours after transfection. Bromo-deoxyuridine incorporation was assayed at three-time points (24–48–72 hrs) after siRNA transfection and in all cases a significant reduction was observed when compared to cells transfected with control siRNA (Figure 5D, right panels). This reduction tended to increase with the time, in particular when hormone-deprived medium (HD) was used (Figure 5D, lower right panel) and reflected most likely both a decreased fraction of cells in S-phase and an increased cell death, as measured by FACS analysis (Supplemental Table 7). Notably, no significant change in ERα mRNA was noticed after DSCAM-AS1 silencing (Supplemental Figure 3D). DSCAM-AS1 siRNA also up-regulated mesenchymal markers N-Cadherin, Slug, Snail and Vimentin (Figure 5E), and induced partial morphological changes similar to what is observed after ERα down-regulation (not shown).

To get further insight on the possible function of DSCAM-AS1, we went back to the RNA-Seq data from 55 breast cancer cell lines [29] used before, and ran a correlation analysis, in order to find which genes are most frequently co-expressed. By adjusting the correlation to an absolute value of *r* ≥ 0.7 and by applying a threshold of *p*-value = 0.001, 205 genes demonstrated significant co-expression with DSCAM-AS1 (Supplemental Table 5A). We analyzed this co-expressed gene set using Ingenuity Pathway Analysis [37] and we observed that the most enriched terms were related to cell motility, adhesion and cancer cells invasion, further emphasizing a possible role of DSCAM-AS1 in the control of epithelial integrity (Figure 5F and Supplemental Table 5B).

To further corroborate this hypothesis, we analyzed 107 microarray experiments of diverse experimental treatments of breast cancer cell lines present in Gene Expression Omnibus (Supplemental Table 2). From this analysis, we identified 19 treatments leading to significant DSCAM-AS1 down-regulation (at least *p*-value < 0.05). As illustrated in Figure 5G, the treatments leading to the most significant reduction of DSCAM-AS1 were those leading to EMT, i.e. Snail overexpression (GSE58252) and ERα silencing (GSE27473).

In conclusion, the function of DSCAM-AS1 lncRNA appears related to cell survival and proliferation and to EMT, confirming a strict relationship of DSCAM-AS1 with ERα in luminal breast cancer cells.

## DISCUSSION

In this work, we describe a novel set of ERα-dependent long noncoding RNAs that are mostly luminal-specific and appear extremely promising in defining subclasses of breast tumors. In addition, we present preliminary studies on DSCAM-AS1 lncRNA that is the most closely associated to ERα expression in breast tumors and has possible functions in cell development and EMT.

Several groups have addressed lncRNA profiles in breast cancer either by examining tumor biopsies or in experimental settings. The novelty of our data relies on the experimental model system they were obtained from, i.e. transient silencing of ERα expression using MCF-7 cells cultured in hormone-deprived media [4]. In other words, we have not addressed the transcriptional response of breast cancer cells to estrogen or other ligands, as many other published studies reported [16, 26, 27, 38], but to the basal activity of ERα, which represents the leading protein of Luminal A and B subtypes. Since the most common endocrine treatment today is Aromatase Inhibitors, which deplete the organism of estrogenic hormones, we believe that unliganded ERα action is extremely important to understand luminal breast tumor growth and progression. Albeit breast tumor cells often overexpress ERα, ERα binding to chromatin in absence of hormones has been reported in mouse uterus [39] an in other contexts [2, 3].

AER-lncRNAs do not appear simply as a miniaturized E2-responsive signature, since a significant fraction of AER-lncRNAs does not respond to estrogen, as we ascertained by data integration as well as experimentally. It should be also noted that our ERα-dependent lncRNA set does not contain most of the lncRNAs that were previously studied in breast cancer, such as HOTAIR, CCAT2, UCA1, MALAT1, BCAR4, EGOT, SPRY4-IT1 and others [15, 17–24]. They show only limited overlap with other Luminal signatures [40, 41]. Based on our analysis using datasets from both breast cancer cell line collections [29] and breast tumors [31], this set contains a very powerful signature to distinguish not only luminal subtype, but also basal-like cancers.

AER-lncRNAs are regulated transcriptionally by ERα in a way that appears very like the same as for protein-coding gene, i.e. by remote enhancer elements. Indeed, we did not find evidence of enhancer-specific chromatin marks at AER-lncRNA TSS. In this respect, our results are different from those recently published by Sun and coworkers, who integrated GRO-Seq and RNA-Seq data to describe estrogen-induced lncRNAs in MCF-7 cells [16]. GRO-Seq experiments are much more sensitive to nuclear, short-lived transcript than steady-state poly(A)+ RNA-Seq and will detect most of enhancer RNAs (eRNAs) induced by estrogen. Thus, our lncRNA set does not contain eRNAs and is more enriched of relatively stable lncRNAs, which is possibly an advantage for their use as cancer biomarkers.

In search of possible functions of AER-lncRNAs, we have started a preliminary characterization of DSCAM-AS1. It is quite astonishing that DSCAM-AS1, originally described by Liu and coworkers [32], has escaped the attention of many studies that have addressed lncRNA expression in breast cancer, especially due to the fact that, among all transcripts (protein coding and non-coding) DSCAM-AS1 classifies as the eighth most abundant RNA in luminal breast cancer cell lines (the first considering only lncRNA genes) and that it is neatly associated to ER-positivity. Perhaps the confounding effect of HER2+ tumors that also express DSCAM-AS1 has led to disregarding. DSCAM-AS1 expression was recently described as differentially expressed also in lung adenocarcinoma [42].

The proximal AERBS that overlaps the DSCAM-AS1 promoter (Supplemental Figure 4) may be involved in the regulation of DSCAM-AS1 transcription in ER+ breast cancer cells, as also suggested by our reporter assays (Figure 2C). This region contains a single ERE that is unusually close to the TSS (−26), corresponding to the proximal summit of the biphasic AERBS (Figure 5A and Supplemental Figure 4). Whether Apo-ERα directly regulates DSCAM-AS1 transcription by binding to this region awaits more direct proofs, such as measuring the effect of deleting the ERE or the use of DNA-binding defective ERα mutants. Furthermore, we do not know the mechanism leading to overexpression of DSCAM-AS1 in HER2+ tumors and cell lines. In ER+/HER2+ cases, constitutive ERα activation through phosphorylation has been observed [3]. In absence of ERα, other transduction pathways may result in the transcriptional activation of DSCAM-AS1, perhaps targeting the other TFs that are present near DSCAM-AS1 TSS, as FoxM1, FoxA1 or AP-2γ. This point deserves further studies.

*DSCAM-AS1* is antisense intronic within the *DSCAM* gene (Down Syndrome Cell Adhesion Molecule). Low levels of the coding DSCAM transcript are detectable in breast cancer cells, where it appears induced by estrogen [43]. Albeit the hypothesis of antisense/sense transcriptional control could be attractive, especially considering the fact that *DSCAM* encodes a protein important in cell adhesion, no function of *DSCAM* in mammary tissues has been described to date. Deletion of ERα also decreases slightly DSCAM mRNA with slow kinetics, concordant with DSCAM-AS1. However, silencing of DSCAM-AS1 does not affect DSCAM mRNA level (Supplemental Figure 3E). This data suggests that DSCAM-AS1 has its own function, independent of its host gene, in ERα+ breast cancer cells. Conversely, silencing of DSCAM-AS1 mimics some features of ERα silencing [4, 6] since it reduces cell growth, increases cell death and induces EMT markers without influencing ERα expression. This is suggestive of a function of DSCAM-AS1 downstream ERα that is probably limited to breast carcinoma development. However, further studies are needed to fully understanding DSCAM-AS1 function. The lack of conservation of DSCAM-AS1 below Primates would render it difficult to assess its role, if any, in mammary gland development.

Analysis of possible functions was made by examining the correlation of gene expression between AER-lncRNA and protein-coding genes in published studies. This is an inductive way of considering function association and, of course, it should be validated by more direct approaches, such as lncRNA deletion or ectopic expression. Nevertheless, it is intriguing that DSCAM-AS1 expression is inversely correlated with genes with function in cell motility. This may explain why DSCAM-AS1, albeit clearly associated to neoplastic growth, is very high in ductal carcinoma in situ, whereas its expression may be reduced when cells escape cell-to-cell contacts, undergo EMT and acquire invasiveness. This is also proven by the fact that down-regulation of DSCAM-AS1 in MCF-7 cells led to increased expression of mesenchymal markers. Clearly, further work is needed to fully understand the functions of DSCAM-AS1, as well as other AER-lncRNAs, and to establish whether DSCAM-AS1 may mediate some of the functions of unliganded ERα.

Overall, ERα+ breast tumors (Luminal A and B) respond well to endocrine treatments at both the adjuvant and advanced settings. Nonetheless, one-fourth of these cases present either primary resistance or acquired resistance during treatment. This makes development of new markers and targets an important challenge [8]. The set of ERα-dependent, luminal-specific lncRNAs presented here will be further studied as possible markers and targets. One of these, the carcinoma-specific DSCAM-AS1, is particularly interesting since it is functional to ERα action, but does not respond to estrogen, thus representing an ideal marker of ERα function in absence of hormones.

## MATERIALS AND METHODS

### Cells culture

MCF-7, HEK 293T, MDA-MB-231 and SK-BR-3 cells were routinely grown in DMEM (Life Technologies, 31053–028); T-47D, T-47D-sfRON and ZR-75–1 cells were grown in RPMI (Life Technologies, 31870–025) and HTERT-HME1 cells were grown in DMEM/F12 (Life Technologies, 21041–025). All media were supplemented with 10% heat-inactivated FBS (Biochrom, S0115–1), 2 mM L-glutamine (Life Technologies, 25030–024), 50 U/ml penicillin and 50 μg/ml streptomycin (Life Technologies, 15140–122) and only in DMEM/F12 was added 1x mammary epithelial growth supplement (containing bovine pituitary extract, bovine insulin, hydrocortisone and recombinant human epidermal growth factor). Hormone-deprived medium (HD) was obtained from phenol red-free DMEM (Life Technologies, 31053–028) supplemented with 5% charcoal-dextran-treated serum. 17β-estradiol (E2) (Sigma, E2758–1G) was added at a final concentration of 10 nM. Batches of human cell lines were purchased from ATCC. Cell culture was at 37°C with 5% CO_2_.

### Small interfering RNA (siRNA)

MCF-7 cells were grown both in full medium (FM) and in hormone-deprived (HD) medium before being transfected with siRNAs (20 nM final concentration) using Lipofectamine2000 (Life Technologies, 11668–019), according to the manufacture's protocol. Stealth RNAi^TM^ siRNAs from Life Technologies were used to target ERα mRNA [4]; custom-designed Stealth siRNAs from Life Technologies were used to target DSCAM-AS1 lncRNA (siR_1: 5′-ACUCAUCCAUGUACCCAUUUCUUAA-3′ and siR_2: 5′-CCUCCUCCAACUGCCAUUUAUUUAU-3′); stealth RNAi™ siRNA Negative Control Med GC was used as a control (siCTR; Life Technologies, 12935–300). Unless otherwise specified, experiments were performed 48 hours after siRNA transfection.

### RNA isolation and quantitative Real-time PCR (qRT-PCR)

RNA was isolated from MCF-7 cells using the Trizol™ reagent (Life Technologies, 15596–026). Nuclear and cytoplasmic RNA fractions were obtained from MCF-7 cell pellets by lysis in 10 mM TRIS pH = 7.8, 140 mM NaCl, 1.5 mM MgCl_2_, 10 mM EDTA, 0.5% MP40 and 0.3U RNase inhibitor (Life Technologies, AM2694) for 5 min on ice and centrifugation at 3,000 x g for 3 min to obtain a cytosolic fraction and nuclear pellet, followed by Trizol™ extraction. Frozen breast cancer tissues (previously collected and stored at − 80°C) were directly homogenized in Trizol™ to extract total RNA. All total RNA samples were subjected to DNase treatment to remove contaminating genomic DNA (DNA-free^@^ DNA removal kit, Life Technologies, AM1907). First strand cDNA synthesis was performed with a M-MLV reverse transcriptase Kit (Life Technologies, AM2044-AM5722G-AM8228G. qRT-PCR analysis was performed using the SYBR-green method (iTaq UniverSYBR Green, Biorad, 1725124). Real-time PCR primers for human 18S (QT00199367), ERα (QT00044492), GREB1 (QT00080262), N-cadherin (QT00063196), SLUG (QT00044128) and SNAIL (QT00010010) RNAs were purchased from Qiagen (QuantiTect^@^ Primer Assay). Custom expression-primer pairs are reported in Supplemental Table 6.

### Chromatin immunoprecipitation assay (ChIP)

MCF-7 cells were grown for 2 days in HD medium before siRNA transfection or 17β-estradiol (E2) treatment. ChIP experiments were performed as described in [4]. qRT-PCR was carried out on ChIP-enriched DNA using SYBR Green Master Mix. ChIP enrichment was normalized on input samples (1% of total chromatin used per IP) and expressed as enrichment of specific binding over the control unspecific IgG binding. Antibodies against human ERα (Santa Cruz Biotechnology; sc534X, sc7207X), AP-2γ (Santa Cruz Biotechnology; sc-8977X), FoxA1 (Abcam; ab5089) and IgG (Abcam, ab46540) were used in this assay. Custom ChIP-primer pairs are reported in Supplemental Table 6.

### DSCAM-AS1 promoter cloning and promoter-activity assay

A BAC carrying a 25 Kb region of the human chromosome 21 (Life Technologies; clone ID: 3214D4; Chr21:41739181–41764048 genomic region) was used as DNA template for the *DSCAM-AS1* promoter amplification by high-fidelity PCR amplification (HiFi-Taq^@^ polymerase kit, Life Technologies, 11304–011). We amplified a 2 Kb sequence including approximately 170 bp sequence downstream to *DSCAM-AS1* TSS and 1,830 bp sequence upstream, by using the following primers:

- 2 Kb promoter Fwd: 5′-CCGCTCGAGCCTTTATAGAGATATGGAAAGGGGA-3′
- 2 Kb promoter Rev: 5′-CCCAAGCTTGTTCCAGCATTTCTCCTGC-3′

The amplified sequences were then purified and directionally cloned into pGL3-Basic-Luc (firefly) reporter vector (pGL3 Basic, Promega, E1751). Competent bacterial cells were used to amplify the obtained 2Kb-DSCAMAS1p-Luc vector. HEK 293T cells were transfected with the basal pGL3-Basic-Luc vector or the 2Kb-DSCAMAS1p-Luc vector and with pGL4-renilla vector (pGL4.73 [hRluc/SV40] Vector, Promega), as internal control of transfection efficiency, in combination with pSG5 empty or pSG5-hERα expressing vector (pHEGO vector, [44]). To assay for DSCAM-AS1 promoter activity, firefly luciferase mRNA production was evaluated by qRT-PCR, as firefly/renilla luciferase mRNA expression ratio, in FM, HD or 10 nM E2 treatment culture conditions, and was then normalized on the firefly/renilla luciferase mRNA expression ratio from pGL3 basic vector. Custom expression-primer pairs are reported in Supplemental Table 6.

### Cell proliferation assay and FACS analysis

Cell proliferation rate was assayed by measuring BrdU incorporation in a 2-hours pulse by the use of an ELISA format, according to Cell Proliferation Kit instructions (GE Healthcare Life sciences, RPN250).

FACS analysis was performed 48 hours after siRNAs transfection. Growth media and cells were harvested and fixed with 70% cold ethanol. Sample pellets were treated with 100 μg/ml RNase A (Qiagen, 19101) for 15 minutes at RT and then were incubated with 50 μg/ml Propidium Iodide (PI) for 2 hours at 4°C, protected from light. The DNA content of the cells, labeled with PI, was analyzed using a Cyan ADP flow cytometer (Beckman Coulter, Brea, CA, USA).

### RNA-Seq data analysis and identification of Apo-ERα regulated lncRNAs

AER-lncRNAs list was defined by analyzing our previously published RNA-Seq experiment performed in MCF-7 cells that were grown in HD medium and transfected with control (siCTR) or targeting-ERα (siERα) siRNAs (GSE535353). For RNA-Seq analysis, the strategy used in [4] was repeated using Gencode v19 as transcriptome reference and hg19 assembly (GRCh37) as genome reference. The final set of Differentially Expressed (DE) lncRNAs was defined by considering a transcript length threshold of 200 bp and by excluding pseudogenes and ambiguous annotations reported as discordant entries (i.e. both protein coding and noncoding gene) between Ensembl (version 75) and RefSeq (version 64) (e.g. SEMA3B, SSPO, CYP4F8, LEPREL2, TRAV39). DESeq-normalized read counts were converted to Reads per Kilobase of Exon per Million Reads Mapped (RPKM) using the longest-isoform length (in Kb) and the million number of reads counted by HTSeq (63.63 and 61.31 million in siCTR and siERα condition, respectively).

### Computation of distances between DE lncRNAs and Apo-ERBSs

Distances between the TSS of lncRNAs and the center of Apo-ERα Binding Sites (AERBS; [4]) were computed by using an ad-hoc Perl script and by establishing 1,000 Kb as maximum distance-threshold. One thousand random sets of lncRNAs with the same composition in biotypes were used as control sample. The differences in distances distribution were statistically evaluated using the Wilcoxon Rank-Sum test.

### Computational analysis of public ChIP-Seq datasets

ChIP-Seq signals of RNAPII, H3K4me3, H3K4me1, H3K27ac and p300, were analyzed in a genomic window of ± 1 Kb around the TSS of DE lncRNAs and protein coding genes.

The AERBSs mapped within 100 Kb from TSSs of both lncRNAs and protein coding genes were analyzed for ChIP-Seq signal of AP-2γ, c-Myc, CTCF, FoxM1, FoxA1 and Gata3. From each study raw sequencing data were aligned with Bowtie v2.1.0 [45] in default settings; ChIP-Seq reads were counted with Seqminer [46] by considering 50 bp bins and 200 bp reads extension. Read counts were converted in Count Per Million (CPM) using the number of reads in each experiment. Only experiments performed in vehicle-treated MCF-7 were considered. See Supplementary Table 2 for the list of the dataset used.

### Computational analysis of public gene-expression datasets

#### GRO-Seq datasets

Raw data of vehicle or E2-treated MCF-7 for 10, 25, 40 and 60 minutes (GSE43835, GSE41324, GSE45822) were aligned with Bowtie v2.1.0 and aligned reads were counted with HTSeq [47]. Differential expression between vehicle and E2-treatment conditions was performed using DESeq v1.2.0 [48]. Genes associated with a *p*-value < 0.05 in at least one time-point were considered as differentially expressed. If multiple replicates in the same time-point were available the fold changes were averaged and the *p*-values combined using *metaRNASeq* R package [49].

#### CCLE

Normalized data from Cancer Cell Line Encyclopedia (CCLE) project [28] were downloaded from the project website (http://www.broadinstitute.org/ccle). The gene-identifier associated to each probe was updated to Ensembl 75 annotations and the expression levels of multiple probes were averaged if associated to the same gene. The expression values of each probe across all cell lines were converted to Z-Score by subtracting the mean and dividing by standard deviation.

#### RNA-Seq datasets

Public raw data of 55 breast cancer cell lines (GSE48213) and 84 breast cancer tissues (GSE58135) were aligned with Tophat v2.0.0 [50] and the reads were counted with HTSeq. Table of counts were normalized using DESeq and then converted to FPKM values. Molecular and clinical information about cell lines and tissues analyzed were obtained from information provided by authors. Considering the 55 cell lines from GSE48213, a differential expression between luminal and non-luminal subtypes was performed using DESeq v1.2.0. Unsupervised hierarchical clustering was performed using *heatmap.2* R function with *Ward.D2* method while PCA analysis using *prcomp* R function.

#### 3SEQ datasets

Data of 72 breast cancer tissues at different disease stages (GSE47462) were aligned with Bowtie v2.1.0 and reads counted with HTSeq. Molecular and clinical details were obtained from information provided by authors.

#### miTranscriptome database

Analysis of DSCAM-AS1 expression in 6,249 RNA-Seq experiments on tissue samples from miTranscriptome [11] was performed retrieving the lncRNA FPKM expression from project website (mitranscriptome.org). An average FPKM was computed among expression levels computed for the four DSCAM-AS1 isoforms.

#### Microarray datasets

Analysis of DSCAM-AS1 expression in published microarray experiments of breast cancer specimens was performed using NextBio web tool [35]. Only bio-sets associated with *p*-value < 0.00001 were considered. Microarray experiments performed in breast cancer cell lines using Affymetrix Human Genome U133 Plus 2.0 platform were analyzed with GEO2R. Only those experiments with at least two biological replicates were analyzed.

#### Unsupervised lncRNA selection by Weka

To identify a list of lncRNAs discriminating breast cancer subtypes, a machine-learning classification approach using Weka 3.6.12 [30] was applied. The four subtypes of breast cancer cell lines (luminal, basal, claudin-low and normal-like) were used as class label to be identified based on the expression of AER-lncRNA genes. Classification was performed with a *MultilayerPerceptor* classifier with ten folds cross-validation. The contribution of each lncRNA on the classification results was evaluated using Weka *ChiSquareAttributeEval* function with ten folds cross-validation. This method is based on the computation of a Chi-squared statistic for each attribute of the input (lncRNA) with respect to the class labels (i.e. breast cancer subtype). The larger the Chi-squared, the more relevant is the feature with respect to the class. The Chi-squared is used to compute the average *merit* of each lncRNA.

#### DSCAM-AS1 guilt-by-association analysis

To identify a list of DSCAM-AS1 correlated and anti-correlated genes, data from RNA-Seq experiments of 55 breast cancer cell lines (GSE48213) were considered to compute a pairwise Pearson correlation between expression of DSCAM-AS1 and all Gencode v19 genes. Only genes associated to an absolute *r* >= 0.7 and correlation *p*-value < 0.001 were retained. These genes were subjected to QIAGEN's Ingenuity Pathway Analysis (IPA) [37].

#### Transcription Factor Binding Sites (TFBSs) analysis

TFBSs analysis on the DSCAM-AS1-promoter AERB was performed by using MATCH™ algorithm in default settings [51].

### Statistical analysis

For proliferation, ChIP-qPCR and gene-expression (qRT-PCR) data the standard deviation (SD) was computed among independent experiments and the *t*-test was used to compute the *p*-values. DSCAM-AS1 expression in breast cancer tissue samples was evaluated in two different runs (*N* = 22; *N* = 20). In order to merge the data, values were computed as ΔCt from qRT-PCR (18S Ct/DSCAM-AS1 Ct). Values of the two series were then ranked, log-transformed (in order to deal with non-normality of the data) and regressed over the values of series 1. A bootstrap approach with 200 iterations was applied in order to minimize the estimated mean square error. Then, the estimates of the regression model were used to adjust the log-transformed values of the Series 2 according to the location and scale parameters of log-transformed values of the Series 1. Finally, they were back-transformed on the original scale. Wilcoxon Rank-Sum test was used to assess differences between sample 1 and sample 2, Shapiro-Wilk test was applied to assess data normality.

## SUPPLEMENTARY FIGURES AND TABLES




