# Supplementary material for: Luminal long non-coding RNAs regulated by estrogen receptor alpha in a ligand-independent manner show functional roles in breast cancer
Source: Oncotarget. 2015 Nov 28;7(3):3201–16. doi: 10.18632/oncotarget.6420 (PMC4823100; doi:10.18632/oncotarget.6420)
Supplement: Supplementary file 1 [file oncotarget-07-3201-s001.pdf]

SUPPLEMENTARY FIGURES AND TABLES

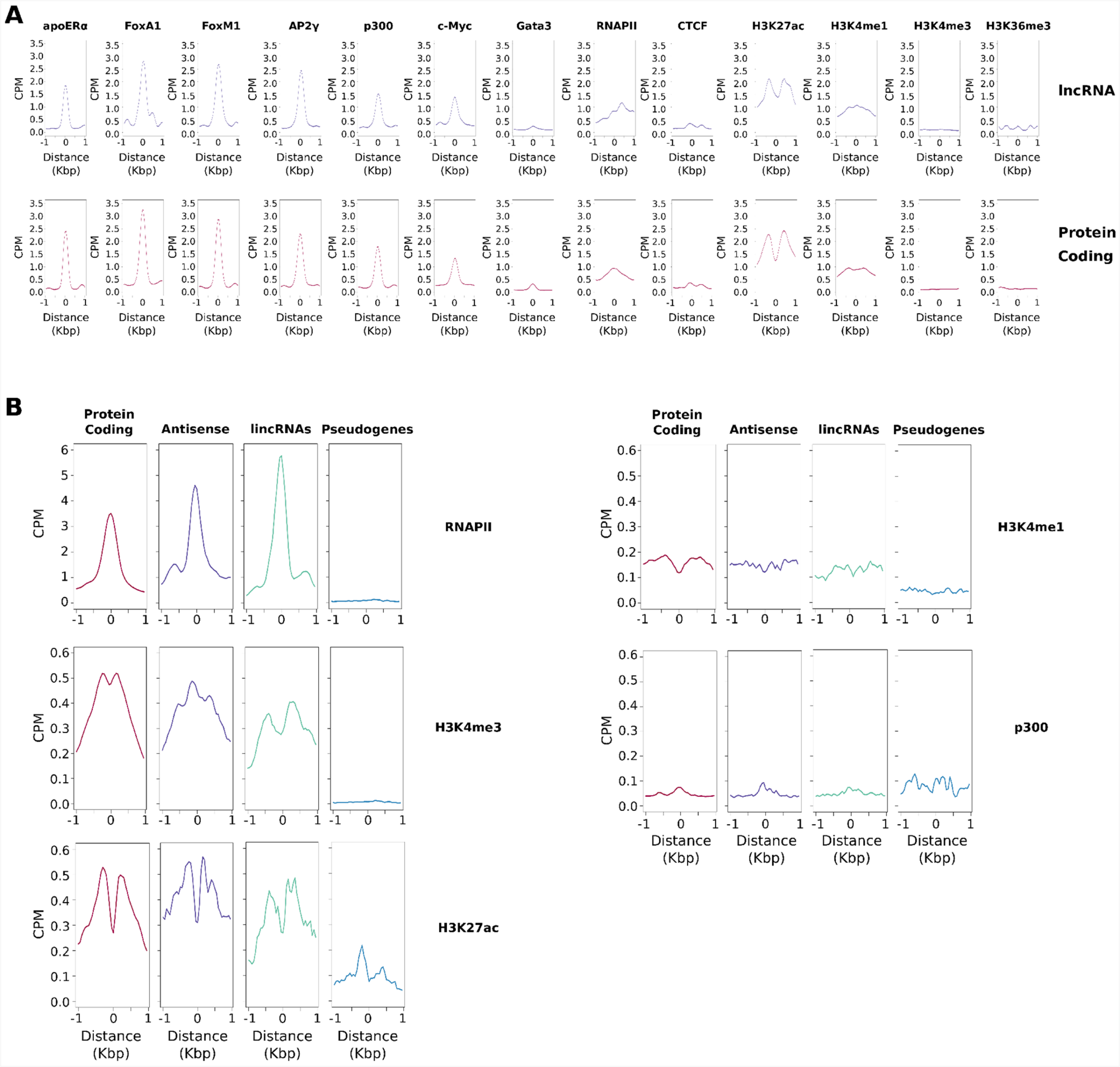

Supplementary Figure S1: Chromatin and TF profiles at AERBSs and AER-lncRNA TSSs. A. Intensity plots showing the profile of different Transcription Factors (TF), histone modifications and RNA-Pol II binding at lncRNA- and protein coding-associated AERBS in MCF-7 cells (Supplemental Table 2). CPM = counts per million. **B.** Intensity plot of ChIP-Seq average signal of RNA-Pol II (RNAPII), p300 and histone modifications across protein-coding and lncRNA Transcriptional Start Sites (± 1Kb from the TSS) in MCF-7 cells (Data are taken from References in Supplemental Table 2).

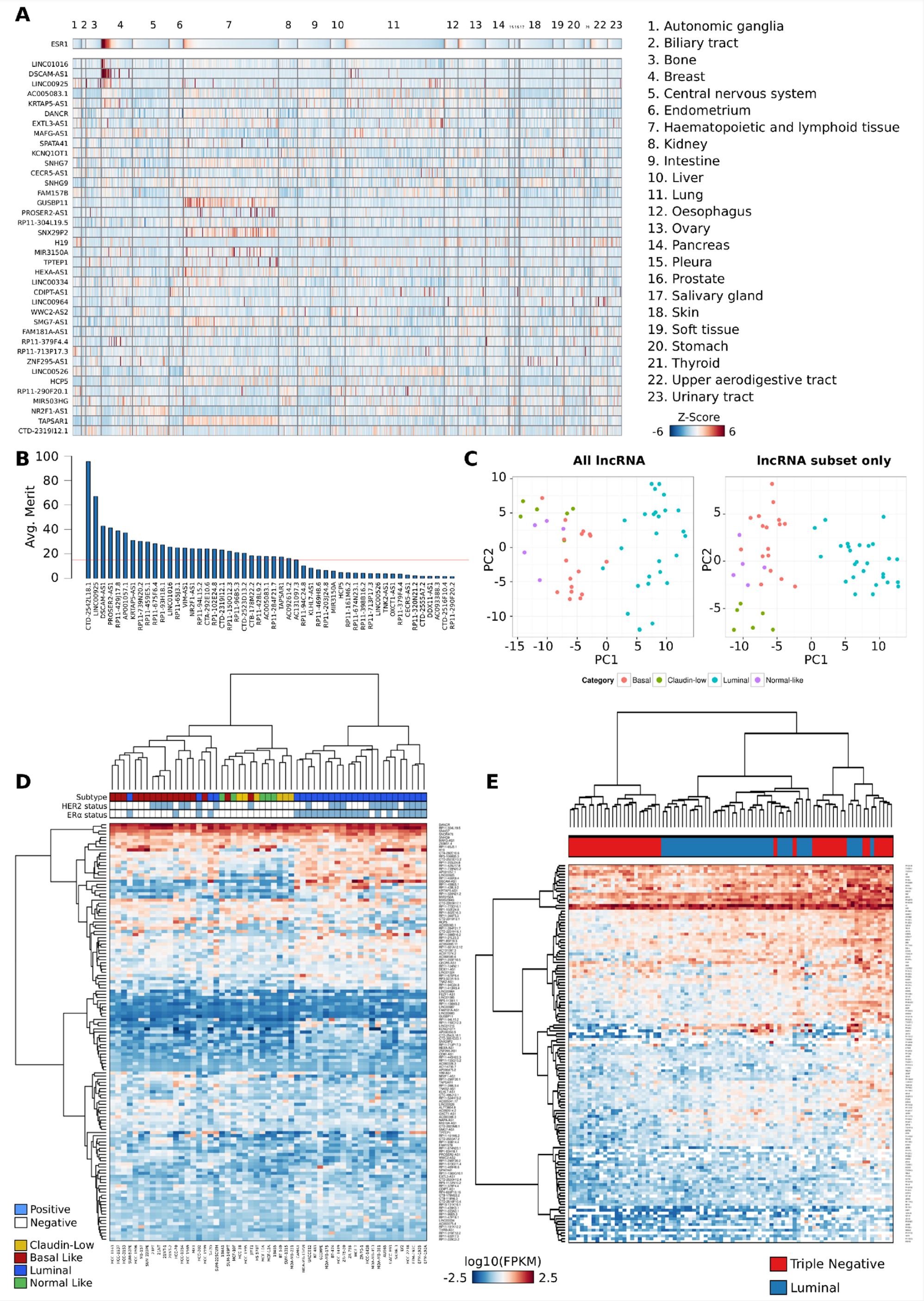

Supplementary Figure S2: AER-lncRNAs define luminal subtype of breast cancer cell lines and tumors. A. Heat map showing the value of 38 AER-lncRNAs expression (rows) in 1037 cancer cell lines from 23 different tissues (columns), reported as z-scores. Within each tissue, columns are ranked according to ERα expression (reported in the first row of the heat map). **B.** Bar plot reporting the Weka computed classification *merit* associated to each AER-lncRNA. Only lncRNAs associated to a *merit* > 0 are reported, while those associated to *merit* > 15 (red line) were used as a 29-AER-lncRNA signature in Figure 3 (main text). **C.** PCA plot reporting the clustering of 55 breast cancer cell lines by using the expression values of all AER-lncRNAs (**left**) or 29-AER-lncRNA signature (**right**). **D.** Heat map illustrating the expression values of all AER-lncRNAs (rows) in 55 breast cancer cell line (columns). For each sample, the tumor subtype is color-coded below the dendrogram (basal-like = red; claudin-low = yellow; normal-like = green, luminal = blue), together with the HER-2 status (positive = light blue; negative = white), and the ERα status (positive = light blue; negative = white). **E.** Heat map illustrating the expression values of all AER-lncRNAs (rows) in 84 Breast Cancer tissue samples (columns). Tumor classification is limited in this series to "luminal" and "triple negative", as indicated under the dendrogram by blue and red boxes, respectively.

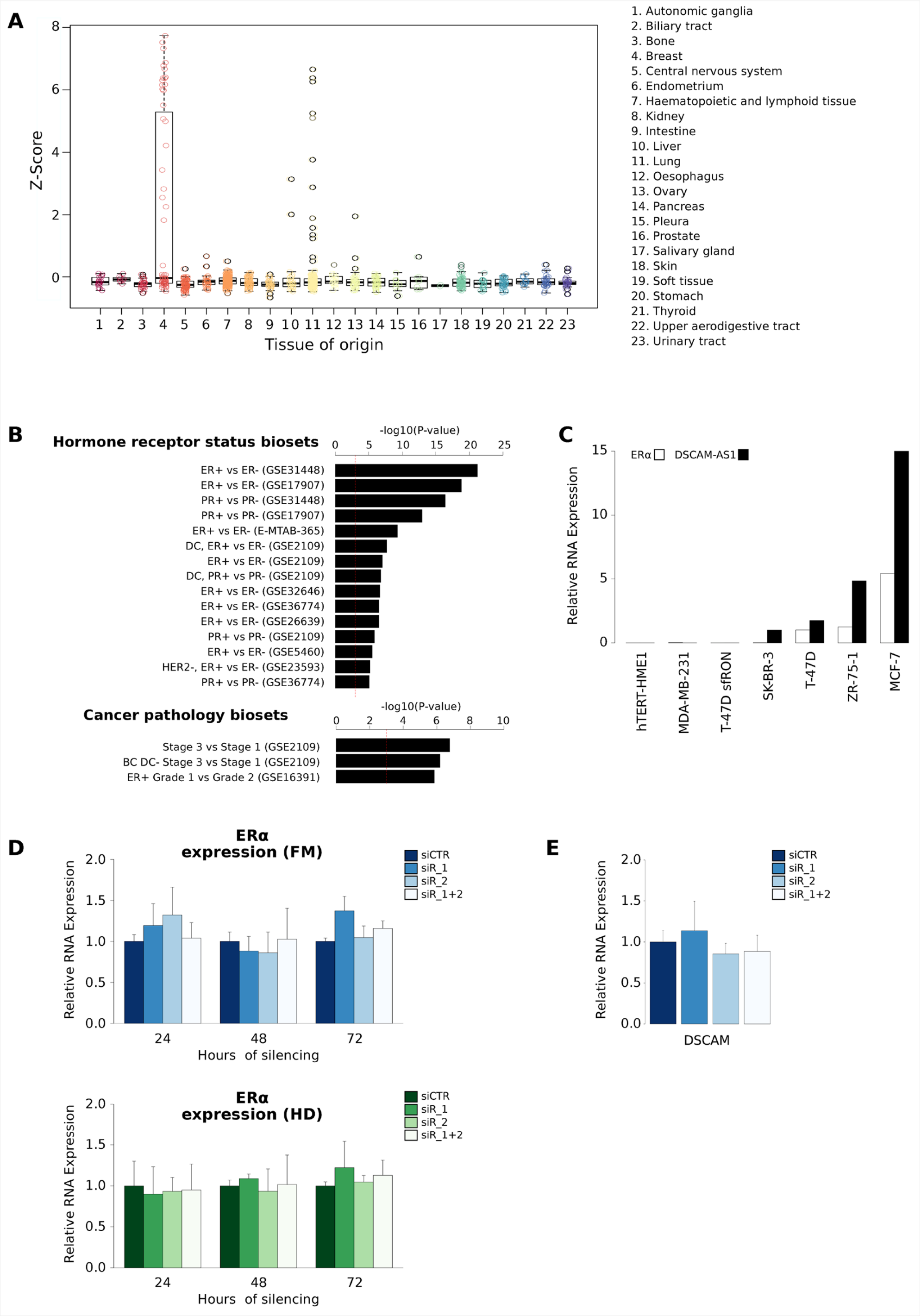

Supplementary Figure S3: DSCAM-AS1 as a luminal, ERα-regulated lncRNA. A. Box plot showing DSCAM-AS1 expression in 1037 cancer cell lines from 23 different tissues. Tissue types are associated to a color, and their DSCAM-AS1 expression levels reported as z-scores. Colored circles are individual values, and black circles are outliers. **B.** Bar plots reporting the differential expression of DSCAM-AS1 in the NextBio collection of breast cancer subgroups. The significance of DSCAM-AS1 differential expression in each comparison is reported as -log10 (*p*-value). For each breast cancer bio-set, there is a brief description and the ID of the dataset (in brackets). PR = Progesterone Receptor; BC DC = ductal carcinoma. **C.** qRT-PCR analysis of ERα and DSCAM-AS1 expression in 7 different breast cancer cell lines. **D.** ERα expression measured by qRT-PCR in MCF-7 cells grown in full medium (FM, **upper panels**) or in hormone-deprived medium (HD, **lower panels**) and transfected with control siRNA (siCTR) or with two different siRNAs targeting DSCAM-AS1, alone (siR_1 and siR_2) or in combination (siR_1+2) (error bars are SD of 3 independent biological replicates). **E.** Analysis of the protein-coding DSCAM hosting gene mRNA by qRT-PCR in MCF-7 cells grown in full medium and transfected with control siRNA (siCTR) or with two different siRNAs targeting DSCAM-AS1, alone (siR_1 and siR_2) or in combination (siR_1+2) (error bars are SD of 5 independent biological replicates).

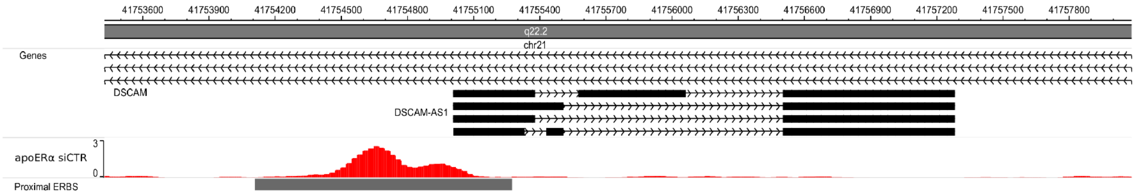

>GRCh37 – chr21:41754111:41755277 – DSCAM-AS1 Upstream sequence

```
1    - TTAAATTAAAGATTCCTGCTTTAGCAAGTCCCCTAGTTCTTATTCCTGGTAGGGGGACTC
61   - TTCACCTGTTCAGGGCTCCTCACCAAGACCTAGCAGCCGCCTTGGTCCTCATCTCCTTTG
121  - CCTCATCCTGACATTCAGGCCAATTCTATGTCCTATCAGTTCTATTTTCAAAGTATATCT
181  - TGTATCATTTCCTTGGACATGACACATTTTTTCCCACCTCAAACACATGCTTTGAGACCA
241  - GCAAGTCTATAAGGCCATAGGCTTGGTTGCATTTATAATGGGAATAGCAGAGAAAGCAGT
301  - TTATTTTTTTAAAACTGCAACATCATTTGAAGGTCTTGGATTACATTTGTTTCCCAATGA
361  - CACGTGATGATGTCAAAGGAAACTATAATGAGTTTACTGAGCTATCCATACAAATGTACA
421  - TGCAAATATGTATACTTACAAGAACACGCAAGGGTAGAAACCATGTCAGATGCGGTCCCT
481  - CGTGTCACTCCCACTCCACTGCCTTTTGGTTTCAAATAGTCTGACCCCATGAGAATCTTT
541  - GAGACTGTCTTTCATGTCACAGAGCCTTGCACTGTGAGAGTAACTCAATTCCCTAAATGA
601  - CTTCCCCAGATCTAGCTTATCACAAACTCCATTGCCTTGCAATAACCTTTGTAACAAAAT
661  - GGTATTAATAAAATCATGTAGCAACTGCCCATCATATGCAGACCTAGTAATGGAATCCAA
721  - GTTACCTAAAAGGCTAATTCCTAATGAGAAGGAAAGGAAACATACCATAAAGTAAAAGTG
781  - CAAACAATTGCAAAACAGAGGCAAACAGTGTCAGTTGGATATACGTAAGCGCTGATGTAA
841  - AGAGAGATTGGAAATGATATATCTGGCTGGGCAGGTCACTCATGCCTGTAATCCCAGCAC
901  - TTTGGGAGGCTGAGGCAGGCAGATCACCTAAGGCCAGGAATTCGACACCAGCCTGGCCAA
961  - CGTGGCAAAACCCGTCTCTACTAAAAATACAAAAATTAGCCGGGCGTGGTGGTGTGCGCC
1021 - TGGAATCCCAGCTACCCAGGAGGCTGAGGCAGGAGAAATGCTGGAACCCGGGAGGCAGAG
1081 - GCTGCAGTGAGCTGAGATCATGCCACTACTGCACTCCAGCCTGGGTGACACAGCAAGACT
1041 - CCCTCTAAAAAAAGAAAAAAAGAAAAG
```

Matrix	Position (strand)	Core match	Matrix sequence	Factor
V$PAX6_01	353 (-)	1.000	tcccaatgaca**CGTGA**tgatg	Pax-6
V$MYCMAX_02	359 (-)	1.000	tgac**ACGTG**atg	c-Myc/Max
V$USF_Q6	360 (-)	1.000	gaca**CGTGA**t	USF
V$OCT1_Q6	417 (+)	1.000	gtacat**GCAAA**tatg	Oct-1
V$OCT1_06	418 (-)	0.964	taca**TGCAA**atatg	Oct-1
V$CHOP_01	636 (+)	1.000	cct**TGCAA**taacc	CHOP-C/EBPalpha
V$EVI1_04	846 (+)	0.907	agattggaa**ATGAT**a	Evi-1
V$ER_Q6	872 (-)	1.000	gca**GGTCA**ctcatgcctgt	ER

ID	Position (strand)
**DSCAM-AS1 TSS**	900 (+)
**1° Peak summit**	553 (+)
**2° Peak summit**	853 (+)

Supplementary Figure S4: Structure of the putative DSCAM-AS1 promoter and analysis of Transcription Factor motifs. In the **upper** panel, the same genomic browser view as in Figure 5A is zoomed in, to show in detail the sequence identified by unliganded ERα ChIP-Seq analysis. The sequence of this AERBS (± 200 additional nucleotides) is shown **below**, with annotation of the TF binding motifs identified by MATCH™ [51]. A single ERE is evidenced, 26 bp upstream the major TSS; this position corresponds to the center of the second, minor peak in ChIP-Seq data.

Supplementary Table 1A: AER-lncRNAs. This Table reports the Ensemble ID, gene symbol and biotype of AER-lncRNAs, together with their expression values in siCTR- and siERα-transfected MCF-7 cells, the calculated average log2 (Fold Change), the combined *p*-value associated with FC and, in the last column, the distance of the main TSS of each lncRNA from the closest AERBS. AS = antisense; SI = sense intronic; SO = sense overlapping; PT = processed transcript.

Supplementary Table 1B: GRO-Seq analysis. This Table reports the values of Log2 (Fold Change), with the associated *p*-value, derived from a published GRO-Seq analyses 10, 25, 40 and 60 min after E2-treatment in MCF-7 cells (data from GSE43835, GSE41324, GSE45822 datasets).

Supplementary Table 2: Analyzed public dataset references. The Table reports the experimental identifier (e.g. GEO number ID), the experimental conditions and the assay type for each dataset analyzed.

Supplementary Table 3: Weka confusion matrices

**All AER-lncRNAs**				
Correctly Classified Instances: 53 (96.3636%)				
**Confusion matrix**				
**Normal-like**	**Basal**	**Luminal**	**Claudin-low**	**< — classified as**
5	0	0	0	**Normal-like**
0	16	0	1	**Basal**
0	1	26	0	**Luminal**
0	0	0	6	**Claudin-low**
**AER-lncRNAs without 29-AER-lncRNA signature**				
Correctly Classified Instances: 44 (80%)				
**Confusion matrix**				
**Normal-like**	**Basal**	**Luminal**	**Claudin-low**	**< — classified as**
2	1	1	1	**Normal-like**
0	15	1	1	**Basal**
1	1	25	0	**Luminal**
0	4	0	2	**Claudin-low**
**29-AER-lncRNA signature only**				
Correctly Classified Instances: 53 (96.3636%)				
**Confusion matrix**				
**Normal-like**	**Basal**	**Luminal**	**Claudin-low**	**< — classified as**
5	0	0	0	**Normal-like**
0	16	1	0	**Basal**
0	1	26	0	**Luminal**
0	0	0	6	**Claudin-low**

The number of correctly classified breast cancer cell lines is reported for each subset of AER-lncRNAs considered.

Supplementary Table 4: Breast tumor data

Histology number	Diagnosis	Grade	pT	pN	IHC ER	IHC PR	IHC HER2 score	FISH HER2
BC_1	IDC	3	3	1a	-	0	0	NA
BC_2	IDC	3	1c	1a	-	0	1	NA
BC_3	IDC	3	2	2a	-	0	0	NA
BC_4	IDC	3	2(m)	0 sn (i-)*	-	0	0	NA
BC_5	IDC	3	2	0	-	0	3	NA
BC_6	IDC	3	2m	0 (i-) sn*	-	0	1	NA
BC_7	IDC	3	2	1a	-	0	0	NA
BC_8	IDC	3	2	0 (i-) sn*	-	0	0	NA
BC_9	Metaplastic Carcinoma	3	2	1a*	-	0	0	NA
BC_10	IDC	3	1c	1a*	-	0	0	NA
BC_11	ILC	3	2	1a	-	0	0	NA
BC_12	IDC	2	2	3a	+	38	2	no amplification
BC_13	IDC	2	1c	1a	+	55	1	NA
BC_14	IDC	3	2	0 sn*	+	95	1	NA
BC_15	IDC	2	2	1a	+	20	0	NA
BC_16	IDC	2	1c	1a*	+	35	1	NA
BC_17	ILC	2	3	3a	+	70	3	NA
BC_18	IDC	3	2	1a*	+	25	0	NA
BC_19	IDC	3	2	2a	+	20	0	NA
BC_20	IDC	2	1c	0 (i-) sn*	+	65	0	NA
BC_21	IDC	3	2	1a*	+	10	0	NA
BC_22	IDC	3	1c	1c	+	70	1	NA
BC_23	DCIS	NA	is	NA	+	0	3	NA
BC_24	IDC	2	1c	0	-	0	3	NA
BC_25	IDC	3	4b	0	-	0	3	NA
BC_26	IDC	3	2	1a	-	0	1	NA
BC_27	IDC	3	2	0 (i+) sn*	-	1	1	NA
BC_28	IDC	3	2	0 sn)(i+)*	+	30	3	NA
BC_29	IDC	2	1c	1mic (sn) +	+	55	2	no amplification
BC_30	IDC	3	3(m)	3a	+	10	3	NA
BC_31	IDC	3	2	1a*	+	60	2	no amplification
BC_32	IDC/ILC	1	2	1a*	+	90	2	no amplification
BC_33	IDC	3	1c	1a*	+	60	3	NA
BC_34	IDC	2	2	1a	+	80	3	NA

(*Continued*)

Histology number	Diagnosis	Grade	pT	pN	IHC ER	IHC PR	IHC HER2 score	FISH HER2
BC_35	ILC	2	2	0mi*	+	98	1	NA
BC_36	IDC	2	1c	0(sn)*	+	75	1	NA
BC_37	IDC	2	1c	2a	+	50	2	no amplification
BC_38	IDC	2	1c	0 (i-) sn	+	90	3	NA
BC_39	IDC	3	2	0 (sn) (i-)*	+	5	3	NA
BC_40	IDC	2	2	0 (sn) (i-)*	+	70	3	NA
BC_41	IDC	2	1c	0 (sn) (i-)*	+	75	2	no amplification
BC_42	IDC	2	1c	0	+	25	3	NA

Histology, grade, pT and pN values, immunohistochemistry (IHC) of ERα, PR and HER2, score FISH number of HER2 are reported for the 42 tumor biopsies RNA analyzed by qRT-PCR (Figure 4G).

Supplementary Table 5A: Correlation analysis. List of DSCAM-AS1 correlated and anti-correlated genes from the 55 breast cancer cell lines dataset (Ref 29 maintext). Correlation values and *p*-values are reported for each gene. Pt.cod = protein coding; PSG = pseudogene; AS = antisense; SI = sense intronic; SO = sense overlapping; PT = processed transcript.

Supplementary Table 5B: IPA enriched functional annotations of DSCAM-AS1 correlated and anti-correlated genes. Genes are reported for each enriched annotation.

Supplementary Table 6: Custom Primers sequences

Custom EXPRESSION PRIMERS		Custom ChIP PRIMERS	
DSCAM-AS1	Fwd: 5′-GATCCTTGTTTGGTCTCACTCC-3′		Rev: 5′-AATCTCACGCAGGCAGTTCT-3′
	Rev: 5′-ATGCCTATGTGGGTGATTGG-3′	RENILLA luciferase	Fwd: 5′-AACACCGAGTTCGTGAAGGT-3′
DSCAM-AS1 1n nuclear	Fwd: 5′-TTCTCAGCACGTTTTTGCAG-3′		Rev: 5′-CATTTCATCTGGAGCGTCCT-3′
	Rev: 5′-CCGATCCGTCGTCCATCTC-3′	14S	Fwd: 5′-TTCTCAGCACGTTTTTGCAG-3′
DSCAM-AS1 2c cytoplasmic	Fwd: 5′-CCACTGATGGGAAAGCCACA-3′		Rev: 5′-CCGATCCGTCGTCCATCTC-3′
	Rev: 5′-GTGGAGGCACCTAAGTCAGT-3′	Vimentin	Fwd: 5′-ACCAGCTAACCAACGACAAAG-3′
DSCAM-AS1 3c cytoplasmic	Fwd: 5′-GTATGCAGCTGATAAGACGCT-3′		Rev: 5′-GCATCTCCTCCTGCAATTTT-3′
	Rev: 5′-TGAGATGGGGTTTCGCTCTT-3′	**Custom ChIP PRIMERS**	
DSCAM-AS1 4c cytoplasmic	Fwd: 5′-TTCTCAGCACGTTTTTGCAG-3′	DSCAM-AS1	Fwd: 5′-CCACTCCACTGCCTTTTGGT-3′
	Rev: 5′-CCGATCCGTCGTCCATCTC-3′		Rev: 5′-TGCAAGGCTCTGTGACATGAA-3′
LINC01016	Fwd: 5′-CGCAGAAGACAGACCGGTAG-3′	LINC01016	Fwd: 5′-GACTGCACTGGAGAGTGAGG-3′
	Rev: 5′-GGGCCAAACTGCACTTCTT-3′		Rev: 5′-TTCGAAGCAGCCTGTGAAGT-3′
DANCR	Fwd: 5′-CGCCTCTCTGGTTTGTGC-3′	AP001057.1	Fwd: 5′-CTAAGAGATGGGTGGCTGGAG-3′
	Rev: 5′-CCGATCCGTCGTCCATCTC-3′		Rev: 5′-GAGAAGCACGCACATCCTGC-3′
AC068580.6	Fwd: 5′-AGGTTCCAGAATGGGGAAGA-3′	TFF1	Fwd: 5′-CACCCCGTGAGCCACTGT-3′
	Rev: 5′-CCCGAGGTGCTCAAGAACT-3′		Rev: 5′-CTGCAGAAGTGATTCATAGTGAGAGAT-3′
RP11.320N21.1	Fwd: 5′-TTAGGACACCAGGCAGAAAGTT-3′	KCNQ1OT1	Fwd: 5′-CCCAGGGAGTTCATGTGTCT-3′
	Rev: 5′-AATGCCCAAGGCTGTCAT-3′	(negative control)	Rev: 5′-TGACAAAAACTTCCCAGCTAGA-3′
LINC01016	Fwd: 5’-CGCAGAAGACAGACCGGTAG-3’		
	Rev: 5’-GGGCCAAACTGCACTTCTT-3’		
DANCR	Fwd: 5’-CGCCTCTCTGGTTTGTGC-3’		
	Rev: 5’-CCGATCCGTCGTCCATCTC-3’		
AC068580.6	Fwd: 5’-AGGTTCCAGAATGGGGAAGA-3’		
	Rev: 5’-CCCGAGGTGCTCAAGAACT-3’		
RP11.320N21.1	Fwd: 5’-TTAGGACACCAGGCAGAAAGTT-3’		
	Rev: 5’-AATGCCCAAGGCTGTCAT-3’		
FIREFLY luciferase	Fwd: 5’-CTGGATCTACTGGTCTGCCTAAA-3’		
	Rev: 5’-AATCTCACGCAGGCAGTTCT-3’		
RENILLA luciferase	Fwd: 5’-AACACCGAGTTCGTGAAGGT-3’		
	Rev: 5’-CATTTCATCTGGAGCGTCCT-3’		
14S	Fwd: 5’-TTCTCAGCACGTTTTTGCAG-3’		
	Rev: 5’-CCGATCCGTCGTCCATCTC-3’		
Vimentin	Fwd: 5’-ACCAGCTAACCAACGACAAAG-3’		
	Rev: 5’-GCATCTCCTCCTGCAATTTT-3’		

Expression and ChIP custom primer pairs are listed as forward (Fwd) and reverse (Rev) sequences.

Supplementary Table 7: FACS analysis of MCF7 Cell Cycle upon DSCAM-AS1 silencing

Full Medium	% of apoptotic cells	% of cells in G0/G1 phase	% of cells in S phase	% of cells in G2/M phase
**siCTR**	22,39	51,91667	9,133333	17,35667
**siR_1**	28,05333	49,59333	8,08	15,02667
**siR_2**	28,52333	48,14333	8,83	15,38
**siR_1+2**	28,27667	50,42	8,556667	13,64
**Hormone-Depleted Medium**				
**siCTR**	29,52	50,27	8,016667	13,01667
**siR_1**	38,69333	42,64	7,49	12,14333
**siR_2**	41,48333	40,86667	6,683333	11,67333
**siR_1+2**	35,86333	41,27667	5,7	11,08

The table reports the percentage of cells in different cell cycle phases or in the apoptotic fraction as defined by FACS analysis. The result for cell grown in full or hormone deprived medium and transfected with control siRNA (siCTR) or with two different siRNAs targeting DSCAM-AS1, alone (siR_1 and siR_2) or in combination (siR_1+2) are reported.
