# Supplementary material for: Luminal long non-coding RNAs regulated by estrogen receptor alpha in a ligand-independent manner show functional roles in breast cancer
Source: Oncotarget. 2015 Nov 28;7(3):3201–16. doi: 10.18632/oncotarget.6420 (PMC4823100; doi:10.18632/oncotarget.6420)
Supplement: Supplementary file 2 [file oncotarget-07-3201-s002.docx]

**Supplemental Table 1A:** **AER-lncRNAs.** This Table reports the Ensemble ID, gene symbol and biotype of AER-lncRNAs, together with their expression values in siCTR- and siERα-transfected MCF-7 cells, the calculated average log2 (Fold Change), the combined p-value associated with FC and, in the last column, the distance of the main TSS of each lncRNA from the closest AERBS. AS = antisense; SI = sense intronic; SO = sense overlapping; PT = processed transcript.

| **Ensembl ID** | **Symbol** | **Biotype** | **RPKM siCTR** | **RPKM siERα** | **AVG log2FC** | **Combined P-value** | **AERBS dist (bp)** |
| --- | --- | --- | --- | --- | --- | --- | --- |
| ENSG00000100181 | TPTEP1 | lincRNA | 0.202 | 0.094 | -1.11 | 5.87E-04 | >1000000 |
| ENSG00000130600 | H19 | lincRNA | 6.824 | 2.007 | -1.82 | 0.00E+00 | 167832 |
| ENSG00000132832 | RP11-445H22.3 | AS | 0.122 | 0.023 | -3.06 | 8.95E-06 | 2894 |
| ENSG00000182586 | LINC00334 | lincRNA | 0.106 | 0.722 | 2.87 | 5.24E-07 | 93060 |
| ENSG00000185837 | CECR5-AS1 | AS | 0.356 | 0.983 | 1.42 | 7.08E-04 | >1000000 |
| ENSG00000189419 | SPATA41 | lincRNA | 0.507 | 0.182 | -1.69 | 3.71E-07 | 167142 |
| ENSG00000198106 | SNX29P2 | lincRNA | 0.034 | 0.008 | -1.33 | 4.25E-03 | 353474 |
| ENSG00000203999 | RP11-290F20.1 | lincRNA | 0.419 | 0.194 | -1.11 | 3.77E-05 | 7861 |
| ENSG00000204241 | RP11-713P17.3 | lincRNA | 0.227 | 0.067 | -1.97 | 2.75E-04 | 607990 |
| ENSG00000204261 | TAPSAR1 | lincRNA | 0.062 | 0.542 | 2.49 | 0.00E+00 | 277057 |
| ENSG00000206337 | HCP5 | SO | 0.354 | 1.908 | 2.39 | 0.00E+00 | 109484 |
| ENSG00000214725 | CDIPT-AS1 | AS | 0.373 | 0.087 | -1.80 | 3.47E-04 | 97869 |
| ENSG00000214733 | RP11-429J17.8 | AS | 1.793 | 0.772 | -1.40 | 9.00E-04 | 771491 |
| ENSG00000223502 | RP11-96B5.3 | AS | 1.563 | 0.389 | -2.19 | 2.31E-06 | >1000000 |
| ENSG00000223669 | RP11-93B14.4 | AS | 0.615 | 0.133 | -2.42 | 3.56E-06 | 91027 |
| ENSG00000223714 | RP5-1172N10.2 | lincRNA | 0.788 | 0.215 | -2.05 | 1.86E-06 | 698111 |
| ENSG00000223749 | MIR503HG | lincRNA | 1.546 | 0.603 | -1.64 | 6.01E-06 | 111959 |
| ENSG00000223808 | RP11-428L9.2 | lincRNA | 14.993 | 32.313 | 1.03 | 0.00E+00 | 64495 |
| ENSG00000224614 | TNK2-AS1 | AS | 0.658 | 0.148 | -1.14 | 0.00E+00 | 6681 |
| ENSG00000224984 | RP11-524H19.2 | AS | 5.186 | 10.945 | 1.05 | 0.00E+00 | 23702 |
| ENSG00000225484 | RP11-773D16.1 | lincRNA | 0.359 | 1.026 | 2.22 | 6.54E-04 | 467661 |
| ENSG00000225742 | RP11-513G11.4 | lincRNA | 0.069 | 0.306 | 2.17 | 2.15E-03 | 73890 |
| ENSG00000225778 | PROSER2-AS1 | AS | 0.165 | 0.036 | -2.20 | 2.86E-06 | 62976 |
| ENSG00000225886 | RP11-288L9.4 | AS | 2.050 | 5.822 | 1.64 | 1.11E-16 | 127310 |
| ENSG00000226330 | RP11-739N20.2 | AS | 3.803 | 1.582 | -1.46 | 8.24E-08 | 2800 |
| ENSG00000226471 | CTA-292E10.6 | AS | 2.914 | 1.300 | -1.16 | 9.29E-04 | 8560 |
| ENSG00000226920 | RP5-1068B5.3 | lincRNA | 0.557 | 0.137 | -2.39 | 2.28E-03 | 58956 |
| ENSG00000226950 | DANCR | lincRNA | 50.023 | 27.189 | -0.92 | 0.00E+00 | >1000000 |
| ENSG00000227121 | RP11-319F12.2 | lincRNA | 0.189 | 0.867 | 2.63 | 8.11E-06 | >1000000 |
| ENSG00000227218 | RP11-203J24.8 | AS | 15.649 | 6.515 | -1.31 | 5.50E-05 | 21293 |
| ENSG00000227256 | MIS18A-AS1 | AS | 0.387 | 0.085 | -2.40 | 1.76E-03 | 115671 |
| ENSG00000227285 | RP3-327A19.5 | AS | 0.039 | 0.364 | 3.68 | 4.39E-05 | 453463 |
| ENSG00000227359 | AC017074.2 | lincRNA | 0.810 | 2.129 | 1.36 | 1.36E-06 | 318442 |
| ENSG00000227512 | RP11-413M3.4 | AS | 0.363 | 0.019 | -4.88 | 3.03E-07 | 150995 |
| ENSG00000227527 | RP11-223A3.1 | SO | 0.545 | 0.049 | -4.16 | 3.34E-08 | 186060 |
| ENSG00000227542 | AC092614.2 | lincRNA | 0.948 | 2.160 | 1.12 | 1.76E-06 | 459386 |
| ENSG00000228315 | GUSBP11 | PT | 0.004 | 0.069 | 3.19 | 4.93E-06 | 524868 |
| ENSG00000228701 | TNKS2-AS1 | AS | 1.518 | 0.564 | -1.60 | 1.17E-04 | 126692 |
| ENSG00000228791 | THRB-AS1 | AS | 0.167 | 0.457 | 0.38 | 4.56E-08 | 225047 |
| ENSG00000229109 | RP11-439K3.1 | AS | 0.988 | 0.368 | -1.91 | 7.25E-04 | 177416 |
| ENSG00000229124 | VIM-AS1 | AS | 0.220 | 0.702 | 1.47 | 1.02E-04 | 215813 |
| ENSG00000229953 | RP11-284F21.7 | AS | 1.839 | 0.626 | -1.57 | 4.12E-10 | 44762 |
| ENSG00000230316 | FEZF1-AS1 | AS | 0.171 | 0.055 | -2.01 | 5.75E-04 | >1000000 |
| ENSG00000230392 | RP5-1139I1.1 | lincRNA | 0.027 | 0.124 | 2.04 | 6.63E-04 | 18515 |
| ENSG00000230658 | KLHL7-AS1 | lincRNA | 0.061 | 0.006 | -3.94 | 7.80E-04 | 229143 |
| ENSG00000231691 | RP11-203F10.5 | AS | 1.440 | 0.587 | -1.38 | 1.56E-03 | 103645 |
| ENSG00000231993 | RP1-85F18.5 | AS | 1.234 | 0.465 | -1.56 | 9.93E-04 | 464555 |
| ENSG00000232124 | AP001057.1 | AS | 1.980 | 0.942 | -1.08 | 5.97E-04 | 4580 |
| ENSG00000232710 | RP4-669P10.16 | SI | 0.047 | 0.270 | 2.42 | 5.14E-04 | 33795 |
| ENSG00000232860 | SMG7-AS1 | lincRNA | 0.318 | 0.140 | -1.12 | 1.49E-03 | 447769 |
| ENSG00000233013 | FAM157B | lincRNA | 0.017 | 0.100 | 1.07 | 1.93E-04 | 698173 |
| ENSG00000233016 | SNHG7 | AS | 35.886 | 23.901 | -0.64 | 0.00E+00 | 34308 |
| ENSG00000233052 | RP11-398B16.2 | SI | 1.222 | 0.319 | -2.32 | 4.71E-04 | 92587 |
| ENSG00000233077 | RP11-290F20.2 | lincRNA | 0.091 | 0.022 | -1.88 | 1.50E-04 | 6252 |
| ENSG00000233633 | AC093326.3 | lincRNA | 0.226 | 0.751 | 1.70 | 6.65E-04 | 8946 |
| ENSG00000233654 | AC093388.3 | AS | 0.853 | 0.202 | -2.32 | 2.78E-04 | 115839 |
| ENSG00000233806 | AC131097.3 | AS | 0.929 | 0.349 | -1.55 | 1.92E-03 | 614330 |
| ENSG00000233834 | AC005083.1 | lincRNA | 0.762 | 0.287 | -2.24 | 4.15E-06 | 3448 |
| ENSG00000233901 | RP11-65J3.1 | lincRNA | 5.094 | 2.397 | -1.22 | 2.13E-13 | 7221 |
| ENSG00000233903 | Z83851.4 | lincRNA | 2.529 | 0.841 | -1.49 | 3.52E-05 | 102628 |
| ENSG00000233930 | KRTAP5-AS1 | AS | 1.425 | 2.817 | 0.84 | 0.00E+00 | 198038 |
| ENSG00000233997 | AP000475.2 | lincRNA | 0.206 | 0.667 | 1.61 | 2.97E-03 | 431526 |
| ENSG00000234793 | AC114730.7 | AS | 0.188 | 0.024 | -3.26 | 3.92E-05 | 485971 |
| ENSG00000235027 | AC068580.6 | AS | 1.384 | 0.585 | -1.14 | 0.00E+00 | 9043 |
| ENSG00000235123 | DSCAM-AS1 | AS | 394.146 | 254.178 | -0.65 | 0.00E+00 | 316 |
| ENSG00000235236 | RP13-131K19.1 | AS | 1.891 | 0.472 | -1.68 | 3.45E-06 | 434976 |
| ENSG00000236519 | AL773604.8 | AS | 0.319 | 0.063 | -2.58 | 1.56E-04 | 28854 |
| ENSG00000237187 | NR2F1-AS1 | AS | 0.074 | 0.276 | 1.55 | 7.24E-07 | 593914 |
| ENSG00000237232 | ZNF295-AS1 | lincRNA | 0.109 | 0.028 | -1.83 | 2.48E-03 | 47577 |
| ENSG00000237248 | LINC00987 | lincRNA | 0.013 | 0.073 | 2.24 | 4.70E-05 | 107608 |
| ENSG00000237773 | AC003075.4 | AS | 0.131 | 0.727 | 2.49 | 1.44E-05 | 72894 |
| ENSG00000237807 | RP11-400K9.4 | lincRNA | 6.903 | 12.448 | 0.78 | 0.00E+00 | >1000000 |
| ENSG00000238184 | CD81-AS1 | AS | 0.193 | 0.045 | -2.30 | 2.90E-04 | 184539 |
| ENSG00000240050 | RP1-93H18.1 | lincRNA | 0.039 | 0.215 | 2.81 | 7.85E-06 | 206046 |
| ENSG00000240207 | RP11-379F4.4 | AS | 0.050 | 0.195 | 2.63 | 2.31E-04 | >1000000 |
| ENSG00000242687 | AC004893.11 | AS | 1.351 | 0.424 | -1.72 | 3.00E-05 | 177744 |
| ENSG00000242828 | RP11-47P18.1 | lincRNA | 0.661 | 0.081 | -3.57 | 2.39E-05 | 945666 |
| ENSG00000245080 | MIR3150A | AS | 0.434 | 1.019 | 1.13 | 1.42E-09 | 1895 |
| ENSG00000245146 | LINC01024 | AS | 0.317 | 0.680 | 1.18 | 4.50E-08 | 299347 |
| ENSG00000245614 | DDX11-AS1 | AS | 0.872 | 0.354 | -0.49 | 1.15E-12 | 386307 |
| ENSG00000246339 | EXTL3-AS1 | AS | 0.085 | 0.027 | -1.87 | 1.13E-03 | 553076 |
| ENSG00000248445 | CTB-118N6.3 | AS | 0.400 | 0.133 | -1.84 | 1.51E-04 | 910484 |
| ENSG00000248668 | OXCT1-AS1 | AS | 0.031 | 0.525 | 3.41 | 7.29E-06 | 437097 |
| ENSG00000248693 | CTD-2023M8.1 | lincRNA | 0.132 | 1.237 | 3.55 | 1.34E-04 | 988926 |
| ENSG00000248698 | LINC01085 | lincRNA | 0.071 | 0.412 | 2.48 | 6.06E-09 | >1000000 |
| ENSG00000249346 | LINC01016 | lincRNA | 1.233 | 0.301 | -2.05 | 0.00E+00 | 153 |
| ENSG00000249430 | CTD-2231H16.1 | lincRNA | 0.378 | 0.093 | -2.31 | 1.56E-03 | 42599 |
| ENSG00000249572 | CTD-2203K17.1 | AS | 2.859 | 1.158 | -1.48 | 4.36E-09 | 109603 |
| ENSG00000249685 | RP11-360F5.3 | lincRNA | 0.757 | 0.108 | -2.99 | 1.78E-05 | 381182 |
| ENSG00000249816 | LINC00964 | lincRNA | 0.049 | 0.005 | -3.57 | 9.24E-05 | 5414 |
| ENSG00000250012 | RP11-124N2.1 | AS | 2.717 | 0.700 | -1.80 | 1.62E-05 | 159239 |
| ENSG00000250237 | CTC-498J12.1 | lincRNA | 0.072 | 0.560 | 3.39 | 9.66E-05 | 286286 |
| ENSG00000250286 | RP11-94C24.8 | AS | 0.874 | 0.297 | -1.43 | 3.87E-11 | 221276 |
| ENSG00000250889 | RP11-229C3.2 | lincRNA | 0.147 | 0.582 | 2.01 | 3.36E-03 | 15446 |
| ENSG00000250906 | RP11-632F7.3 | AS | 0.099 | 0.446 | 1.82 | 2.36E-07 | 319684 |
| ENSG00000251359 | WWC2-AS2 | lincRNA | 0.050 | 0.210 | 2.40 | 6.34E-05 | 330359 |
| ENSG00000251513 | RP11-155G15.2 | PT | 0.105 | 0.385 | 2.02 | 2.48E-04 | >1000000 |
| ENSG00000251687 | RP11-181K12.2 | AS | 0.691 | 0.288 | -1.35 | 2.05E-03 | 96739 |
| ENSG00000253125 | RP11-459E5.1 | AS | 1.144 | 0.181 | -2.78 | 0.00E+00 | 44843 |
| ENSG00000253978 | CTB-178M22.2 | AS | 0.592 | 0.130 | -2.38 | 4.97E-04 | 84189 |
| ENSG00000254100 | RP11-675F6.4 | lincRNA | 0.758 | 0.274 | -1.74 | 1.78E-03 | 219519 |
| ENSG00000254248 | RP11-320N21.2 | AS | 0.612 | 0.185 | -1.94 | 6.79E-05 | 12847 |
| ENSG00000254290 | RP11-150O12.3 | lincRNA | 0.296 | 0.110 | -1.64 | 3.92E-05 | 1133 |
| ENSG00000254812 | RP11-661A12.12 | AS | 0.715 | 0.257 | -1.78 | 2.23E-04 | 616327 |
| ENSG00000254854 | CTD-2523D13.2 | AS | 1.438 | 0.657 | -1.21 | 1.92E-03 | 180595 |
| ENSG00000254864 | CTD-2516F10.4 | AS | 0.086 | 0.506 | 2.73 | 3.09E-04 | >1000000 |
| ENSG00000255031 | RP11-802E16.3 | AS | 0.693 | 0.145 | -1.81 | 1.66E-06 | 12378 |
| ENSG00000255100 | RP11-21L23.3 | AS | 1.259 | 0.389 | -2.39 | 1.37E-05 | 10277 |
| ENSG00000255198 | SNHG9 | lincRNA | 19.854 | 9.727 | -1.07 | 3.15E-03 | 51462 |
| ENSG00000255326 | CTD-2530H12.4 | AS | 0.048 | 0.327 | 3.36 | 4.24E-04 | 160562 |
| ENSG00000255571 | LINC00925 | lincRNA | 2.853 | 1.342 | -1.07 | 8.88E-16 | 23883 |
| ENSG00000256433 | RP1-102E24.8 | lincRNA | 0.303 | 0.881 | 1.77 | 3.47E-07 | 29874 |
| ENSG00000256982 | CTD-2555A7.2 | lincRNA | 0.342 | 0.129 | -1.48 | 7.20E-05 | 52883 |
| ENSG00000257588 | RP11-469H8.6 | AS | 2.173 | 0.698 | -1.69 | 4.70E-04 | 158726 |
| ENSG00000258584 | FAM181A-AS1 | AS | 0.150 | 0.375 | 1.44 | 7.40E-05 | 32592 |
| ENSG00000258811 | CTD-3051D23.1 | lincRNA | 0.380 | 0.055 | -2.88 | 1.71E-06 | 68078 |
| ENSG00000258821 | AC005041.17 | AS | 0.875 | 0.159 | -3.25 | 3.56E-07 | 346076 |
| ENSG00000259370 | RP11-1069G10.1 | AS | 0.063 | 0.390 | 2.63 | 1.51E-05 | 38439 |
| ENSG00000260260 | RP11-304L19.5 | lincRNA | 95.172 | 56.884 | -0.82 | 0.00E+00 | 95214 |
| ENSG00000260339 | HEXA-AS1 | AS | 0.135 | 0.361 | 1.39 | 8.59E-06 | 400502 |
| ENSG00000260417 | CTD-2542L18.1 | lincRNA | 0.141 | 0.005 | -6.42 | 5.18E-07 | 21677 |
| ENSG00000260729 | RP11-106M3.2 | PT | 0.004 | 0.037 | 3.95 | 6.10E-05 | 400370 |
| ENSG00000260807 | RP11-161M6.2 | lincRNA | 0.391 | 0.132 | -1.64 | 2.45E-09 | 67353 |
| ENSG00000261040 | CTD-2319I12.1 | lincRNA | 0.423 | 1.273 | 1.30 | 1.42E-03 | 607 |
| ENSG00000264198 | RP11-94L15.2 | lincRNA | 0.110 | 0.035 | -1.60 | 5.64E-06 | 417044 |
| ENSG00000264575 | LINC00526 | lincRNA | 0.292 | 0.838 | 1.39 | 5.06E-08 | >1000000 |
| ENSG00000265688 | MAFG-AS1 | AS | 1.620 | 0.725 | -1.23 | 8.51E-12 | 176236 |
| ENSG00000265962 | RP11-674N23.1 | AS | 0.345 | 0.040 | -3.17 | 1.09E-05 | 997813 |
| ENSG00000266256 | LINC00683 | lincRNA | 0.375 | 0.117 | -1.40 | 5.54E-06 | 71846 |
| ENSG00000266402 | SNORA76 | lincRNA | 17.129 | 6.827 | -1.48 | 1.93E-06 | 13996 |
| ENSG00000268061 | NAPA-AS1 | AS | 0.617 | 0.243 | -1.20 | 1.66E-05 | 147320 |
| ENSG00000269821 | KCNQ1OT1 | AS | 0.087 | 0.030 | -1.60 | 1.38E-05 | 300780 |
| ENSG00000273295 | AP000350.5 | lincRNA | 0.247 | 0.058 | -2.16 | 1.02E-04 | 673086 |

**Supplemental Table 1B:** **GRO-Seq analysis.** This Table reports the values of Log2 (Fold Change), with the associated p-value, derived from a published GRO-seq analyses 10, 25, 40 and 60 min after E2-treatment in MCF-7 cells (data from GSE43835, GSE41324, GSE45822 datasets).

| **Ensembl ID** | **Symbol** | **log2FC 10 min** | **log2FC 25 min** | **log2FC 40 min** | **log2FC 60 min** | **P-value 10 min** | **P-value 25 min** | **P-value 40 min** | **P-value 60 min** |
| --- | --- | --- | --- | --- | --- | --- | --- | --- | --- |
| ENSG00000100181 | TPTEP1 | -0.205 | -0.453 | -0.455 | 0.332 | 6.37E-01 | 4.89E-01 | 9.99E-01 | 9.28E-01 |
| ENSG00000130600 | H19 | 0.371 | 1.33 | 1.381 | 1.079 | 7.83E-01 | 5.03E-01 | 3.94E-01 | 3.42E-01 |
| ENSG00000132832 | RP11-445H22.3 | 0.053 | -0.243 | 0.818 | -0.882 | 9.99E-01 | 8.64E-01 | 8.60E-01 | 6.37E-01 |
| ENSG00000182586 | LINC00334 | 0.279 | -1.06 | 0.302 | -0.305 | 2.48E-01 | 5.00E-01 | 4.05E-01 | 1.00E+00 |
| ENSG00000185837 | CECR5-AS1 | 0.407 | 0.212 | 0.693 | 0.533 | 9.77E-01 | 6.31E-01 | 6.45E-01 | 5.37E-01 |
| ENSG00000189419 | SPATA41 | 0.476 | 1.094 | 0.828 | -0.206 | 3.34E-01 | 3.33E-02 | 5.54E-03 | 7.92E-01 |
| ENSG00000198106 | SNX29P2 | 0.531 | 0.094 | 1.446 | 0.834 | 1.50E-01 | 2.13E-01 | 4.80E-05 | 4.27E-01 |
| ENSG00000203999 | RP11-290F20.1 | -1.017 | 0.51 | -0.47 | 0.945 | 4.31E-01 | 6.76E-01 | 6.56E-01 | 4.01E-01 |
| ENSG00000204241 | RP11-713P17.3 | -0.282 | -0.582 | -0.896 | -0.346 | 3.99E-01 | 2.35E-01 | 1.10E-03 | 6.60E-01 |
| ENSG00000204261 | TAPSAR1 | 0.609 | -0.418 | 0.447 | -0.782 | 5.25E-01 | 6.32E-01 | 6.37E-01 | 7.68E-01 |
| ENSG00000206337 | HCP5 | 0.269 | -0.767 | 0.079 | -0.34 | 6.46E-02 | 2.26E-01 | 1.16E-01 | 7.06E-01 |
| ENSG00000214725 | CDIPT-AS1 | -0.074 | 0.18 | 0.389 | -0.917 | 9.97E-01 | 1.00E+00 | 9.95E-01 | 4.63E-01 |
| ENSG00000214733 | RP11-429J17.8 | -0.283 | -0.108 | -0.169 | 0.201 | 8.04E-01 | 1.00E+00 | 8.85E-01 | 9.77E-01 |
| ENSG00000223502 | RP11-96B5.3 | 1.526 | 0.552 | 0.304 | 0.628 | 7.38E-01 | 9.03E-01 | 9.68E-01 | 9.41E-01 |
| ENSG00000223669 | RP11-93B14.4 | 0.48 | 0 | 0.56 | 0.776 | 9.98E-01 | 1.00E+00 | 9.51E-01 | 7.52E-01 |
| ENSG00000223714 | RP5-1172N10.2 | -0.477 | -1.762 | -1.271 | -0.563 | 7.71E-01 | 1.10E-01 | 3.78E-02 | 6.16E-01 |
| ENSG00000223749 | MIR503HG | 1.488 | 2.101 | 2.998 | 1.121 | 4.02E-03 | 4.72E-04 | 5.94E-09 | 7.07E-02 |
| ENSG00000223808 | RP11-428L9.2 | -0.954 | -1.504 | -1.291 | -1.064 | 1.98E-03 | 5.91E-01 | 4.55E-04 | 7.63E-02 |
| ENSG00000224614 | TNK2-AS1 | 0.933 | 1.573 | 1.298 | -0.537 | 3.92E-03 | 1.62E-03 | 3.63E-06 | 6.52E-01 |
| ENSG00000224984 | RP11-524H19.2 | -0.206 | -1.693 | -0.006 | -1.013 | 7.03E-01 | 4.33E-01 | 5.10E-01 | 3.62E-01 |
| ENSG00000225484 | RP11-773D16.1 | -0.181 | 0.289 | 0.256 | 0.281 | 7.09E-01 | 1.00E+00 | 9.53E-01 | 7.89E-01 |
| ENSG00000225742 | RP11-513G11.4 | 1.035 | 0.904 | 0.865 | 0.713 | 5.21E-01 | 6.92E-01 | 7.80E-02 | 4.84E-01 |
| ENSG00000225778 | PROSER2-AS1 | 0.331 | 0.538 | 0.831 | -0.833 | 7.57E-01 | 3.48E-01 | 2.82E-02 | 8.23E-01 |
| ENSG00000225886 | RP11-288L9.4 | 0.298 | -0.768 | -0.421 | 0.683 | 9.97E-01 | 7.50E-01 | 8.74E-01 | 9.87E-01 |
| ENSG00000226330 | RP11-739N20.2 | 1.131 | 1.762 | 0.948 | 0.105 | 1.93E-01 | 1.56E-01 | 2.73E-01 | 9.99E-01 |
| ENSG00000226471 | CTA-292E10.6 | 0.899 | 0.393 | 0.688 | 0.704 | 3.82E-04 | 2.28E-01 | 6.84E-04 | 3.04E-01 |
| ENSG00000226920 | RP5-1068B5.3 | -0.899 | -1.189 | -0.331 | 0.225 | 2.45E-01 | 2.85E-01 | 7.90E-01 | 8.83E-01 |
| ENSG00000226950 | DANCR | 0.379 | 0.392 | 0.514 | -0.057 | 3.83E-01 | 6.96E-01 | 1.12E-01 | 9.18E-01 |
| ENSG00000227121 | RP11-319F12.2 | -0.344 | -0.198 | 0.595 | -0.099 | 9.98E-01 | 1.00E+00 | 1.00E+00 | 1.00E+00 |
| ENSG00000227218 | RP11-203J24.8 | 0.093 | 0.361 | 1.9 | 1.003 | 9.57E-01 | 2.46E-01 | 2.29E-14 | 4.08E-01 |
| ENSG00000227256 | MIS18A-AS1 | 0.454 | 0.609 | -0.248 | 0.052 | 8.42E-01 | 9.10E-01 | 4.67E-02 | 1.00E+00 |
| ENSG00000227285 | RP3-327A19.5 | 0.723 | 1.015 | 0.429 | -0.227 | 7.10E-01 | 6.48E-01 | 7.08E-01 | 7.10E-01 |
| ENSG00000227359 | AC017074.2 | -0.846 | -0.926 | -1.094 | -0.37 | 2.98E-01 | 3.79E-01 | 1.81E-01 | 7.82E-01 |
| ENSG00000227512 | RP11-413M3.4 | -1.089 | 0.138 | -0.136 | 0.089 | 8.41E-01 | 2.79E-01 | 6.90E-01 | 1.00E+00 |
| ENSG00000227527 | RP11-223A3.1 | -0.654 | -0.106 | -0.535 | -1.163 | 1.14E-01 | 5.58E-01 | 3.28E-01 | 7.13E-01 |
| ENSG00000227542 | AC092614.2 | -0.41 | -0.594 | -0.589 | 0.097 | 9.80E-01 | 5.53E-01 | 4.66E-01 | 1.00E+00 |
| ENSG00000228315 | GUSBP11 | -0.032 | -0.198 | 0.36 | 0.265 | 1.00E+00 | 1.00E+00 | 1.00E+00 | 1.00E+00 |
| ENSG00000228701 | TNKS2-AS1 | -0.531 | -0.947 | -0.632 | -0.809 | 3.01E-01 | 1.70E-01 | 3.27E-01 | 2.21E-01 |
| ENSG00000228791 | THRB-AS1 | -0.09 | -0.717 | -0.69 | -0.616 | 9.79E-01 | 3.43E-01 | 3.44E-01 | 4.54E-01 |
| ENSG00000229109 | RP11-439K3.1 | 0.52 | 1.262 | 0.65 | 0.01 | 1.03E-01 | 2.60E-02 | 1.30E-01 | 9.67E-01 |
| ENSG00000229124 | VIM-AS1 | 0.581 | 0.38 | 0.53 | -0.318 | 6.94E-02 | 1.00E+00 | 2.02E-01 | 6.12E-01 |
| ENSG00000229953 | RP11-284F21.7 | -0.916 | -0.928 | 0.016 | -0.302 | 4.49E-01 | 3.18E-01 | 4.51E-01 | 6.59E-01 |
| ENSG00000230316 | FEZF1-AS1 | 0.271 | 0.3 | -0.464 | 0 | 9.66E-01 | 9.34E-01 | 4.80E-01 | 1.00E+00 |
| ENSG00000230392 | RP5-1139I1.1 | -0.252 | -0.228 | 0.442 | 0.329 | 9.83E-01 | 9.90E-01 | 7.27E-01 | 9.69E-01 |
| ENSG00000230658 | KLHL7-AS1 | 0.066 | 0.232 | 0.272 | -0.232 | 8.64E-01 | 8.82E-01 | 2.90E-01 | 6.97E-01 |
| ENSG00000231691 | RP11-203F10.5 | 0.429 | -0.308 | 0.132 | 0 | 8.16E-01 | 7.82E-01 | 7.02E-01 | 1.00E+00 |
| ENSG00000231993 | RP1-85F18.5 | -0.175 | 0.493 | 0.373 | -0.694 | 3.71E-01 | 8.95E-01 | 9.54E-01 | 8.30E-01 |
| ENSG00000232124 | AP001057.1 | -0.222 | -0.308 | 0.458 | 1.353 | 7.53E-01 | 9.32E-01 | 1.24E-02 | 8.02E-02 |
| ENSG00000232710 | RP4-669P10.16 | -0.228 | 1.129 | 0.305 | -0.457 | 3.67E-01 | 7.94E-01 | 9.83E-01 | 1.00E+00 |
| ENSG00000232860 | SMG7-AS1 | 0.415 | 0.312 | 0.842 | 0.124 | 1.67E-01 | 6.62E-01 | 5.03E-03 | 7.97E-01 |
| ENSG00000233013 | FAM157B | -0.729 | -0.684 | -0.339 | 0 | 6.88E-01 | 6.55E-01 | 9.98E-01 | 1.00E+00 |
| ENSG00000233016 | SNHG7 | 0.124 | 0.544 | 0.138 | 0.236 | 8.64E-01 | 4.26E-01 | 7.84E-01 | 7.62E-01 |
| ENSG00000233052 | RP11-398B16.2 | -0.245 | 0 | 0.077 | 0 | 1.00E+00 | 1.00E+00 | 1.00E+00 | 1.00E+00 |
| ENSG00000233077 | RP11-290F20.2 | 0.432 | -1.401 | 0.249 | 0.24 | 9.45E-01 | 4.06E-01 | 9.95E-01 | 1.00E+00 |
| ENSG00000233633 | AC093326.3 | 0.165 | -1.396 | 0.412 | 0 | 6.98E-01 | 7.13E-02 | 8.37E-02 | 1.00E+00 |
| ENSG00000233654 | AC093388.3 | 0.168 | 0.19 | -0.425 | -0.142 | 9.90E-01 | 7.42E-01 | 9.48E-01 | 8.32E-01 |
| ENSG00000233806 | AC131097.3 | -0.429 | -1.23 | -2.192 | -0.807 | 6.56E-01 | 1.28E-01 | 3.19E-05 | 3.60E-01 |
| ENSG00000233834 | AC005083.1 | 0.135 | 0.678 | 0.97 | -0.162 | 2.12E-01 | 7.13E-01 | 1.68E-01 | 8.50E-01 |
| ENSG00000233901 | RP11-65J3.1 | -0.601 | -0.179 | 0.185 | -0.217 | 1.69E-01 | 8.61E-01 | 7.34E-01 | 7.18E-01 |
| ENSG00000233903 | Z83851.4 | 0.068 | 0.282 | 1.239 | 0.174 | 8.41E-01 | 1.00E+00 | 5.70E-01 | 9.05E-01 |
| ENSG00000233930 | KRTAP5-AS1 | -0.092 | 0.031 | -0.19 | -0.239 | 6.22E-01 | 7.51E-01 | 6.12E-01 | 8.89E-01 |
| ENSG00000233997 | AP000475.2 | -0.677 | -0.034 | 0.083 | -0.782 | 8.97E-01 | 9.60E-01 | 1.00E+00 | 7.68E-01 |
| ENSG00000234793 | AC114730.7 | 0.064 | -1.131 | -0.047 | 0 | 9.04E-01 | 6.35E-01 | 9.98E-01 | 1.00E+00 |
| ENSG00000235027 | AC068580.6 | 0.678 | 2.098 | 2.339 | 0 | 2.92E-01 | 1.69E-02 | 7.45E-09 | 1.00E+00 |
| ENSG00000235123 | DSCAM-AS1 | 0.54 | -0.667 | -0.198 | 0.95 | 4.83E-01 | 6.76E-01 | 5.71E-01 | 2.70E-01 |
| ENSG00000235236 | RP13-131K19.1 | 0.171 | -0.738 | -0.118 | -1.709 | 8.93E-01 | 8.73E-01 | 9.10E-01 | 7.97E-02 |
| ENSG00000236519 | AL773604.8 | 0.956 | 0.527 | 0.779 | 0.123 | 1.59E-03 | 5.10E-01 | 4.71E-02 | 7.65E-01 |
| ENSG00000237187 | NR2F1-AS1 | 0.687 | 0.727 | -0.062 | -1.24 | 7.97E-01 | 8.25E-01 | 6.85E-01 | 3.45E-01 |
| ENSG00000237232 | ZNF295-AS1 | 0.634 | 1.656 | 1.694 | 0.099 | 2.06E-01 | 4.78E-03 | 3.36E-05 | 9.20E-01 |
| ENSG00000237248 | LINC00987 | 1.034 | 0.207 | 0.947 | 0.477 | 2.70E-01 | 1.00E+00 | 8.51E-01 | 9.56E-01 |
| ENSG00000237773 | AC003075.4 | 1.288 | 0.738 | 1.109 | -1.066 | 1.08E-01 | 1.00E+00 | 9.79E-02 | 1.60E-01 |
| ENSG00000237807 | RP11-400K9.4 | -0.8 | -2.229 | -2.246 | -1.128 | 7.94E-01 | 4.49E-01 | 7.72E-03 | 4.25E-01 |
| ENSG00000238184 | CD81-AS1 | 0.429 | 1.729 | 0.513 | 0.266 | 6.85E-01 | 2.43E-01 | 8.38E-01 | 9.19E-01 |
| ENSG00000240050 | RP1-93H18.1 | -0.039 | 0.518 | -0.415 | 0.305 | 3.52E-01 | 8.87E-01 | 5.89E-01 | 7.39E-01 |
| ENSG00000240207 | RP11-379F4.4 | 0.616 | -1.655 | -0.444 | -0.586 | 2.15E-01 | 7.26E-02 | 7.74E-01 | 5.64E-01 |
| ENSG00000242687 | AC004893.11 | 0.238 | 0.714 | 0.354 | 0.043 | 5.43E-01 | 6.37E-01 | 3.60E-01 | 9.32E-01 |
| ENSG00000242828 | RP11-47P18.1 | -0.752 | 0.752 | 0.209 | 0.683 | 8.31E-01 | 9.14E-01 | 9.77E-01 | 9.87E-01 |
| ENSG00000245080 | MIR3150A | 0.246 | -1.476 | -1.428 | -1.326 | 8.85E-01 | 1.12E-01 | 1.71E-04 | 6.75E-02 |
| ENSG00000245146 | LINC01024 | -0.202 | -0.163 | -0.31 | -0.17 | 5.20E-01 | 6.91E-01 | 2.41E-01 | 8.25E-01 |
| ENSG00000245614 | DDX11-AS1 | -0.579 | -0.276 | -0.274 | 0.109 | 3.80E-01 | 3.26E-01 | 4.55E-01 | 8.33E-01 |
| ENSG00000246339 | EXTL3-AS1 | 0.016 | 0.047 | -0.252 | -0.137 | 9.14E-01 | 8.09E-01 | 3.35E-01 | 8.35E-01 |
| ENSG00000248445 | CTB-118N6.3 | -0.885 | -1.098 | -0.155 | 0.323 | 7.37E-01 | 9.06E-01 | 1.88E-01 | 7.68E-01 |
| ENSG00000248668 | OXCT1-AS1 | 0.015 | 0.408 | 0.025 | -0.066 | 6.82E-01 | 6.61E-01 | 5.69E-01 | 1.00E+00 |
| ENSG00000248693 | CTD-2023M8.1 | 0.312 | -0.115 | -1.154 | -0.554 | 7.04E-01 | 9.24E-01 | 5.27E-01 | 6.59E-01 |
| ENSG00000248698 | LINC01085 | -0.266 | 0.503 | 0.7 | 0.683 | 9.64E-01 | 1.00E+00 | 1.00E+00 | 9.87E-01 |
| ENSG00000249346 | LINC01016 | 3.233 | 4.505 | 5.752 | 3.684 | 5.83E-11 | 1.12E-07 | 0.00E+00 | 6.25E-06 |
| ENSG00000249430 | CTD-2231H16.1 | 0.488 | 1.165 | -0.099 | -0.75 | 9.66E-01 | 8.96E-01 | 9.97E-01 | 6.52E-01 |
| ENSG00000249572 | CTD-2203K17.1 | 0.014 | 0.733 | 0.491 | -0.25 | 8.69E-01 | 5.66E-01 | 5.35E-01 | 7.76E-01 |
| ENSG00000249685 | RP11-360F5.3 | -0.043 | -1.53 | -1.34 | -1.012 | 8.98E-01 | 5.44E-01 | 8.94E-02 | 4.18E-01 |
| ENSG00000249816 | LINC00964 | -0.232 | -0.207 | -0.161 | -0.457 | 3.96E-01 | 8.40E-02 | 5.72E-01 | 1.00E+00 |
| ENSG00000250012 | RP11-124N2.1 | 0.139 | 0.628 | -0.171 | -0.482 | 6.38E-01 | 9.27E-01 | 4.31E-01 | 7.19E-01 |
| ENSG00000250237 | CTC-498J12.1 | -0.212 | -0.7 | -1.728 | -1.535 | 7.32E-01 | 6.49E-01 | 4.24E-02 | 2.72E-01 |
| ENSG00000250286 | RP11-94C24.8 | -0.205 | -0.044 | -0.852 | 0 | 7.89E-01 | 5.42E-01 | 8.06E-02 | 1.00E+00 |
| ENSG00000250889 | RP11-229C3.2 | 0.673 | 1.164 | 0.755 | -0.36 | 6.83E-01 | 4.87E-01 | 6.49E-01 | 8.27E-01 |
| ENSG00000250906 | RP11-632F7.3 | 0.975 | 0.376 | 0.339 | 0.332 | 7.51E-01 | 9.18E-01 | 8.74E-01 | 9.28E-01 |
| ENSG00000251359 | WWC2-AS2 | 0.44 | 0.796 | -0.405 | -0.629 | 4.96E-01 | 3.88E-01 | 2.80E-01 | 5.93E-01 |
| ENSG00000251513 | RP11-155G15.2 | -0.152 | -0.37 | 0.275 | -0.251 | 4.13E-01 | 7.48E-01 | 6.32E-01 | 9.37E-01 |
| ENSG00000251687 | RP11-181K12.2 | -0.606 | 0.934 | -0.735 | 0 | 7.43E-01 | 9.31E-01 | 3.41E-01 | 1.00E+00 |
| ENSG00000253125 | RP11-459E5.1 | 1.517 | 2.272 | 3.24 | 2.873 | 4.70E-03 | 1.69E-03 | 1.11E-16 | 3.67E-05 |
| ENSG00000253978 | CTB-178M22.2 | 0 | 0 | 1.032 | 1.067 | 1.00E+00 | 1.00E+00 | 7.60E-01 | 4.08E-01 |
| ENSG00000254100 | RP11-675F6.4 | -0.587 | -2.277 | -2.8 | -1.29 | 2.06E-01 | 1.72E-02 | 5.99E-07 | 1.38E-01 |
| ENSG00000254248 | RP11-320N21.2 | -0.104 | -1.466 | -1.625 | -1.79 | 8.49E-01 | 8.07E-02 | 4.44E-05 | 7.89E-02 |
| ENSG00000254290 | RP11-150O12.3 | 1.656 | 2.09 | 2.072 | 2.161 | 3.09E-09 | 1.02E-04 | 1.11E-16 | 1.01E-01 |
| ENSG00000254812 | RP11-661A12.12 | 0.123 | 0.455 | -0.292 | -0.176 | 7.42E-01 | 8.35E-01 | 2.27E-01 | 8.16E-01 |
| ENSG00000254854 | CTD-2523D13.2 | -0.491 | -0.561 | -0.486 | -0.072 | 4.48E-01 | 3.78E-01 | 4.21E-01 | 1.00E+00 |
| ENSG00000254864 | CTD-2516F10.4 | 0.153 | 0.17 | -0.682 | -1.448 | 9.46E-01 | 7.29E-01 | 7.59E-01 | 2.03E-01 |
| ENSG00000255031 | RP11-802E16.3 | 0.043 | 0.914 | 0.163 | 1.929 | 7.97E-01 | 6.13E-01 | 9.84E-01 | 1.67E-02 |
| ENSG00000255100 | RP11-21L23.3 | 0.774 | -0.398 | 1.043 | 2.107 | 2.79E-01 | 8.74E-01 | 1.71E-05 | 1.07E-02 |
| ENSG00000255198 | SNHG9 | 0.296 | 0.597 | 0.539 | 0.1 | 7.22E-01 | 6.84E-01 | 2.17E-01 | 8.59E-01 |
| ENSG00000255326 | CTD-2530H12.4 | -0.129 | -0.446 | 0.089 | 0.265 | 8.22E-01 | 3.83E-01 | 8.05E-01 | 1.00E+00 |
| ENSG00000255571 | LINC00925 | -0.454 | 0.089 | -0.512 | -0.361 | 6.85E-02 | 7.26E-01 | 3.34E-01 | 5.78E-01 |
| ENSG00000256433 | RP1-102E24.8 | 0.356 | 0.87 | 0.482 | -0.064 | 6.36E-01 | 2.79E-01 | 4.88E-01 | 9.97E-01 |
| ENSG00000256982 | CTD-2555A7.2 | 0.364 | 0.233 | 1.995 | 1.182 | 8.70E-01 | 7.57E-01 | 1.48E-03 | 1.31E-01 |
| ENSG00000257588 | RP11-469H8.6 | 1.081 | 1.049 | 1.208 | -1.433 | 8.30E-01 | 7.69E-01 | 2.12E-01 | 3.95E-01 |
| ENSG00000258584 | FAM181A-AS1 | 0.46 | 0.837 | -0.099 | 0.533 | 1.00E+00 | 9.37E-01 | 9.99E-01 | 9.87E-01 |
| ENSG00000258811 | CTD-3051D23.1 | 0.006 | 0.05 | -0.511 | -0.42 | 9.87E-01 | 8.51E-01 | 7.90E-02 | 8.67E-01 |
| ENSG00000258821 | AC005041.17 | -0.515 | -0.459 | -0.594 | -1.091 | 2.43E-01 | 5.72E-01 | 3.01E-01 | 4.14E-01 |
| ENSG00000259370 | RP11-1069G10.1 | 2.289 | 2.087 | 3.553 | 2.57 | 3.56E-09 | 5.92E-03 | 0.00E+00 | 1.36E-01 |
| ENSG00000260260 | RP11-304L19.5 | 0.33 | 0.383 | 0.478 | -0.277 | 2.09E-01 | 4.05E-01 | 6.17E-02 | 6.65E-01 |
| ENSG00000260339 | HEXA-AS1 | -0.209 | -0.697 | -0.284 | -0.42 | 9.26E-01 | 6.48E-01 | 8.73E-01 | 4.82E-01 |
| ENSG00000260417 | CTD-2542L18.1 | -0.278 | 1.203 | 1.356 | 0.836 | 3.67E-01 | 1.26E-01 | 2.97E-04 | 2.27E-01 |
| ENSG00000260729 | RP11-106M3.2 | -0.154 | 0.652 | 0.315 | -0.418 | 2.76E-01 | 8.70E-01 | 8.75E-01 | 7.73E-01 |
| ENSG00000260807 | RP11-161M6.2 | 0.346 | 0.438 | -0.188 | 0.533 | 9.06E-01 | 9.96E-01 | 7.35E-01 | 9.87E-01 |
| ENSG00000261040 | CTD-2319I12.1 | -0.656 | -1.205 | -0.552 | -1.001 | 2.65E-01 | 6.92E-01 | 1.07E-01 | 2.34E-01 |
| ENSG00000264198 | RP11-94L15.2 | 0.324 | -0.704 | 0.223 | -0.238 | 5.25E-01 | 7.70E-01 | 5.46E-01 | 9.00E-01 |
| ENSG00000264575 | LINC00526 | 0.57 | -0.895 | 0.474 | 0 | 8.78E-01 | 4.04E-01 | 8.00E-01 | 1.00E+00 |
| ENSG00000265688 | MAFG-AS1 | 0.03 | -1.603 | -0.381 | 0.074 | 7.36E-01 | 1.02E-01 | 8.35E-01 | 1.00E+00 |
| ENSG00000265962 | RP11-674N23.1 | 0.337 | 1.45 | 0.396 | 0.533 | 9.24E-01 | 5.45E-01 | 1.00E+00 | 9.87E-01 |
| ENSG00000266256 | LINC00683 | -0.364 | -0.979 | -0.814 | -0.449 | 7.77E-01 | 1.96E-01 | 1.09E-01 | 6.13E-01 |
| ENSG00000266402 | SNORA76 | -0.219 | 0.576 | -0.051 | -0.064 | 5.43E-01 | 4.30E-01 | 8.90E-01 | 9.54E-01 |
| ENSG00000268061 | NAPA-AS1 | -0.047 | 0.036 | -0.379 | -0.183 | 4.29E-01 | 6.76E-01 | 6.47E-01 | 8.29E-01 |
| ENSG00000269821 | KCNQ1OT1 | 0.26 | -0.665 | 0.109 | 0.225 | 5.35E-01 | 6.74E-01 | 7.35E-01 | 6.65E-01 |
| ENSG00000273295 | AP000350.5 | 0.407 | -0.054 | -0.241 | -0.321 | 3.02E-01 | 8.73E-01 | 4.01E-01 | 6.67E-01 |

**Supplemental Table 2A:** **Analyzed public dataset references.** The Table reports the experimental identifier (e.g. GEO number ID), the experimental conditions and the assay type for each dataset analyzed.

| **ChIP-Seq data** |  |  |  |
| --- | --- | --- | --- |
| **Dataset ID** | **Condition or treatment** | **Factor/modification** | **Assay** |
| GSM588927 | Vehicle | AP2γ | ChIP-Seq |
| GSM822301 | Vehicle | c-Myc | ChIP-Seq |
| GSM822308 | Vehicle | CTCF | ChIP-Seq |
| GSM1295590 | Vehicle + control siRNA | ERα | ChIP-Seq |
| GSM986065 | Vehicle | FOXA1 | ChIP-Seq |
| GSM1000995 | Vehicle | FOXM1 | ChIP-Seq |
| GSM986068 | Vehicle | GATA3 | ChIP-Seq |
| GSM986079 | Vehicle | H3K27ac | ChIP-Seq |
| GSM916106 | Vehicle | H3K36me3 | ChIP-Seq |
| GSM986083 | Vehicle | H3K4me1 | ChIP-Seq |
| GSM588571 | Vehicle | H3K4me3 | ChIP-Seq |
| GSM986087 | Vehicle | p300 | ChIP-Seq |
| GSM1116656 | Vehicle | RNAPII | ChIP-Seq |

| **Expression Data** |  |  |  |
| --- | --- | --- | --- |
|  |  |  |  |
| **Data ID or http reference** | **Condition or treatment** | **Cell line / Tissue** | **Assay** |
| GSM1295593 | Vehicle + control siRNA | MCF-7 | RNA-Seq |
| GSM1295594 | Vehicle + siRNA ER | MCF-7 | RNA-Seq |
| GSM1067410,GSM1067411 | Vehicle | MCF-7 | GRO-Seq |
| GSM1067412,GSM1067413 | E2 10 min | MCF-7 | GRO-Seq |
| GSM1067414,GSM1067415 | E2 40 min | MCF-7 | GRO-Seq |
| GSM1014637,GSM1014638,GSM1014639 | Vehicle | MCF-7 | GRO-Seq |
| GSM1014640,GSM1014641,GSM1014643 | E2 10 min | MCF-7 | GRO-Seq |
| GSM1014643,GSM1014644,GSM1014645 | E2 25 min | MCF-7 | GRO-Seq |
| GSM1014645,GSM1014646,GSM1014647 | E2 40 min | MCF-7 | GRO-Seq |
| GSM1115997,GSM1115998 | Vehicle | MCF-7 | GRO-Seq |
| GSM1115995,GSM1115996 | E2 60 min | MCF-7 | GRO-Seq |
| GSM1172844,GSM1172845,GSM1172846,GSM1172847,GSM1172848,GSM1172849,GSM1172850,GSM1172851,GSM1172853,GSM1172854,GSM1172855,GSM1172856,GSM1172858,GSM1172859,GSM1172860,GSM1172861,GSM1172863,GSM1172864,GSM1172865,GSM1172867,GSM1172868,GSM1172869,GSM1172870,GSM1172871,GSM1172872,GSM1172873,GSM1172874,GSM1172875,GSM1172876,GSM1172877,GSM1172878,GSM1172879,GSM1172881,GSM1172882,GSM1172883,GSM1172884,GSM1172885,GSM1172886,GSM1172888,GSM1172889,GSM1172890,GSM1172893,GSM1172895,GSM1172896,GSM1172897,GSM1172901,GSM1172902,GSM1172903,GSM1172904,GSM1172906,GSM1172907,GSM1172908,GSM1172909,GSM1172910,GSM1384316 | Various growth conditions | 55 Breast cancer cell lines | RNA-Seq |
| GSM1401676,GSM1401677,GSM1401678,GSM1401679,GSM1401680,GSM1401681,GSM1401682,GSM1401683,GSM1401684,GSM1401685,GSM1401686,GSM1401687,GSM1401688,GSM1401689,GSM1401690,GSM1401691,GSM1401692,GSM1401693,GSM1401694,GSM1401695,GSM1401696,GSM1401697,GSM1401698,GSM1401699,GSM1401700,GSM1401701,GSM1401702,GSM1401703,GSM1401704,GSM1401705,GSM1401706,GSM1401707,GSM1401708,GSM1401709,GSM1401710,GSM1401711,GSM1401712,GSM1401713,GSM1401714,GSM1401715,GSM1401716,GSM1401717,GSM1401718,GSM1401719,GSM1401720,GSM1401721,GSM1401722,GSM1401723,GSM1401724,GSM1401725,GSM1401726,GSM1401727,GSM1401728,GSM1401729,GSM1401730,GSM1401731,GSM1401732,GSM1401733,GSM1401734,GSM1401735,GSM1401736,GSM1401737,GSM1401738,GSM1401739,GSM1401740,GSM1401741,GSM1401742,GSM1401743,GSM1401744,GSM1401745,GSM1401746,GSM1401747,GSM1401748,GSM1401749,GSM1401750,GSM1401751,GSM1401752,GSM1401753,GSM1401754,GSM1401755,GSM1401756,GSM1401757,GSM1401758,GSM1401759,GSM1401760,GSM1401761,GSM1401762,GSM1401763,GSM1401764,GSM1401765,GSM1401766,GSM1401767,GSM1401768,GSM1401769,GSM1401770,GSM1401771,GSM1401772,GSM1401773,GSM1401774,GSM1401775,GSM1401776,GSM1401777,GSM1401778,GSM1401779,GSM1401780,GSM1401781,GSM1401782,GSM1401783,GSM1401784,GSM1401785,GSM1401786,GSM1401787,GSM1401788,GSM1401789,GSM1401790,GSM1401791,GSM1401792,GSM1401793,GSM1401794,GSM1401795,GSM1401796,GSM1401797,GSM1401798,GSM1401799,GSM1401800,GSM1401801,GSM1401802,GSM1401803,GSM1401804,GSM1401805,GSM1401806,GSM1401807,GSM1401808,GSM1401809,GSM1401810,GSM1401811,GSM1401812,GSM1401813,GSM1401814,GSM1401815 | Fresh frozen breast cancer specimens | 140 breast cancer and normal tissues | RNA-Seq |
| http://mitranscriptome.org/ | Various growth conditions | 6,249 tissues | RNA-Seq |
| GSM1150560,GSM1150561,GSM1150562,GSM1150563,GSM1150564,GSM1150565,GSM1150566,GSM1150567,GSM1150568,GSM1150569,GSM1150570,GSM1150571,GSM1150572,GSM1150573,GSM1150574,GSM1150575,GSM1150576,GSM1150577,GSM1150578,GSM1150579,GSM1150580,GSM1150581,GSM1150582,GSM1150583,GSM1150584,GSM1150585,GSM1150586,GSM1150587,GSM1150588,GSM1150589,GSM1150590,GSM1150591,GSM1150592,GSM1150593,GSM1150594,GSM1150595,GSM1150596,GSM1150597,GSM1150598,GSM1150599,GSM1150600,GSM1150601,GSM1150602,GSM1150603,GSM1150604,GSM1150605,GSM1150606,GSM1150607,GSM1150608,GSM1150609,GSM1150610,GSM1150611,GSM1150612,GSM1150613,GSM1150614,GSM1150615,GSM1150616,GSM1150617,GSM1150618,GSM1150619,GSM1150620,GSM1150621,GSM1150622,GSM1150623,GSM1150624,GSM1150625,GSM1150626,GSM1150627,GSM1150628,GSM1150629,GSM1150630,GSM1150631 | FFPE breast cancer specimens | 72 breast cancer and normal tissues | 3SEQ |
| http://www.broadinstitute.org/ccle/home | Various growth conditions | 1,037 cell lines | Microarray |
| GSE58252,GSE27473,GSE13477,GSE28789,GSE28789,GSE8597,GSE29672,GSE29672,GSE8565,GSE29672,GSE45046,GSE29672,GSE29137,GSE40968,GSE29137,GSE28789,GSE15481,GSE26599,GSE13477,GSE31782,GSE26599,GSE29672,GSE40968,GSE26298,GSE8597,GSE53668,GSE37820,GSE26599,GSE35428,GSE41972,GSE31912,GSE10890,GSE5823,GSE24547,GSE36529,GSE53668,GSE46924,GSE31912,GSE13477,GSE31912,GSE29672,GSE46924,GSE40985,GSE31180,GSE8565,GSE46924,GSE11352,GSE31912,GSE31782,GSE18552,GSE53668,GSE14987,GSE5823,GSE35428,GSE40730,GSE31912,GSE26298,GSE17508,GSE53668 | Various growth conditions | MCF-7, MCF-10A, SK-BR-3, BT-474, MDA-MB-453, T-47D | Microarray |

**Supplemental Table 3:** **Weka confusion matrices.** The number of correctly classified breast cancer cell lines is reported for each subset of AER-lncRNAs considered.

| **All AER-lncRNAs** |  |  |  |  |
| --- | --- | --- | --- | --- |
| Correctly Classified Instances: 53 (96.3636 %) |  |  |  |  |
|  |  |  |  |  |
| **Confusion matrix** |  |  |  |  |
| **Normal-like** | **Basal** | **Luminal** | **Claudin-low** | **<-- classified as** |
| 5 | 0 | 0 | 0 | **Normal-like** |
| 0 | 16 | 0 | 1 | **Basal** |
| 0 | 1 | 26 | 0 | **Luminal** |
| 0 | 0 | 0 | 6 | **Claudin-low** |

| **AER-lncRNAs without 29-AER-lncRNA signature** |  |  |  |  |
| --- | --- | --- | --- | --- |
| Correctly Classified Instances: 44 (80 %) |  |  |  |  |
|  |  |  |  |  |
| **Confusion matrix** |  |  |  |  |
| **Normal-like** | **Basal** | **Luminal** | **Claudin-low** | **<-- classified as** |
| 2 | 1 | 1 | 1 | **Normal-like** |
| 0 | 15 | 1 | 1 | **Basal** |
| 1 | 1 | 25 | 0 | **Luminal** |
| 0 | 4 | 0 | 2 | **Claudin-low** |

| **29-AER-lncRNA signature only** |  |  |  |  |
| --- | --- | --- | --- | --- |
| Correctly Classified Instances: 53 (96.3636 %) |  |  |  |  |
|  |  |  |  |  |
| **Confusion matrix** |  |  |  |  |
| **Normal-like** | **Basal** | **Luminal** | **Claudin-low** | **<-- classified as** |
| 5 | 0 | 0 | 0 | **Normal-like** |
| 0 | 16 | 1 | 0 | **Basal** |
| 0 | 1 | 26 | 0 | **Luminal** |
| 0 | 0 | 0 | 6 | **Claudin-low** |

**Supplemental Table 4: Breast tumor data.** Histology, grade, pT and pN values, immunohistochemistry (IHC) of ERα, PR and HER2, score FISH number of HER2 are reported for the 42 tumor biopsies RNA analyzed by qRT-PCR (Figure 4G).

| **Histology number** | **Diagnosis** | **Grade** | **pT** | **pN** | **IHC ER** | **IHC PR** | **IHC HER2 score** | **FISH HER2** |
| --- | --- | --- | --- | --- | --- | --- | --- | --- |
| BC_1 | IDC | 3 | 3 | 1a | - | 0 | 0 | NA |
| BC_2 | IDC | 3 | 1c | 1a | - | 0 | 1 | NA |
| BC_3 | IDC | 3 | 2 | 2a | - | 0 | 0 | NA |
| BC_4 | IDC | 3 | 2(m) | 0 sn (i-) * | - | 0 | 0 | NA |
| BC_5 | IDC | 3 | 2 | 0 | - | 0 | 3 | NA |
| BC_6 | IDC | 3 | 2m | 0 (i-) sn* | - | 0 | 1 | NA |
| BC_7 | IDC | 3 | 2 | 1a | - | 0 | 0 | NA |
| BC_8 | IDC | 3 | 2 | 0 (i-) sn* | - | 0 | 0 | NA |
| BC_9 | Metaplastic Carcinoma | 3 | 2 | 1a* | - | 0 | 0 | NA |
| BC_10 | IDC | 3 | 1c | 1a* | - | 0 | 0 | NA |
| BC_11 | ILC | 3 | 2 | 1a | - | 0 | 0 | NA |
| BC_12 | IDC | 2 | 2 | 3a | + | 38 | 2 | no amplification |
| BC_13 | IDC | 2 | 1c | 1a | + | 55 | 1 | NA |
| BC_14 | IDC | 3 | 2 | 0 sn* | + | 95 | 1 | NA |
| BC_15 | IDC | 2 | 2 | 1a | + | 20 | 0 | NA |
| BC_16 | IDC | 2 | 1c | 1a* | + | 35 | 1 | NA |
| BC_17 | ILC | 2 | 3 | 3a | + | 70 | 3 | NA |
| BC_18 | IDC | 3 | 2 | 1a* | + | 25 | 0 | NA |
| BC_19 | IDC | 3 | 2 | 2a | + | 20 | 0 | NA |
| BC_20 | IDC | 2 | 1c | 0 (i-) sn* | + | 65 | 0 | NA |
| BC_21 | IDC | 3 | 2 | 1a* | + | 10 | 0 | NA |
| BC_22 | IDC | 3 | 1c | 1c | + | 70 | 1 | NA |
| BC_23 | DCIS | NA | is | NA | + | 0 | 3 | NA |
| BC_24 | IDC | 2 | 1c | 0 | - | 0 | 3 | NA |
| BC_25 | IDC | 3 | 4b | 0 | - | 0 | 3 | NA |
| BC_26 | IDC | 3 | 2 | 1a | - | 0 | 1 | NA |
| BC_27 | IDC | 3 | 2 | 0 (i+) sn* | - | 1 | 1 | NA |
| BC_28 | IDC | 3 | 2 | 0 sn)(i+) * | + | 30 | 3 | NA |
| BC_29 | IDC | 2 | 1c | 1mic (sn) + | + | 55 | 2 | no amplification |
| BC_30 | IDC | 3 | 3(m) | 3a | + | 10 | 3 | NA |
| BC_31 | IDC | 3 | 2 | 1a* | + | 60 | 2 | no amplification |
| BC_32 | IDC/ILC | 1 | 2 | 1a* | + | 90 | 2 | no amplification |
| BC_33 | IDC | 3 | 1c | 1a* | + | 60 | 3 | NA |
| BC_34 | IDC | 2 | 2 | 1a | + | 80 | 3 | NA |
| BC_35 | ILC | 2 | 2 | 0mi* | + | 98 | 1 | NA |
| BC_36 | IDC | 2 | 1c | 0(sn)* | + | 75 | 1 | NA |
| BC_37 | IDC | 2 | 1c | 2a | + | 50 | 2 | no amplification |
| BC_38 | IDC | 2 | 1c | 0 (i-) sn | + | 90 | 3 | NA |
| BC_39 | IDC | 3 | 2 | 0 (sn) (i-) * | + | 5 | 3 | NA |
| BC_40 | IDC | 2 | 2 | 0 (sn) (i-) * | + | 70 | 3 | NA |
| BC_41 | IDC | 2 | 1c | 0 (sn) (i-) * | + | 75 | 2 | no amplification |
| BC_42 | IDC | 2 | 1c | 0 | + | 25 | 3 | NA |

**Supplemental Table 5A: Correlation analysis.** List of DSCAM-AS1 correlated and anti-correlated genes from the 55 breast cancer cell lines dataset (Ref 29 maintext). Correlation values and p-values are reported for each gene. Pt.cod = protein coding; PSG = pseudogene; AS = antisense; SI = sense intronic; SO = sense overlapping; PT = processed transcript.

| **Ensembl ID** | **Symbol** | **Correlation** | **P-value** | **Biotype** | **Ensembl ID** | **Symbol** | **Correlation** | **P-value** | **Biotype** |
| --- | --- | --- | --- | --- | --- | --- | --- | --- | --- |
| ENSG00000131149 | GSE1 | 0,842 | 2,22E-15 | pt.cod | ENSG00000142606 | MMEL1 | 0,713 | 1,99E-09 | pt.cod |
| ENSG00000167964 | RAB26 | 0,822 | 3,39E-13 | pt.cod | ENSG00000183067 | IGSF5 | 0,712 | 2,97E-09 | pt.cod |
| ENSG00000139531 | SUOX | 0,818 | 2,80E-13 | pt.cod | ENSG00000261452 | RP11-509E16.1 | 0,711 | 1,96E-09 | lincRNA |
| ENSG00000179627 | ZBTB42 | 0,81 | 3,05E-12 | pt.cod | ENSG00000175985 | PLEKHD1 | 0,71 | 4,15E-09 | pt.cod |
| ENSG00000160180 | TFF3 | 0,809 | 1,20E-13 | pt.cod | ENSG00000172794 | RAB37 | 0,709 | 4,74E-09 | pt.cod |
| ENSG00000064787 | BCAS1 | 0,808 | 1,66E-13 | pt.cod | ENSG00000148396 | SEC16A | 0,709 | 1,25E-08 | pt.cod |
| ENSG00000160182 | TFF1 | 0,807 | 2,86E-13 | pt.cod | ENSG00000143842 | SOX13 | 0,708 | 6,12E-09 | pt.cod |
| ENSG00000214530 | STARD10 | 0,807 | 4,76E-13 | pt.cod | ENSG00000183044 | ABAT | 0,708 | 8,06E-09 | pt.cod |
| ENSG00000143412 | ANXA9 | 0,807 | 9,03E-13 | pt.cod | ENSG00000204323 | SMIM5 | 0,708 | 1,19E-08 | pt.cod |
| ENSG00000170476 | MZB1 | 0,804 | 2,96E-13 | pt.cod | ENSG00000132746 | ALDH3B2 | 0,707 | 3,01E-09 | pt.cod |
| ENSG00000074370 | ATP2A3 | 0,803 | 1,02E-12 | pt.cod | ENSG00000165731 | RET | 0,707 | 4,77E-09 | pt.cod |
| ENSG00000163704 | PRRT3 | 0,801 | 8,82E-12 | pt.cod | ENSG00000253125 | RP11-459E5.1 | 0,707 | 6,45E-09 | PT |
| ENSG00000124664 | SPDEF | 0,8 | 2,74E-13 | pt.cod | ENSG00000130584 | ZBTB46 | 0,707 | 5,97E-08 | pt.cod |
| ENSG00000198133 | TMEM229B | 0,8 | 1,32E-12 | pt.cod | ENSG00000172725 | CORO1B | 0,706 | 2,67E-08 | pt.cod |
| ENSG00000116299 | KIAA1324 | 0,797 | 7,66E-13 | pt.cod | ENSG00000257883 | RP11-497G19.1 | 0,705 | 3,06E-09 | lincRNA |
| ENSG00000268615 | RP11-65J3.15 | 0,797 | 9,91E-13 | lincRNA | ENSG00000159423 | ALDH4A1 | 0,705 | 3,45E-09 | pt.cod |
| ENSG00000255735 | AC110619.1 | 0,795 | 6,67E-13 | PSG | ENSG00000110169 | HPX | 0,705 | 4,13E-09 | pt.cod |
| ENSG00000218416 | AC110619.2 | 0,793 | 9,23E-13 | pt.cod | ENSG00000205913 | SRRM2-AS1 | 0,705 | 2,46E-08 | AS |
| ENSG00000109062 | SLC9A3R1 | 0,789 | 7,85E-12 | pt.cod | ENSG00000108175 | ZMIZ1 | 0,705 | 1,71E-07 | pt.cod |
| ENSG00000124126 | PREX1 | 0,788 | 3,88E-12 | pt.cod | ENSG00000162078 | ZG16B | 0,704 | 7,01E-09 | pt.cod |
| ENSG00000235939 | RP11-123B3.2 | 0,788 | 4,06E-12 | AS | ENSG00000124493 | GRM4 | 0,703 | 9,09E-09 | pt.cod |
| ENSG00000228613 | AC144450.1 | 0,788 | 4,22E-12 | AS | ENSG00000265246 | RP11-663N22.1 | 0,701 | 3,04E-09 | lincRNA |
| ENSG00000085831 | TTC39A | 0,787 | 2,92E-12 | pt.cod | ENSG00000260710 | RP11-616M22.7 | 0,701 | 4,90E-09 | lincRNA |
| ENSG00000261664 | RP11-275F13.1 | 0,786 | 4,12E-12 | AS | ENSG00000135773 | CAPN9 | 0,701 | 6,01E-09 | pt.cod |
| ENSG00000149809 | TM7SF2 | 0,786 | 1,89E-11 | pt.cod | ENSG00000115363 | EVA1A | -0,7 | 1,35E-09 | pt.cod |
| ENSG00000211689 | TRGC1 | 0,785 | 3,90E-12 | TR_C_gene | ENSG00000107249 | GLIS3 | -0,7 | 7,85E-09 | pt.cod |
| ENSG00000247011 | RP11-700H6.1 | 0,784 | 9,81E-12 | lincRNA | ENSG00000171658 | RP11-443P15.2 | -0,701 | 5,24E-10 | PSG |
| ENSG00000179082 | C9orf106 | 0,783 | 8,59E-12 | PT | ENSG00000255248 | RP11-166D19.1 | -0,701 | 8,84E-10 | SO |
| ENSG00000248954 | RP11-304F15.4 | 0,783 | 1,33E-11 | lincRNA | ENSG00000206075 | SERPINB5 | -0,701 | 1,86E-09 | pt.cod |
| ENSG00000115616 | SLC9A2 | 0,774 | 2,24E-11 | pt.cod | ENSG00000231298 | LINC00704 | -0,704 | 5,23E-10 | lincRNA |
| ENSG00000143578 | CREB3L4 | 0,772 | 4,56E-11 | pt.cod | ENSG00000177469 | PTRF | -0,704 | 5,52E-10 | pt.cod |
| ENSG00000185863 | TMEM210 | 0,771 | 1,28E-11 | pt.cod | ENSG00000041982 | TNC | -0,704 | 5,23E-09 | pt.cod |
| ENSG00000124507 | PACSIN1 | 0,77 | 5,95E-11 | pt.cod | ENSG00000183853 | KIRREL | -0,706 | 1,90E-09 | pt.cod |
| ENSG00000160325 | CACFD1 | 0,77 | 1,33E-10 | pt.cod | ENSG00000184838 | PRR16 | -0,706 | 4,54E-09 | pt.cod |
| ENSG00000167861 | HID1 | 0,769 | 1,87E-11 | pt.cod | ENSG00000073756 | PTGS2 | -0,706 | 1,14E-08 | pt.cod |
| ENSG00000176884 | GRIN1 | 0,769 | 3,82E-11 | pt.cod | ENSG00000180672 | AC007362.1 | -0,707 | 1,50E-10 | PSG |
| ENSG00000141232 | TOB1 | 0,769 | 1,30E-10 | pt.cod | ENSG00000149948 | HMGA2 | -0,707 | 1,06E-09 | pt.cod |
| ENSG00000203635 | AC144450.2 | 0,766 | 4,39E-11 | lincRNA | ENSG00000181773 | GPR3 | -0,707 | 3,80E-09 | pt.cod |
| ENSG00000153060 | TEKT5 | 0,766 | 5,25E-11 | pt.cod | ENSG00000135362 | PRR5L | -0,708 | 2,20E-08 | pt.cod |
| ENSG00000172602 | RND1 | 0,764 | 6,84E-11 | pt.cod | ENSG00000174125 | TLR1 | -0,708 | 2,47E-08 | pt.cod |
| ENSG00000223764 | RP11-54O7.3 | 0,76 | 3,57E-11 | lincRNA | ENSG00000240163 | RP11-745A24.1 | -0,71 | 1,74E-08 | PSG |
| ENSG00000143878 | RHOB | 0,76 | 1,38E-10 | pt.cod | ENSG00000105974 | CAV1 | -0,711 | 4,21E-10 | pt.cod |
| ENSG00000171302 | CANT1 | 0,76 | 7,51E-09 | pt.cod | ENSG00000255443 | RP1-68D18.4 | -0,711 | 1,17E-09 | AS |
| ENSG00000269356 | RP11-120K24.4 | 0,759 | 1,60E-10 | SI | ENSG00000144810 | COL8A1 | -0,711 | 1,68E-09 | pt.cod |
| ENSG00000100346 | CACNA1I | 0,756 | 1,57E-10 | pt.cod | ENSG00000136244 | IL6 | -0,712 | 5,25E-09 | pt.cod |
| ENSG00000139998 | RAB15 | 0,755 | 2,71E-10 | pt.cod | ENSG00000251442 | LINC01094 | -0,714 | 3,29E-10 | lincRNA |
| ENSG00000106077 | ABHD11 | 0,755 | 4,84E-10 | pt.cod | ENSG00000196754 | S100A2 | -0,715 | 2,91E-10 | pt.cod |
| ENSG00000168350 | DEGS2 | 0,752 | 9,39E-11 | pt.cod | ENSG00000163565 | IFI16 | -0,716 | 3,83E-10 | pt.cod |
| ENSG00000213918 | DNASE1 | 0,752 | 4,59E-09 | pt.cod | ENSG00000148468 | FAM171A1 | -0,716 | 1,15E-09 | pt.cod |
| ENSG00000129514 | FOXA1 | 0,75 | 1,05E-10 | pt.cod | ENSG00000079150 | FKBP7 | -0,716 | 6,37E-08 | pt.cod |
| ENSG00000110171 | TRIM3 | 0,749 | 3,86E-10 | pt.cod | ENSG00000233532 | LINC00460 | -0,717 | 1,68E-10 | lincRNA |
| ENSG00000171433 | GLOD5 | 0,746 | 2,38E-10 | pt.cod | ENSG00000197860 | SGTB | -0,719 | 6,54E-09 | pt.cod |
| ENSG00000187535 | IFT140 | 0,746 | 2,48E-08 | pt.cod | ENSG00000246228 | CASC8 | -0,723 | 3,23E-11 | AS |
| ENSG00000174233 | ADCY6 | 0,744 | 1,75E-10 | pt.cod | ENSG00000207870 | MIR221 | -0,723 | 2,80E-09 | miRNA |
| ENSG00000152642 | GPD1L | 0,743 | 1,07E-11 | pt.cod | ENSG00000170989 | S1PR1 | -0,725 | 2,90E-10 | pt.cod |
| ENSG00000165140 | FBP1 | 0,742 | 2,10E-10 | pt.cod | ENSG00000026508 | CD44 | -0,726 | 6,86E-10 | pt.cod |
| ENSG00000135709 | KIAA0513 | 0,74 | 5,07E-10 | pt.cod | ENSG00000158163 | DZIP1L | -0,726 | 2,27E-09 | pt.cod |
| ENSG00000171604 | CXXC5 | 0,74 | 5,30E-10 | pt.cod | ENSG00000147509 | RGS20 | -0,727 | 4,96E-10 | pt.cod |
| ENSG00000115705 | TPO | 0,74 | 7,12E-10 | pt.cod | ENSG00000113083 | LOX | -0,727 | 7,70E-10 | pt.cod |
| ENSG00000119711 | ALDH6A1 | 0,739 | 3,04E-10 | pt.cod | ENSG00000122870 | BICC1 | -0,727 | 5,70E-09 | pt.cod |
| ENSG00000057593 | F7 | 0,738 | 1,94E-10 | pt.cod | ENSG00000118515 | SGK1 | -0,728 | 4,82E-11 | pt.cod |
| ENSG00000084710 | EFR3B | 0,738 | 3,25E-10 | pt.cod | ENSG00000144642 | RBMS3 | -0,728 | 1,32E-09 | pt.cod |
| ENSG00000263586 | RP11-309N17.4 | 0,737 | 5,44E-10 | AS | ENSG00000251194 | RP1-68D18.2 | -0,732 | 7,87E-10 | SI |
| ENSG00000120885 | CLU | 0,736 | 3,17E-10 | pt.cod | ENSG00000169429 | IL8 | -0,733 | 4,39E-11 | pt.cod |
| ENSG00000114737 | CISH | 0,736 | 8,09E-10 | pt.cod | ENSG00000104332 | SFRP1 | -0,733 | 9,22E-11 | pt.cod |
| ENSG00000158158 | CNNM4 | 0,736 | 3,31E-09 | pt.cod | ENSG00000081041 | CXCL2 | -0,733 | 2,02E-10 | pt.cod |
| ENSG00000185442 | FAM174B | 0,735 | 3,98E-10 | pt.cod | ENSG00000166741 | NNMT | -0,733 | 2,62E-10 | pt.cod |
| ENSG00000017621 | MAGIX | 0,735 | 6,30E-10 | pt.cod | ENSG00000136231 | IGF2BP3 | -0,736 | 4,98E-10 | pt.cod |
| ENSG00000142197 | DOPEY2 | 0,735 | 1,63E-09 | pt.cod | ENSG00000241106 | HLA-DOB | -0,737 | 3,15E-09 | pt.cod |
| ENSG00000236008 | AC011747.4 | 0,735 | 3,79E-09 | lincRNA | ENSG00000242147 | RP13-463N16.6 | -0,74 | 9,56E-12 | lincRNA |
| ENSG00000196557 | CACNA1H | 0,733 | 2,76E-10 | pt.cod | ENSG00000168685 | IL7R | -0,74 | 2,81E-10 | pt.cod |
| ENSG00000072858 | SIDT1 | 0,733 | 3,58E-10 | pt.cod | ENSG00000176597 | B3GNT5 | -0,742 | 4,41E-12 | pt.cod |
| ENSG00000169710 | FASN | 0,733 | 1,42E-08 | pt.cod | ENSG00000085662 | AKR1B1 | -0,742 | 1,48E-10 | pt.cod |
| ENSG00000100219 | XBP1 | 0,732 | 2,73E-10 | pt.cod | ENSG00000139278 | GLIPR1 | -0,743 | 2,43E-11 | pt.cod |
| ENSG00000132677 | RHBG | 0,732 | 8,96E-10 | pt.cod | ENSG00000135919 | SERPINE2 | -0,746 | 5,08E-11 | pt.cod |
| ENSG00000108639 | SYNGR2 | 0,732 | 4,74E-09 | pt.cod | ENSG00000057657 | PRDM1 | -0,747 | 2,75E-11 | pt.cod |
| ENSG00000114770 | ABCC5 | 0,732 | 1,21E-08 | pt.cod | ENSG00000197646 | PDCD1LG2 | -0,748 | 5,55E-11 | pt.cod |
| ENSG00000182809 | CRIP2 | 0,731 | 4,51E-10 | pt.cod | ENSG00000166396 | SERPINB7 | -0,748 | 1,80E-10 | pt.cod |
| ENSG00000188833 | ENTPD8 | 0,731 | 9,36E-10 | pt.cod | ENSG00000091409 | ITGA6 | -0,751 | 1,84E-11 | pt.cod |
| ENSG00000237441 | RGL2 | 0,731 | 3,62E-09 | pt.cod | ENSG00000188641 | DPYD | -0,751 | 9,27E-11 | pt.cod |
| ENSG00000168421 | RHOH | 0,73 | 6,74E-10 | pt.cod | ENSG00000270069 | RP6-99M1.2 | -0,753 | 2,84E-12 | lincRNA |
| ENSG00000205018 | RP11-830F9.6 | 0,728 | 9,10E-10 | pt.cod | ENSG00000023445 | BIRC3 | -0,753 | 1,29E-11 | pt.cod |
| ENSG00000106012 | IQCE | 0,728 | 4,67E-08 | pt.cod | ENSG00000123685 | BATF3 | -0,756 | 5,85E-11 | pt.cod |
| ENSG00000173890 | GPR160 | 0,727 | 1,01E-09 | pt.cod | ENSG00000166401 | SERPINB8 | -0,761 | 5,63E-12 | pt.cod |
| ENSG00000181444 | ZNF467 | 0,724 | 1,17E-09 | pt.cod | ENSG00000111817 | DSE | -0,762 | 9,92E-12 | pt.cod |
| ENSG00000139865 | TTC6 | 0,724 | 1,75E-09 | pt.cod | ENSG00000187498 | COL4A1 | -0,769 | 1,82E-11 | pt.cod |
| ENSG00000170011 | MYRIP | 0,724 | 2,29E-09 | pt.cod | ENSG00000163734 | CXCL3 | -0,771 | 5,26E-12 | pt.cod |
| ENSG00000166816 | LDHD | 0,723 | 8,86E-10 | pt.cod | ENSG00000157827 | FMNL2 | -0,772 | 3,27E-14 | pt.cod |
| ENSG00000211695 | TRGV9 | 0,723 | 1,31E-09 | TR_V_gene | ENSG00000118257 | NRP2 | -0,773 | 1,02E-11 | pt.cod |
| ENSG00000141977 | CIB3 | 0,721 | 1,70E-09 | pt.cod | ENSG00000163661 | PTX3 | -0,773 | 1,71E-11 | pt.cod |
| ENSG00000187848 | P2RX2 | 0,72 | 1,59E-09 | pt.cod | ENSG00000090530 | LEPREL1 | -0,775 | 6,08E-12 | pt.cod |
| ENSG00000184709 | LRRC26 | 0,72 | 2,26E-09 | pt.cod | ENSG00000101384 | JAG1 | -0,777 | 1,22E-12 | pt.cod |
| ENSG00000117643 | MAN1C1 | 0,72 | 3,32E-09 | pt.cod | ENSG00000019549 | SNAI2 | -0,778 | 5,02E-13 | pt.cod |
| ENSG00000183036 | PCP4 | 0,719 | 1,44E-09 | pt.cod | ENSG00000164099 | PRSS12 | -0,779 | 3,22E-12 | pt.cod |
| ENSG00000222014 | RAB6C | 0,719 | 2,67E-09 | pt.cod | ENSG00000115355 | CCDC88A | -0,786 | 4,41E-11 | pt.cod |
| ENSG00000169583 | CLIC3 | 0,717 | 2,81E-09 | pt.cod | ENSG00000154127 | UBASH3B | -0,787 | 2,08E-13 | pt.cod |
| ENSG00000136367 | ZFHX2 | 0,716 | 1,69E-08 | pt.cod | ENSG00000171488 | LRRC8C | -0,788 | 7,11E-12 | pt.cod |
| ENSG00000176058 | TPRN | 0,716 | 4,64E-08 | pt.cod | ENSG00000147065 | MSN | -0,791 | 9,45E-13 | pt.cod |
| ENSG00000255020 | AF131216.5 | 0,715 | 3,72E-09 | AS | ENSG00000105971 | CAV2 | -0,793 | 1,72E-13 | pt.cod |
| ENSG00000145916 | RMND5B | 0,715 | 2,08E-08 | pt.cod | ENSG00000134954 | ETS1 | -0,83 | 1,11E-15 | pt.cod |
| ENSG00000133424 | LARGE | 0,714 | 1,84E-09 | pt.cod | ENSG00000185483 | ROR1 | -0,835 | 9,84E-15 | pt.cod |
| ENSG00000165215 | CLDN3 | 0,714 | 2,12E-09 | pt.cod | ENSG00000174130 | TLR6 | -0,838 | 6,40E-14 | pt.cod |
|  |  |  |  |  | ENSG00000135046 | ANXA1 | -0,839 | 1,79E-16 | pt.cod |

**Supplemental Table 5B: IPA** enriched functional annotations of DSCAM-AS1 correlated and anti-correlated genes. Genes are reported for each enriched annotation.

| **Functions Annotation** | **p-value** | **Molecules** |
| --- | --- | --- |
| cell movement of tumor cell lines | 2,39E-10 | ANXA1,CAV1,CCDC88A,CD44,CLDN3,CLU,CXCL2,CXCL3,CXCL8,DSE,ETS1,F7,FOXA1,HMGA2,IL6,ITGA6,LOX,MSN,NRP2,PREX1,PTGS2,RET,RHOB,ROR1,S1PR1,  SERPINB5,SLC9A3R1,SNAI2,SPDEF,TFF1,TNC |
| migration of tumor cell lines | 3,77E-09 | ANXA1,CAV1,CCDC88A,CD44,CLU,CXCL2,CXCL8,DSE,ETS1,F7,FOXA1,HMGA2,IL6,ITGA6,LOX,MSN,NRP2,PREX1,PTGS2,RHOB,ROR1,SLC9A3R1,SNAI2,SPDEF,TFF1,TNC |
| invasion of cells | 9,76E-09 | ANXA1,BATF3,CAV1,CCDC88A,CD44,CLDN3,CLU,CXCL8,DSE,ETS1,F7,FOXA1,HMGA2,IL6,ITGA6,LOX,NRP2,PTGS2,RET,RHOB,ROR1,SERPINB5,SERPINE2,SFRP1,  SLC9A3R1,SNAI2,SPDEF,TFF1,TFF3 |
| cell movement of tumor cells | 1,20E-08 | CAV1,CD44,CXCL8,IL6,PTGS2,RET,RHOB,S100A2,SERPINB5,SFRP1,SNAI2,SPDEF,TNC |
| invasion of tumor cell lines | 1,93E-08 | ANXA1,BATF3,CAV1,CCDC88A,CD44,CLDN3,CXCL8,DSE,ETS1,F7,FOXA1,IL6,LOX,NRP2,PTGS2,ROR1,SERPINB5,SERPINE2,SFRP1,SLC9A3R1,SNAI2,SPDEF,TFF1,TFF3 |
| organization of cytoplasm | 2,04E-08 | ADCY6,ANXA1,CACNA1H,CAV1,CAV2,CCDC88A,CD44,CIB3,CLU,CXCL8,DOPEY2,DZIP1L,ETS1,F7,FASN,FMNL2,GRIN1,GRM4,IFT140,IL6,ITGA6,KIRREL,LOX,MSN,NNMT,NRP2,  PACSIN1,PREX1,PRSS12,PTX3,RET,RHOB,RND1,ROR1,S1PR1,SEC16A,SERPINB5,SFRP1,SGK1,SLC9A3R1,TNC |
| proliferation of cells | 2,13E-08 | ABCC5,ADCY6,AKR1B1,ANXA1,B3GNT5,BICC1,BIRC3,CACFD1,CAV1,CAV2,CCDC88A,CD44,CISH,CLU,COL4A1,COL8A1,CORO1B,CXCL2,CXCL3,CXCL8,DEGS2,ETS1,F7,FASN,  FBP1,FOXA1,GPR3,GRM4,HMGA2,HPX,IFI16,IGF2BP3,IL6,IL7R,ITGA6,JAG1,LOX,LRRC26,MZB1,NNMT,NRP2,P3H2,PDCD1LG2,PRDM1,PTGS2,PTX3,RET,RGL2,RHOB,RHOH,  RND1,ROR1,S1PR1,SERPINB5,SERPINB7,SERPINE2,SFRP1,SGK1,SLC9A3R1,SNAI2,SOX13,SPDEF,STARD10,TFF1,TFF3,TLR6,TNC,TOB1,TPO,TRIM3,XBP1,ZG16B,ZMIZ1 |
| migration of tumor cells | 2,28E-08 | CAV1,CD44,CXCL8,F7,IL6,PTGS2,RET,RHOB,S100A2,SERPINB5,SFRP1,SNAI2,SPDEF,TNC |
| organization of cytoskeleton | 1,54E-07 | ADCY6,ANXA1,CACNA1H,CAV1,CCDC88A,CD44,CIB3,CLU,CXCL8,DZIP1L,ETS1,F7,FASN,FMNL2,GRIN1,GRM4,IFT140,IL6,ITGA6,KIRREL,LOX,MSN,NNMT,NRP2,PACSIN1,PREX1,  PRSS12,RET,RHOB,RND1,ROR1,S1PR1,SERPINB5,SFRP1,SGK1,SLC9A3R1,TNC |
| cell movement | 2,28E-07 | AKR1B1,ANXA1,CAV1,CCDC88A,CD44,CLDN3,CLU,COL4A1,CORO1B,CRIP2,CXCL2,CXCL3,CXCL8,CXXC5,DSE,ETS1,F7,FASN,FOXA1,GRIN1,HMGA2,IL6,ITGA6,JAG1,LOX,MSN,  NRP2,PDCD1LG2,PRDM1,PREX1,PTGS2,PTX3,RET,RHOB,ROR1,S100A2,S1PR1,SERPINB5,SERPINE2,SFRP1,SGK1,SLC9A3R1,SNAI2,SPDEF,TFF1,TFF3,TNC,ZG16B |
| migration of cells | 4,68E-07 | ANXA1,CAV1,CCDC88A,CD44,CLU,COL4A1,CORO1B,CRIP2,CXCL2,CXCL3,CXCL8,CXXC5,DSE,ETS1,F7,FASN,FOXA1,HMGA2,IL6,ITGA6,JAG1,LOX,MSN,NRP2,PDCD1LG2,PRDM1,  PREX1,PTGS2,PTX3,RET,RHOB,ROR1,S100A2,S1PR1,SERPINB5,SERPINE2,SFRP1,SGK1,SLC9A3R1,SNAI2,SPDEF,TFF1,TNC,ZG16B |
| synthesis of lipid | 1,43E-06 | ABAT,AKR1B1,ANXA1,B3GNT5,CACNA1H,CAV1,CAV2,CLU,CXCL3,CXCL8,DEGS2,FASN,FOXA1,IL6,ITGA6,LARGE,MZB1,PTGS2,PTX3,S1PR1,SERPINE2,SLC9A3R1,XBP1 |
| chemotaxis of cells | 1,56E-06 | ANXA1,CAV1,CCDC88A,CD44,CLU,CXCL2,CXCL3,CXCL8,F7,IL6,ITGA6,LOX,NRP2,PREX1,PTGS2,S1PR1,SNAI2,TFF1,TFF3 |
| migration of breast cancer cell lines | 1,62E-06 | ANXA1,CAV1,CCDC88A,CXCL8,ETS1,FOXA1,IL6,ITGA6,ROR1,SNAI2,SPDEF,TFF1 |
| cell movement of breast cancer cell lines | 1,64E-06 | ANXA1,CAV1,CCDC88A,CXCL8,ETS1,FOXA1,IL6,ITGA6,ROR1,SERPINB5,SNAI2,SPDEF,TFF1 |
| recruitment of cells | 1,66E-06 | ANXA1,CAV1,CD44,CLU,CXCL2,CXCL3,CXCL8,ETS1,IL6,PREX1,PTGS2,PTX3,S1PR1,SFRP1,TNC |
| synthesis of fatty acid | 1,72E-06 | ABAT,AKR1B1,ANXA1,CAV1,CAV2,CLU,CXCL3,CXCL8,FASN,FOXA1,IL6,PTGS2,S1PR1,XBP1 |
| differentiation of cells | 1,89E-06 | ADCY6,ALDH6A1,ANXA1,BATF3,CAV1,CD44,CISH,CLU,COL4A1,CXCL8,ETS1,FASN,FOXA1,GLIS3,HMGA2,HPX,IFI16,IL6,IL7R,ITGA6,JAG1,LRRC8C,MZB1,NNMT,PACSIN1,PRDM1,  PREX1,PRSS12,PTGS2,PTRF,RET,RGL2,RHOB,RHOH,RND1,ROR1,S1PR1,SERPINE2,SFRP1,SGK1,SNAI2,TFF1,TLR1,TLR6,TNC,TOB1,XBP1,ZBTB46 |
| microtubule dynamics | 2,30E-06 | ADCY6,CACNA1H,CAV1,CCDC88A,CD44,CIB3,CLU,CXCL8,DZIP1L,F7,FASN,GRIN1,GRM4,IFT140,IL6,ITGA6,LOX,MSN,NNMT,NRP2,PACSIN1,PREX1,PRSS12,RET,RHOB,RND1  ,ROR1,S1PR1,SGK1,SLC9A3R1,TNC |
| synthesis of prostaglandin E2 | 3,31E-06 | AKR1B1,ANXA1,CAV1,CLU,CXCL8,FASN,IL6,PTGS2,S1PR1 |
| cellular homeostasis | 3,46E-06 | ADCY6,AKR1B1,ANXA1,CACNA1H,CAV1,CISH,COL4A1,CXCL2,CXCL3,CXCL8,ETS1,F7,FOXA1,GPR3,GRIN1,HPX,IL6,IL7R,JAG1,KIAA1324,LARGE,P2RX2,PRDM1,PREX1,PTGS2,  RHOH,S1PR1,SFRP1,SGK1,SLC9A2,SLC9A3R1,SNAI2,TLR1,TLR6,XBP1,ZG16B |
| morphology of cells | 4,41E-06 | ANXA1,B3GNT5,CACNA1H,CAV1,CAV2,CD44,CISH,CLU,COL8A1,CREB3L4,CXCL8,DSE,ETS1,F7,FASN,GLIS3,GPR3,GRIN1,IL6,IL7R,ITGA6,KIAA1324,KIRREL,LOX,MAN1C1,MSN,  PACSIN1,PRDM1,PRSS12,RET,RHOB,RHOH,SERPINB5,SERPINE2,SFRP1,SLC9A2,SLC9A3R1,SNAI2,SPDEF,TNC,TOB1,XBP1,ZBTB46 |
| proliferation of prostate cancer cell lines | 4,86E-06 | CAV1,CD44,CLU,CXCL8,ETS1,FASN,FOXA1,HMGA2,IFI16,IL6,LRRC26,SERPINB5,XBP1 |
| colony formation | 5,13E-06 | ANXA1,CAV1,CD44,CLU,CXCL3,HMGA2,IFI16,IL6,LOX,PTGS2,PTX3,RET,RHOB,SFRP1,SNAI2,TFF1,TNC,XBP1 |
| inflammation of body region | 5,33E-06 | ANXA1,BIRC3,CAV1,CD44,CISH,CLU,CXCL2,CXCL3,CXCL8,DNASE1,HMGA2,HPX,IL6,JAG1,P2RX2,PDCD1LG2,PRDM1,PTGS2,RET,S1PR1,SLC9A2,SPDEF,TFF3,TLR1,TOB1,XBP1 |
| cell movement of cancer cells | 5,34E-06 | CAV1,CD44,CXCL8,IL6,RHOB,S100A2,SFRP1,SNAI2,TNC |
| cell movement of blood cells | 5,45E-06 | AKR1B1,ANXA1,CAV1,CCDC88A,CD44,CLU,COL4A1,CXCL2,CXCL3,CXCL8,ETS1,F7,IL6,ITGA6,NRP2,PDCD1LG2,PRDM1,PREX1,PTGS2,PTX3,RHOB,S1PR1,SFRP1,SPDEF,TNC |
| proliferation of blood cells | 7,00E-06 | ANXA1,B3GNT5,BIRC3,CAV1,CD44,CISH,CXCL2,CXCL3,CXCL8,ETS1,HPX,IL6,IL7R,JAG1,LOX,PDCD1LG2,PRDM1,PTGS2,RHOH,S1PR1,SNAI2,SOX13,TOB1,TPO |
| binding of cells | 7,44E-06 | CAV1,CD44,CXCL2,CXCL3,CXCL8,DSE,GRIN1,IL6,ITGA6,LOX,MSN,PTX3,RHOB,RHOH,S1PR1,SERPINB5,TNC |
| inflammation of organ | 9,53E-06 | ANXA1,CAV1,CD44,CISH,CLU,CXCL2,CXCL3,CXCL8,DNASE1,HMGA2,HPX,IL6,ITGA6,JAG1,MSN,P2RX2,PDCD1LG2,PRDM1,PTGS2,RET,S100A2,S1PR1,SERPINB5,SLC9A2,SPDEF,  TFF3,TLR1,TOB1,XBP1 |
| fibrogenesis | 1,24E-05 | CAV1,CD44,CIB3,CXCL8,CXXC5,F7,IL6,JAG1,KIRREL,PREX1,RHOB,RND1,S1PR1,SGK1,TNC |
| apoptosis of breast cancer cell lines | 1,25E-05 | BIRC3,CAV1,CD44,CLU,FASN,IFI16,PTGS2,SERPINB5,SFRP1,SGK1,SNAI2,XBP1 |
| inflammation of intestine | 1,25E-05 | ANXA1,CD44,CXCL2,CXCL3,CXCL8,IL6,PTGS2,S1PR1,SPDEF,TFF3,TLR1,XBP1 |
| activation of cells | 1,28E-05 | ANXA1,CD44,CISH,CXCL2,CXCL3,CXCL8,DNASE1,ETS1,F7,IFI16,IL6,PDCD1LG2,PRDM1,PREX1,PTGS2,RET,RHOB,RHOH,SERPINE2,SFRP1,SPDEF,TLR1,TLR6,TNC,TPO,ZBTB46 |
| leukocyte migration | 1,57E-05 | ANXA1,CAV1,CCDC88A,CD44,CLU,COL4A1,CXCL2,CXCL3,CXCL8,ETS1,F7,IL6,ITGA6,NRP2,PDCD1LG2,PRDM1,PREX1,PTGS2,PTX3,RHOB,S1PR1,SFRP1,SPDEF,TNC |
| organization of membrane rafts | 1,70E-05 | CAV1,CAV2,CD44 |
| necrosis | 1,74E-05 | ADCY6,AKR1B1,ANXA1,BIRC3,CACNA1H,CAV1,CD44,CISH,CLU,CNNM4,COL4A1,CXCL2,CXCL3,CXCL8,DNASE1,DPYD,ETS1,F7,FASN,GLIPR1,GLIS3,GRIN1,GRM4,HMGA2,  IFI16,IL6,IL7R,ITGA6,JAG1,MSN,MZB1,PDCD1LG2,PRDM1,PTGS2,RET,RHOB,RHOH,ROR1,S1PR1,SERPINB5,SERPINE2,SFRP1,SGK1,SNAI2,SYNGR2,TFF3,TLR1,TLR6,TNC,XBP1 |
| formation of cytoskeleton | 1,76E-05 | CAV1,CD44,CIB3,CXCL8,CXXC5,F7,JAG1,KIRREL,PREX1,RHOB,RND1,S1PR1,SGK1,TNC |
| immune response of cells | 1,78E-05 | ANXA1,BATF3,BIRC3,CAV1,CD44,CXCL3,CXCL8,ETS1,IL6,IL7R,PDCD1LG2,PRDM1,PTX3,RET,ROR1,SGK1,TLR1,XBP1 |
| cell movement of carcinoma cell lines | 2,06E-05 | CAV1,CD44,CXCL8,DSE,ETS1,HMGA2,NRP2,PTGS2 |
| migration of cervical cancer cell lines | 2,37E-05 | CCDC88A,CXCL2,ETS1,PREX1,PTGS2,SLC9A3R1 |
| colony formation of cells | 2,65E-05 | ANXA1,CAV1,CD44,CLU,CXCL3,HMGA2,IFI16,IL6,LOX,PTGS2,PTX3,RET,RHOB,SFRP1,SNAI2,TFF1 |
| synthesis of reactive oxygen species | 2,76E-05 | AKR1B1,ANXA1,CAV1,CD44,CXCL2,CXCL8,ETS1,F7,FBP1,GRIN1,IL6,PREX1,PTGS2,SERPINB5,TPO,XBP1 |
| infiltration of blood cells | 2,83E-05 | AKR1B1,ANXA1,CAV1,CD44,CXCL2,CXCL3,CXCL8,ETS1,IL6,PRDM1,PTGS2,PTX3,SFRP1,SPDEF |
| colony formation of tumor cell lines | 3,39E-05 | ANXA1,CAV1,CD44,CLU,HMGA2,IFI16,IL6,LOX,PTGS2,SNAI2,TFF1 |
| formation of filaments | 3,48E-05 | CAV1,CD44,CIB3,CXCL8,CXXC5,F7,JAG1,KIRREL,PREX1,RHOB,RND1,S1PR1,SGK1,TNC |
| tubulation of cells | 3,50E-05 | CAV1,CD44,CXCL8,IL6,RHOB,S1PR1,SNAI2,ZG16B |
| immune response of leukocytes | 3,50E-05 | ANXA1,BATF3,CD44,CXCL3,CXCL8,ETS1,IL6,IL7R,PDCD1LG2,PTX3,RET,XBP1 |
| synthesis of eicosanoid | 3,70E-05 | AKR1B1,ANXA1,CAV1,CLU,CXCL3,CXCL8,FASN,IL6,PTGS2,S1PR1 |
| adhesion of lymphoma cell lines | 3,93E-05 | ANXA1,CD44,CXCL2,IL6,ITGA6 |
| migration of carcinoma cell lines | 4,28E-05 | CAV1,CXCL8,DSE,ETS1,HMGA2,NRP2,PTGS2 |
| cell cycle progression of breast cancer cell lines | 4,35E-05 | CAV1,CLU,IL6,TNC,XBP1 |
| invasion of colon cancer cell lines | 4,64E-05 | ANXA1,CAV1,CD44,PTGS2,TFF1,TFF3 |
| cell movement of colon cancer cell lines | 5,03E-05 | CAV1,CCDC88A,CD44,CXCL8,F7,ITGA6,PTGS2 |
| transmigration of cells | 5,23E-05 | CD44,COL4A1,CXCL2,CXCL3,CXCL8,IL6,PDCD1LG2,TNC |
| inflammatory response | 5,43E-05 | ANXA1,BIRC3,CAV1,CCDC88A,CD44,CXCL2,CXCL3,CXCL8,ETS1,F7,IL6,PRDM1,PREX1,PTGS2,PTX3,S1PR1,SFRP1,TLR1,TLR6,TNC,XBP1 |
| development of cytoplasm | 5,46E-05 | ANXA1,CAV1,CD44,CIB3,CXCL8,CXXC5,F7,JAG1,KIRREL,PREX1,RHOB,RND1,S1PR1,SGK1,TNC |
| cell movement of prostate cancer cell lines | 5,59E-05 | CAV1,CLU,CXCL8,FOXA1,IL6,SERPINB5,SPDEF |
| formation of caveolae | 5,73E-05 | CAV1,CAV2,PTRF |
| migration of prostate cancer cells | 5,73E-05 | CAV1,RHOB,SNAI2 |
| migration of squamous cell carcinoma cell lines | 5,81E-05 | CD44,DSE,IL6,PTGS2,SNAI2 |
| adhesion of tumor cell lines | 6,02E-05 | ANXA1,CAV1,CD44,CXCL2,CXCL8,ETS1,IL6,ITGA6,SERPINB5,TFF3,TNC |
| branching of cells | 6,04E-05 | ADCY6,CAV1,ETS1,IL6,NNMT,PACSIN1,PRSS12,RET,RHOB,RND1,ROR1,SGK1,TNC |
| fatty acid metabolism | 6,26E-05 | ABAT,AKR1B1,ANXA1,CAV1,CAV2,CLU,CXCL3,CXCL8,DEGS2,FASN,FOXA1,IL6,LARGE,PTGS2,S1PR1,SLC9A3R1,XBP1 |
| cell viability of breast cancer cell lines | 6,46E-05 | CD44,CLU,CXCL2,CXCL3,HMGA2,IGF2BP3,PTGS2,SNAI2 |
| invasion of breast cancer cell lines | 6,79E-05 | CAV1,CCDC88A,CD44,FOXA1,LOX,PTGS2,ROR1,SERPINB5,SLC9A3R1,TFF1 |
| adhesion of connective tissue cells | 7,32E-05 | CD44,CLU,IL6,JAG1,RHOB,RND1,SERPINB5,TNC |
| binding of leukemia cell lines | 7,63E-05 | CD44,IL6,ITGA6,RHOH,TNC |
| influx of cells | 7,88E-05 | CD44,CXCL2,CXCL3,IL6,PTX3,SGK1 |
| binding of DNA | 8,18E-05 | ADCY6,CAV1,CISH,CLU,CXCL8,DNASE1,ETS1,FOXA1,IFI16,IL6,LOX,PRDM1,RHOB,S1PR1,SNAI2,TFF3 |
| migration of colon cancer cell lines | 9,48E-05 | CAV1,CCDC88A,CD44,CXCL8,F7,ITGA6 |
| apoptosis of neutrophils | 9,86E-05 | ANXA1,CD44,CXCL2,CXCL8,IL6 |
| sprouting | 1,00E-04 | CAV1,ETS1,IL6,NNMT,PACSIN1,PRSS12,RET,RHOB,RND1,ROR1,SGK1,TNC,ZMIZ1 |
| proliferation of tumor cell lines | 1,02E-04 | ANXA1,CAV1,CCDC88A,CD44,CLU,CXCL8,DEGS2,ETS1,F7,FASN,FOXA1,HMGA2,IFI16,IGF2BP3,IL6,ITGA6,JAG1,LOX,LRRC26,NRP2,PRDM1,PTGS2,RET,RGL2,  RHOB,ROR1,SERPINB5,SFRP1,SGK1,SLC9A3R1,XBP1,ZMIZ1 |
| necrosis of epithelial tissue | 1,05E-04 | BIRC3,CAV1,CD44,CLU,CNNM4,CXCL3,CXCL8,IL6,MZB1,PTGS2,RHOB,S1PR1,SERPINB5,SFRP1,SGK1,TFF3,XBP1 |
| formation of plasma membrane | 1,06E-04 | CAV1,CLDN3,COL4A1,CXCL8,F7,GRIN1,ITGA6,P2RX2,S1PR1,TNC |
| cell death of epithelial cells | 1,36E-04 | BIRC3,CD44,CLU,CNNM4,CXCL3,CXCL8,IL6,MZB1,PTGS2,S1PR1,SERPINB5,SFRP1,SGK1,TFF3,XBP1 |
| cell viability of leukocytes | 1,48E-04 | CD44,CISH,CLU,CXCL8,IL6,IL7R,PDCD1LG2,PRDM1,SNAI2,XBP1 |
| quantity of metal ion | 1,49E-04 | ADCY6,AKR1B1,CACNA1H,CANT1,CAV1,CXCL3,CXCL8,FOXA1,GRIN1,IL6,PTGS2,S1PR1,SGK1,SLC9A3R1 |
| arrest in cell cycle progression of tumor cell lines | 1,52E-04 | CAV1,CLU,FOXA1,IFI16,IL6,TNC,XBP1 |
| apoptosis | 1,65E-04 | ADCY6,AKR1B1,ANXA1,BIRC3,CACFD1,CAV1,CCDC88A,CD44,CLU,COL4A1,CXCL2,CXCL8,DNASE1,ETS1,F7,FASN,GLIPR1,GLIS3,GRIN1,GRM4,HMGA2,IFI16,  IL6,IL7R,ITGA6,JAG1,MSN,MZB1,PDCD1LG2,PRDM1,PTGS2,RET,RHOB,RHOH,ROR1,S1PR1,SERPINB5,SERPINE2,SFRP1,SGK1,SNAI2,SPDEF,TFF1,TFF3,TLR1,TLR6,TNC,XBP1 |
| quantity of metal | 1,65E-04 | ADCY6,AKR1B1,CACNA1H,CANT1,CAV1,CXCL3,CXCL8,FOXA1,GRIN1,HPX,IL6,PTGS2,S1PR1,SGK1,SLC9A3R1 |
| ion homeostasis of cells | 1,77E-04 | ADCY6,AKR1B1,ANXA1,CAV1,CXCL2,CXCL3,CXCL8,F7,GPR3,GRIN1,HPX,IL6,P2RX2,S1PR1,SGK1,SLC9A3R1 |
| apoptosis of prostate cancer cell lines | 1,81E-04 | BIRC3,CAV1,CLU,FASN,GLIPR1,IL6,PTGS2,SERPINB5 |
| adhesion of breast cancer cell lines | 1,83E-04 | ANXA1,CD44,ETS1,IL6,SERPINB5 |
| binding of tumor cell lines | 1,87E-04 | CD44,CXCL3,DSE,IL6,ITGA6,RHOH,SERPINB5,TNC |
| cell movement of hairy leukemia cells | 1,89E-04 | CD44,CXCL8 |
| catabolism of thymine | 1,89E-04 | ALDH6A1,DPYD |
| engulfment of tumor cell lines | 2,04E-04 | ANXA1,CAV1,CD44,CLIC3,IL6,PTX3,ZG16B |
| secretion of fatty acid | 2,10E-04 | ANXA1,CXCL8,GRM4,PTGS2,RET |
| cell death | 2,24E-04 | ABCC5,ADCY6,AKR1B1,ANXA1,BATF3,BIRC3,CACFD1,CACNA1H,CAV1,CCDC88A,CD44,CISH,CLDN3,CLU,CNNM4,COL4A1,CXCL2,CXCL3,CXCL8,DNASE1,  DPYD,ETS1,F7,FASN,GLIPR1,GLIS3,GRIN1,GRM4,HMGA2,IFI16,IL6,IL7R,ITGA6,JAG1,MSN,MZB1,PDCD1LG2,PRDM1,PTGS2,RET,RHOB,RHOH,ROR1,S1PR1,  SERPINB5,SERPINE2,SFRP1,SGK1,SNAI2,SPDEF,SYNGR2,TFF1,TFF3,TLR1,TLR6,TNC,XBP1 |
| degranulation of cells | 2,27E-04 | ANXA1,CORO1B,CXCL3,CXCL8,ETS1,GRIN1,SGK1,SLC9A3R1,UBASH3B |
| inflammation of body cavity | 2,34E-04 | ANXA1,CAV1,CD44,CISH,CLU,CXCL2,CXCL3,CXCL8,DNASE1,HMGA2,HPX,IL6,P2RX2,PRDM1,PTGS2,RET,SLC9A2,SPDEF |
| synthesis of leukotriene | 2,41E-04 | ANXA1,CAV1,CXCL3,CXCL8,PTGS2 |
| cytolysis | 2,49E-04 | ANXA1,BATF3,CAV1,CD44,CLDN3,ETS1,F7,IL6,IL7R,TLR1 |
| production of reactive oxygen species | 2,50E-04 | AKR1B1,ANXA1,CAV1,CD44,CXCL2,CXCL8,ETS1,F7,FBP1,IL6,PTGS2,XBP1 |
| arrest in cell cycle progression | 2,91E-04 | CAV1,CD44,CLU,CXCL8,FASN,FOXA1,IFI16,IL6,PDCD1LG2,TNC,XBP1 |
| aggregation of cells | 2,91E-04 | AKR1B1,CANT1,CD44,CLU,IL6,PTGS2,PTX3,RET,SERPINE2,SGK1,UBASH3B |
| homing of tumor cell lines | 2,91E-04 | CD44,CXCL3,CXCL8,ITGA6,S1PR1,SNAI2,TFF1 |
| trafficking of cells | 2,94E-04 | ANXA1,BATF3,CD44,CXCL3,S1PR1 |
| binding of neuroglia | 3,12E-04 | CAV1,CD44,ITGA6 |
| chemotaxis of central nervous system cells | 3,12E-04 | CAV1,IL6,NRP2 |
| synthesis of hydrocortisone | 3,12E-04 | CACNA1H,FOXA1,IL6 |
| invasion of ovarian cancer cell lines | 3,13E-04 | CAV1,CD44,CLDN3,ETS1 |
| adhesion of blood cells | 3,24E-04 | ANXA1,CD44,CLU,CXCL2,CXCL3,CXCL8,IL6,ITGA6,MSN,PTGS2,PTX3,RHOB |
| apoptosis of tumor cell lines | 3,32E-04 | AKR1B1,BIRC3,CAV1,CD44,CLU,COL4A1,CXCL8,ETS1,FASN,GLIPR1,GLIS3,IFI16,IL6,JAG1,MSN,PRDM1,PTGS2,RET,RHOB,ROR1,SERPINB5,SFRP1,SGK1,SNAI2,TFF3,XBP1 |
| anoikis | 3,37E-04 | BIRC3,CAV1,COL4A1,PTGS2,RHOB,TNC |
| size of cells | 3,37E-04 | ANXA1,CAV2,CD44,FASN,GLIS3,IL6,IL7R,MSN,PACSIN1,PRDM1,RET,TOB1,XBP1 |
| formation of focal adhesions | 3,38E-04 | CAV1,DSE,JAG1,PTGS2,RHOB,RND1,S1PR1 |
| cell cycle progression of tumor cell lines | 3,63E-04 | CAV1,CD44,CLU,FOXA1,GLIPR1,IFI16,IL6,TNC,XBP1 |
| binding of squamous cell carcinoma cell lines | 3,75E-04 | CD44,DSE |
| binding of CRE-like element | 3,75E-04 | ADCY6,IL6 |
| apoptosis of synovial cells | 3,77E-04 | CD44,CLU,S1PR1,SNAI2 |
| apoptosis of epithelial cells | 3,94E-04 | CLU,CXCL8,IL6,PTGS2,S1PR1,SERPINB5,SFRP1,SGK1,TFF3,XBP1 |
| cell viability of mononuclear leukocytes | 3,94E-04 | CD44,CISH,CLU,CXCL8,IL6,IL7R,PRDM1,SNAI2 |
| morphology of tumor cell lines | 3,94E-04 | CLU,FASN,IL6,RET,SERPINB5,SNAI2,SPDEF,TNC |
| flux of Ca2+ | 3,94E-04 | ANXA1,CAV1,CXCL3,CXCL8,F7,GRIN1,IL6,P2RX2,S1PR1,SGK1 |
| cytostasis of tumor cell lines | 4,21E-04 | CAV1,CLU,ETS1,IFI16,IL6,JAG1,SERPINB5 |
| internalization by tumor cell lines | 4,23E-04 | ANXA1,CAV1,CLIC3,IL6,PTX3,ZG16B |
| cell death of immune cells | 4,54E-04 | ANXA1,BIRC3,CAV1,CD44,CISH,CLU,CXCL2,CXCL8,ETS1,IL6,IL7R,MZB1,PDCD1LG2,PRDM1,RHOH,SNAI2,XBP1 |
| cell viability | 4,85E-04 | CAV1,CD44,CISH,CLDN3,CLU,CXCL2,CXCL3,CXCL8,DPYD,F7,HMGA2,IGF2BP3,IL6,IL7R,JAG1,KIRREL,PDCD1LG2,PRDM1,PTGS2,RET,ROR1,S1PR1,SERPINB5,SFRP1,  SGK1,SNAI2,XBP1 |
| cell movement of mammary tumor cells | 4,89E-04 | CD44,RET,SERPINB5,SPDEF |
| organization of actin cytoskeleton | 5,41E-04 | CCDC88A,CLU,FMNL2,MSN,RET,RHOB,RND1,S1PR1,SFRP1,SLC9A3R1 |
| immune response of T lymphocytes | 5,47E-04 | CXCL8,ETS1,IL6,IL7R,PDCD1LG2,RET |
| inflammation of central nervous system | 5,77E-04 | CD44,HPX,IL6,JAG1,PDCD1LG2,PRDM1,PTGS2,S1PR1,TOB1,XBP1 |
| apoptosis of fibroblast-like synoviocytes | 5,95E-04 | CLU,S1PR1,SNAI2 |
| binding of prostate cancer cell lines | 5,95E-04 | CD44,ITGA6,SERPINB5 |
| synthesis of long chain fatty acid | 5,95E-04 | CAV1,CAV2,FASN |
| synthesis of oleic acid | 6,22E-04 | CAV1,CAV2 |
| secretion of eicosanoid | 6,24E-04 | ANXA1,CXCL8,PTGS2,RET |
| cell death of tumor cell lines | 6,58E-04 | AKR1B1,BIRC3,CACNA1H,CAV1,CD44,CLU,COL4A1,CXCL8,DNASE1,DPYD,ETS1,FASN,GLIPR1,GLIS3,IFI16,IL6,JAG1,MSN,PRDM1,PTGS2,RET,RHOB,ROR1,  SERPINB5,SFRP1,SGK1,SNAI2,SYNGR2,TFF3,XBP1 |
| formation of connective tissue cells | 6,62E-04 | CD44,CXCL8,IL6,IL7R,PRDM1,PTGS2,SFRP1,XBP1 |
| mitogenesis | 6,76E-04 | IL6,IL7R,PTGS2,PTX3,RET,SERPINB5,TNC |
| morphology of prostate cancer cell lines | 6,85E-04 | CLU,FASN,SPDEF |
| inflammation of stomach | 6,85E-04 | PTGS2,SLC9A2,SPDEF |
| cell-cell adhesion | 7,38E-04 | ANXA9,CD44,CLDN3,CXCL8,IGSF5,ITGA6,KIRREL,RET |
| metabolism of nucleic acid component or derivative | 7,38E-04 | ADCY6,ALDH6A1,CAV1,DPYD,ENTPD8,FASN,FBP1,GPD1L,GPR3,IL6,MZB1,NNMT,PTGS2,RGS20 |
| synthesis of steroid | 7,48E-04 | CACNA1H,CAV1,CXCL8,FOXA1,IL6,ITGA6,MZB1,SERPINE2,SLC9A3R1 |
| organization of endoplasmic reticulum | 7,83E-04 | CAV2,DOPEY2,SEC16A |
| chemokinesis of cells | 7,83E-04 | CXCL3,CXCL8,TFF1 |
| synthesis of platelet activating factor | 8,90E-04 | CXCL8,PTGS2,PTX3 |
| linkage of cytoskeleton | 9,28E-04 | GRIN1,MSN |
| synthesis of nucleotide | 9,43E-04 | ADCY6,CAV1,ENTPD8,FASN,FBP1,GPR3,IL6,MZB1,NNMT,PTGS2,RGS20 |
| organization of cellular membrane | 9,46E-04 | ANXA1,CAV1,CAV2,CCDC88A,CD44,CORO1B |
| cell movement of leukemia cell lines | 9,81E-04 | CD44,CXCL8,S1PR1,SLC9A3R1,SNAI2,TNC |

**Supplemental Table 6: Custom Primers sequences.** Expression and ChIP custom primer pairs are listed as forward (Fwd) and reverse (Rev) sequences.

| **Custom EXPRESSION PRIMERS** |  |  | FIREFLY luciferase | Fwd: 5'-CTGGATCTACTGGTCTGCCTAAA-3' |
| --- | --- | --- | --- | --- |
| DSCAM-AS1 | Fwd: 5'-GATCCTTGTTTGGTCTCACTCC-3' |  |  | Rev: 5'-AATCTCACGCAGGCAGTTCT-3' |
|  | Rev: 5'-ATGCCTATGTGGGTGATTGG-3' |  | RENILLA luciferase | Fwd: 5'-AACACCGAGTTCGTGAAGGT-3' |
| DSCAM-AS1 1n nuclear | Fwd: 5'-TTCTCAGCACGTTTTTGCAG-3' |  |  | Rev: 5'-CATTTCATCTGGAGCGTCCT-3' |
|  | Rev: 5'-CCGATCCGTCGTCCATCTC-3' |  | 14S | Fwd: 5'-TTCTCAGCACGTTTTTGCAG-3' |
| DSCAM-AS1 2c cytoplasmic | Fwd: 5'-CCACTGATGGGAAAGCCACA-3' |  |  | Rev: 5'-CCGATCCGTCGTCCATCTC-3' |
|  | Rev: 5'-GTGGAGGCACCTAAGTCAGT-3' |  | Vimentin | Fwd: 5'-ACCAGCTAACCAACGACAAAG-3' |
| DSCAM-AS1 3c cytoplasmic | Fwd: 5'-GTATGCAGCTGATAAGACGCT-3' |  |  | Rev: 5'-GCATCTCCTCCTGCAATTTT-3' |
|  | Rev: 5'-TGAGATGGGGTTTCGCTCTT-3' |  | **Custom ChIP PRIMERS** |  |
| DSCAM-AS1 4c cytoplasmic | Fwd: 5'-TTCTCAGCACGTTTTTGCAG-3' |  | DSCAM-AS1 | Fwd: 5'-CCACTCCACTGCCTTTTGGT-3' |
|  | Rev: 5'-CCGATCCGTCGTCCATCTC-3' |  |  | Rev: 5'-TGCAAGGCTCTGTGACATGAA-3' |
| LINC01016 | Fwd: 5'-CGCAGAAGACAGACCGGTAG-3' |  | LINC01016 | Fwd: 5'-GACTGCACTGGAGAGTGAGG-3' |
|  | Rev: 5'-GGGCCAAACTGCACTTCTT-3' |  |  | Rev: 5'-TTCGAAGCAGCCTGTGAAGT-3' |
| DANCR | Fwd: 5'-CGCCTCTCTGGTTTGTGC-3' |  | AP001057.1 | Fwd: 5'-CTAAGAGATGGGTGGCTGGAG-3' |
|  | Rev: 5'-CCGATCCGTCGTCCATCTC-3' |  |  | Rev: 5'-GAGAAGCACGCACATCCTGC-3' |
| AC068580.6 | Fwd: 5'-AGGTTCCAGAATGGGGAAGA-3' |  | TFF1 | Fwd: 5'-CACCCCGTGAGCCACTGT-3' |
|  | Rev: 5'-CCCGAGGTGCTCAAGAACT-3' |  |  | Rev: 5'-CTGCAGAAGTGATTCATAGTGAGAGAT-3' |
| RP11.320N21.1 | Fwd: 5'-TTAGGACACCAGGCAGAAAGTT-3' |  | KCNQ1OT1 | Fwd: 5'-CCCAGGGAGTTCATGTGTCT-3' |
|  | Rev: 5'-AATGCCCAAGGCTGTCAT-3' |  | (control negative) | Rev: 5'-TGACAAAAACTTCCCAGCTAGA-3' |

**Supplemental Table 7: FACS analysis of MCF7 Cell Cycle upon DSCAM-AS1 silencing.** The table reports the percentage of cells in different cell cycle phases or in the apoptotic fraction as defined by FACS analysis. The result for cell grown in full or hormone deprived medium and transfected with control siRNA (siCTR) or with two different siRNAs targeting DSCAM-AS1, alone (siR_1 and siR_2) or in combination (siR_1+2) are reported

| **Full Medium** | **% of apoptotic cells** | **% of cells in G0/G1 phase** | **% of cells in S phase** | **% of cells in G2/M phase** |
| --- | --- | --- | --- | --- |
| **siCTR** | 22,39 | 51,91667 | 9,133333 | 17,35667 |
| **siR_1** | 28,05333 | 49,59333 | 8,08 | 15,02667 |
| **siR_2** | 28,52333 | 48,14333 | 8,83 | 15,38 |
| **siR_1+2** | 28,27667 | 50,42 | 8,556667 | 13,64 |
| **Hormone-Depleted Medium** |  |  |  |  |
| **siCTR** | 29,52 | 50,27 | 8,016667 | 13,01667 |
| **siR_1** | 38,69333 | 42,64 | 7,49 | 12,14333 |
| **siR_2** | 41,48333 | 40,86667 | 6,683333 | 11,67333 |
| **siR_1+2** | 35,86333 | 41,27667 | 5,7 | 11,08 |
